# Storytelling in family-provided care - developing diversity-sensitive stories for family caregivers of Turkish individuals living with dementia

**DOI:** 10.1186/s12912-025-03712-7

**Published:** 2025-08-12

**Authors:** Kübra Annac, Ela Rana Örs, Sümeyra Öztürk, Mualla Basyigit, Tugba Aksakal, Christina Kuhn, Anja Rutenkröger, Hürrem Tezcan-Güntekin, Yüce Yilmaz-Aslan, Patrick Brzoska

**Affiliations:** 1 Faculty of Health/School of Medicine, Health Services Research Unit, Witten/Herdecke University, Alfred-Herrhausen-Straße 50, 58455 Witten, Germany; 2 Alice Salomon University Berlin, Berlin School of Public Health, Berlin, Germany; 3 Demenz Support Stuttgart gGmbH, Stuttgart, Germany

**Keywords:** Storytelling, Family-provided care, Family caregivers, Dementia, Diversity, Migration

## Abstract

**Background:**

Family caregivers play a crucial role in care for individuals with dementia, particularly in migrant communities such as families of Turkish descent in Germany. However, caregivers often experience high levels of stress due to cultural expectations, family dynamics, and limited access to support services. Traditional self-help approaches frequently fail to address their diverse needs, necessitating innovative strategies to enhance self-management skills and emotional resilience. Storytelling, a method that facilitates knowledge transfer and emotional expression through narratives, has the potential to empower caregivers by fostering empathy, self-reflection, and problem-solving skills. This study aims to develop and evaluate storytelling interventions that are culturally and linguistically tailored to support Turkish family caregivers in managing caregiving challenges more effectively.

**Methods:**

A multi-stage methodological approach was used to develop a diversity-sensitive storytelling intervention for Turkish family caregivers of individuals with dementia. The process included a comprehensive needs assessment through a literature review and qualitative secondary data analysis of interviews. Based on these insights, different caregiver personas were identified, each representing different challenges and caregiving roles. Stories were then developed following principles of effective storytelling, incorporating key themes such as stress management, family conflict resolution, and cultural expectations. A quality assessment was conducted using a pretest with caregivers, applying the Community-Based Participatory Research (CBPR) approach to refine narratives based on clarity, accessibility, and relatability.

**Results:**

The study resulted in the creation of nine storytelling packs, each containing five stories tailored to specific caregiver personas. These narratives were developed in German and translated into Turkish and English to ensure linguistic and cultural accessibility. Pretests indicated that the stories resonated with caregivers, addressing their unique caregiving realities while fostering engagement and perceived emotional support. Feedback led to refinements in language style and narrative structure, ensuring greater clarity and identification with the protagonists.

**Conclusion:**

Storytelling shows promise as a supportive intervention for Turkish family caregivers of individuals with dementia by fostering self-management skills and reducing emotional burden. The developed stories provide an inclusive, diversity sensitive approach to addressing caregivers’ challenges, promoting self-reflection, and enhancing support networks. Future research should explore the long-term impact of storytelling interventions and their integration into caregiver support programs to improve well-being and caregiving outcomes.

**Supplementary Information:**

The online version contains supplementary material available at 10.1186/s12912-025-03712-7.

## Background

A considerable number of individuals requiring care in Germany receive care at home from family members and close relatives [1]. Among individuals with a Turkish migration background, the proportion of those receiving care at home is nearly 100% [2] Providing care for a relative can result in both physical and psychological stress for caregivers [3]. Approximately half of all family caregivers experience mental illness related to their caregiving responsibilities and the associated stress [4]. These burdens vary depending on factors such as age, gender, generational relationship, cultural background, and socio-economic conditions [5].

In order to guarantee the continuity of care at home, it is essential to reinforce the resources available to caregivers and alleviate the burden on them. One potential avenue for providing relief is by promoting the self-management skills of family caregivers, thereby supporting them in assuming responsibility for their own actions [6, 7]. Strengthening resources can help caregivers to meet the challenges resulting from caregiving and to expand their self-management competencies [8].

Nevertheless, specific demographic groups, including those with a history of migration, seldom engage in suitable support programmes, such as conventional self-help groups [9]. One reason for this is that significant differences between participants in such a self-help group in terms of age, biography, relationship to the person in need of care, religiosity, language skills, sexual orientation, level of education and care-related challenges can lead to a reduction in identification of the family caregivers with the other group members. This subsequently results in discontinuities in participation or dropping out of the group if the aforementioned factors are not adequately addressed [10]. Interventions to promote self-management skills must take into account the individual resources of those affected as well as their social and cultural diversity [11, 12]. Diverse characteristics interact with each other, a phenomenon that can be examined through intersectional analyses. This approach permits the examination of how multiple diversity attributes can intensify inequalities. An intersectional perspective is often limited in the domains of prevention, health promotion, and nursing science. In particular, the interplay between migration and other diversity attributes has not been sufficiently investigated. It is postulated that the coexistence of multiple attributes, each of which may independently precipitate significant psychological distress and related health complications, may exacerbate the effects of stress and the risk of illness [13]. To date, there is a paucity of studies that adopt an intersectional perspective and examine a broader range of differentiating characteristics beyond migration history, such as socio-economic status, sexual orientation or identity, and family role models [14, 15]. Migrant caregivers often face specific barriers that cannot be fully captured by considering individual diversity characteristics alone. An intersectional perspective is needed to examine the increased inequality or disadvantage that may result from the interaction of multiple diversity characteristics. In addition to structural barriers, caregivers with a Turkish migration background often encounter discriminatory attitudes and cultural stereotypes. These experiences can reduce trust in the healthcare system, discourage participation in support services, and reinforce existing inequalities [16, 17].

One technique that allows for such a perspective is storytelling. Storytelling is a concept based on the use of stories to convey knowledge, values and emotions. Greenhalgh describes storytelling as a method that makes complex issues understandable and accessible, using narratives (stories) to foster interpersonal connections and empathy [18]. Storytelling enables participants to share experiences and emotions through personal stories, which can help to promote wellbeing, particularly in a healthcare context. The utilisation of storytelling has the potential to enhance the development of self-management abilities, health literacy and self-efficacy expectations. The method entails the moderated presentation of narratives on a range of subjects, with the objective of fostering individuals’ capacity to disseminate knowledge, experience and information autonomously [19]. The mechanism through which storytelling can promote self-management can be understood through the lens of the Health Belief Model (HBM). This model suggests that individuals are more likely to engage in health-promoting behavior when they perceive a personal susceptibility and severity regarding a health issue, believe in the effectiveness of the proposed action, and feel capable of taking that action [20]. Storytelling supports this process by embedding health-relevant knowledge, perceived coping strategies, and positive examples of caregiver behavior within emotionally resonant narratives [21]. Through identification with protagonists and situations, caregivers may reassess their perceived burden, develop greater self-efficacy, and be motivated to adopt supportive caregiving behaviors. In this way, storytelling fosters both emotional engagement and cognitive appraisal, creating a bridge between awareness and action in self-management [22].

Storytelling aligns with an intersectional approach by representing diverse caregiving experiences shaped by factors such as migration background, socio-economic status, gender, family structure, and language. By incorporating intersectionality, storytelling addresses the unique challenges of caregivers, ensuring their varied needs are recognized. This approach leads to more inclusive and effective support, enhancing the relevance and accessibility of caregiving resources [23].

The use of storytelling in the context of family-provided care for Turkish individuals in Germany was first investigated in 2011 [24]. The narrative-generating methodology was particularly effective in facilitating the exchange of knowledge, experiences and information between family caregivers. The approach enabled valuable insights to be gained regarding the removal of access barriers, the utilisation of support services, the strengthening of the self-help potential of family caregivers and the necessity for the participation of the target group in the design of such services [24]. Through storytelling, caregivers’ experiences that might otherwise remain unspoken can be expressed in a safe environment. The decision to use storytelling as a central concept is based on its ability to create an emotional and identifying connection between those involved. The utilisation of stories is particularly well-suited to addressing the emotional and practical challenges frequently experienced by caregivers, thereby facilitating their identification with the protagonists in the stories and enabling the acquisition of knowledge from them. Particularly for caregivers who often suffer from social isolation, storytelling provides an opportunity to reflect on their experiences and to feel understood. This approach helps to reduce the burden on caregivers by fostering a supportive community [16, 17].

Storytelling as a method can take different forms. It can involve individuals sharing their own personal experiences in their own words, providing authentic insights and emotional connection. Alternatively, storytelling can use fictional or composite stories created by researchers or facilitators as prompts to spark discussion and reflection. Both approaches help explore perspectives and ideas, either through real-life narratives or imagined scenarios, depending on the objective. In this study, storytelling refers to the use of researcher-developed narratives, designed to reflect diverse caregiving experiences and challenges. The narratives serve as tools to stimulate caregivers’ reflection and discussion, fostering emotional connection, identification, and empowerment. Through engagement with these stories, caregivers can gain insights, enhance self-management skills, and feel supported in their caregiving role.

Although the use of researcher-developed narratives appear helpful, there is a lack of suitable stories within the context of storytelling that address the care and living environment of caregivers of Turkish individuals living with dementia. Consequently, there is a need for the development of stories that authentically reflect the multifarious disadvantages experienced by the target group, with a view to enhancing the quality of care.

The present study addresses this limitation. The aim of this study is to describe the development of diversity-sensitive narratives designed for use in storytelling interventions for family caregivers of Turkish individuals living with dementia, with the goal of enhancing caregivers’ self-management skills.

## Methods

**Fig. 1:**
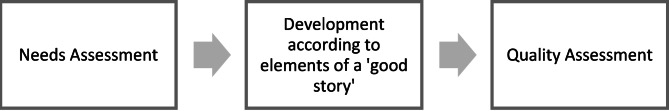
Methodological process for the development of diversity-sensitive stories for caregivers of Turkish individuals living with dementia

The development of the stories followed a methodologically coordinated, multi-stage process that combined formative needs research with the systematic use of collected data [25, 26]. This approach integrated a needs assessment based on a literature review and qualitative secondary data analysis, and the application of narrative principles associated with effective storytelling, followed by a structured quality assessment (Fig. 1). The chosen methods were intended to support the creation of stories that are both practical and diversity-sensitive, while aligning with the real-world needs of family caregivers of individuals living with dementia. All authors of this article actively participated in the development process. The project team brings together interdisciplinary expertise in intersectionality, dementia, family caregiving, diversity-sensitive healthcare, and practical experience in close contact with patients and their families. This collaborative approach ensured that the selected themes were both comprehensive and grounded in real-world caregiving contexts. The following sections describe each step in greater detail and outline its specific contribution to the story development process.

### Needs assessment

Prior to the development of any given story, it is imperative to identify extant literature and undertake a comprehensive assessment of the needs of the target group. This process ensures that the stories are tailored to the specific experiences, challenges, and requirements of the intended audience. To this end, a literature review was carried out alongside a qualitative secondary data analysis of interviews with family caregivers of Turkish individuals living with dementia. The goal was to synthesize existing knowledge on the diverse needs of caregivers and to highlight gaps that the stories should address [27, 28]. In addition to the literature review, a qualitative secondary data analysis was conducted using 31 interviews from three studies that focused on family caregivers of Turkish individuals living with dementia [10, 29, 30]. The analysis employed Winker and Degele’s multi-level approach [15], which examines social structures, identity constructs, and interaction processes. This framework enabled a nuanced understanding of how cultural, social, and individual factors shape the caregiving experience.

By integrating findings from the literature review and qualitative secondary data analysis, this needs assessment ensures that the developed stories authentically reflect the challenges, needs, and lived realities of caregivers, ultimately contributing to a more meaningful and impactful storytelling approach.

### Development according to elements of a good story

The findings of the needs assessment served as the foundation for designing stories that are both evidence-informed and practically relevant. This process ensured that the stories were not only tailored to the specific needs of the target audience but also grounded in scientific understanding to enhance their impact. Accordingly, the initial development of the stories was guided by the core principles of effective storytelling, as described by Holzinger and Sturmer [31] (Table 1).

**Table 1:** The fundamental elements of a ‘good story’ according to Holzinger and Sturmer [31]

Elements	Description
Message	What do you want to achieve with your story? Generate sympathy or disgust? Do you want to make your users laugh or cry? The message is essential for the choice of protagonists and the tonality of the language.
Protagonists	Every story lives through its protagonists. The protagonists must be connected to the theme, with experiences and feelings that are as intense as possible.
Plot	Stories have a beginning and an end; and a middle where the story can develop. What is the thread that can pull the user through the whole plot?
Setting	Where does your story take place? Good descriptions allow users to get an idea of the setting and understand the context of the story.
Dramaturgy	When researching a topic, look for dramatic moments. Are there turning points, biographical breaks or even conflicts? Conflict is the driving force behind stories.
Narrator	Narrators create perspective. Narrators determine the point of view and thus the perception of the story. First-person narrators can also be compelling.
Language style	The language used should reflect the desired atmosphere and be appropriate for the target audience.

The effectiveness of the stories lies in their deliberate use of key storytelling elements, each carefully selected to serve a specific function and foster meaningful engagement with the target audience. During the development process, these components, characteristics of a ‘good story’, were systematically incorporated to ensure narrative quality and impact. The following section outlines how each storytelling element was applied and highlights its specific role in shaping the final stories.

#### Message

The stories are written with a clear and focused message aimed at eliciting compassion and understanding from the readers. Each story is designed to shed light on the challenges faced by caregivers while promoting solutions such as seeking support and embracing self-help. By focusing on these aspects, the stories inspire empathy and action.

#### Protagonists

Protagonists form the emotional core of the stories, reflecting a wide range of diversity characteristics and life experiences. By presenting protagonists connected to the theme with intense emotions and relatable experiences, the stories enable readers to form an emotional connection and foster identification with the protagonists. The protagonists were chosen to reflect different types of caregivers identified in the needs assessment. The protagonists illustrate diverse dimensions of caregiving, highlighting variations in roles, family dynamics, beliefs, and traditions. They range from individuals providing care alone to those supported by siblings or extended family. The stories depict different family constellations, spanning harmonious networks to more conflictual relationships. Religious or spiritual beliefs emerge as significant, serving either as a source of strength through practices like prayer or ritual, or as a point of tension when traditions evolve. Cultural expectations around caregiving, such as the notion that care should remain ‘within the family,’ are also explored, revealing both the burdens and benefits associated with these traditions.

#### Plot

The plots are structured with a beginning, middle, and end, ensuring coherence and engagement throughout the narrative. Core themes identified during research and the needs assessment guide the progression of the story, with conflicts and turning points thoughtfully integrated to create a meaningful and logical flow.

#### Setting

Realistic and vivid descriptions of the settings create an immersive atmosphere. Whether focusing on domestic environments, community centers, or everyday scenarios, the settings provide a tangible context for the stories, allowing readers or listeners to better understand and connect with the stories.

#### Dramaturgy

Dramatic moments, biographical breaks, and conflicts are authentically depicted to emotionally engage the audience. These moments serve as key drivers of the plot, creating turning points that capture attention and deepen understanding of the challenges and dynamics of caregiving.

#### Narrator

The first-person perspective is used to foster a deeper identification with the narrating protagonist, as it allows readers to directly experience their thoughts, emotions, and perceptions. This creates a personal connection and enables readers to witness events firsthand.

#### Language style

The language is simple, appealing, and inclusive, creating an accessible story for a diverse audience with different educational backgrounds. The tone balances emotional depth without veering into clichés or excessive dramatization, ensuring that the stories remain authentic and respectful.

By combining these elements thoughtfully, the stories not only engage readers or listeners but also achieve their goal of fostering empathy, understanding, and empowerment for caregivers.

### Quality assessment

The objective of developing the stories was to ensure that they were sensitive to diversity and intersectionality, thereby facilitating identification with them by family caregivers and strengthening their self-management skills. The quality assessment of the developed stories was a key step in ensuring their effectiveness and relevance to the target audience [25, 26]. This process focused on evaluating the stories against a set of well-defined criteria [31] designed to guarantee their clarity, accessibility, and suitability for the intended purpose. Additionally, the quality assessment of the stories was based on a pretest with family caregivers of Turkish individuals living with dementia according to the Community-based Participatory Research (CBPR) approach [32].

The criteria were derived from the elements of a ‘good story’ according to Holzinger and Sturmer [31], adapted and revised by an ‘interpretation group’ within the project team. These criteria are structure, language, length, relatability, problem orientation, open ending, validity, and adaptability.

The **structure** of the stories should follow a standardized three-phase approach. First, an Introductory Phase introduces the protagonists and the initial context. This is followed by the Problem Description Phase, where the cause and nature of the problem are outlined. Finally, the Closing Phase describes the consequences of the problem, such as feelings of fear or helplessness.

The **language** of the stories should be simple and accessible, ensuring that participants can easily engage with the content. Given the diverse backgrounds of the individuals involved, who may not all share the same level of education, it is crucial to use clear and straightforward wording.

The **length** of the stories should be concise, summarizing essential details while maintaining brevity. A reading time of approximately five minutes is recommended to keep participants focused and ensure that all information is absorbed without overwhelming them.

To enhance **relatability**, the realism of the stories is vital. The content should closely mirror the everyday experiences of family caregivers and individuals in need of care. True-to-life stories enable participants to connect with the material more effectively and actively engage in discussions.

**Problem orientation** is another essential criterion. Each story should address a problem relevant to the participants’ lives. This provides a foundation for subsequent discussions, where potential solutions can be explored collectively.

Including an **open ending** in the stories facilitates greater engagement from participants. Those with prior experience of the depicted problems are encouraged to share their own stories, while those unfamiliar with such issues are prompted to think critically and explore the topics further.

**Validity** is a cornerstone of quality storytelling. The stories should meet high qualitative standards, particularly communicative validity (as defined by Mayring [33]). This means that participants should interpret the stories as intended by the storytellers. To ensure this, pretests can be conducted where family caregivers listen to the stories and are asked to recount their understanding of the content.

Lastly, **adaptability** is crucial. The topics of the stories should align with the specific needs of the participants, which are identified in advance. Tailoring the stories to these needs ensures they are relevant and meaningful to the target audience.

To ensure the quality and relevance of the stories, pretests were conducted with three family caregivers of Turkish individuals living with dementia as part of a CBPR approach [23, 34, 35]. A purposive sampling approach with a maximum contrasting comparison, where individuals are deliberately selected for their relevant information concerning the research question, and who differ in key characteristics (e.g., age, role in care, relationship to patient), was used to collect a broad range of information [36] (Table 2). Participants for the pretest were recruited via social media, specifically Facebook groups for Turkish individuals living with dementia and their families, to obtain initial formative feedback on the storytelling packs. The purpose was to gather qualitative insights to refine language, narrative structure, and cultural sensitivity.

**Table 2:** Self-reported sample description of the participants of the pretest

Age range	Role in Care	Relationship to Patient	Employment	Family Situation	Cultural & Traditional Expectations	Use of Professional Support
45–54	Primary caregiver	Daughter-in-law	Part-time	Married with children	Very strong	Minimal
30–39	Supporting caregiver	Granddaughter	Full-time	Married without children	Moderate	Utilizes support services
18–24	Emergency caregiver	Granddaughter	In education	Not married without children	None	Prefers full professional care

Using a think-aloud approach according to Charters [37], the draft version of the story was read aloud to the participants. Following the reading, the participants systematically reviewed the story using the defined quality criteria. The verbal statements of the participants were documented to ensure an accurate representation of their thought processes. The data were analyzed inductively using qualitative content analysis according to Mayring [33], allowing themes and patterns to emerge directly from the data. This approach enabled a comprehensive evaluation of whether the developed stories met the predetermined criteria. The pretests provided invaluable insights into how well the stories aligned with the needs and experiences of the participants, ensuring the material was both relatable and impactful [38, 39].

The pretests provided valuable feedback, helping to assess whether the story met these standards and resonated with the experiences and expectations of the caregivers. Insights gained during this process guided further refinement of the story to ensure its practical application and life-world relevance. By applying these criteria, the assessment aimed to create stories that were not only informative but also engaging and adaptable to real-world contexts. The focus was on producing high-quality narratives that could effectively facilitate understanding, stimulate discussion, and address the challenges faced by the target group in a meaningful way.

## Results

The results of the three-stage development process of the stories, focusing on the key findings from the needs assessment and the development and quality assessment, will be presented below. The needs assessment based on a literature review and qualitative secondary data analysis revealed the complex and varied experiences of family caregivers of Turkish individuals living with dementia, emphasizing the importance of recognizing diversity in caregiving roles. Building on these findings, a series of diversity sensitive and intersectional stories were developed according to the principles of a ‘good story’ to address the specific needs of the caregivers. The stories were further evaluated against a set of well-defined criteria and pretests with the target group, ensuring that they were both relevant and engaging.

### Caregivers’ perspectives and needs

The literature review and qualitative secondary data analysis revealed that distinguishing between individual and structural challenges is essential for understanding the experiences of family caregivers. It became clear that the role of the caregiver must be examined with particular attention, as caregivers’ needs and circumstances can vary widely depending on their relationship with the person living with dementia – whether as a spouse, parent, sibling, or child [40]. Additionally, factors such as gender, age, socio-economic status, education, and broader family context play a significant role in shaping these needs [41]. A diversity-sensitive approach must therefore account for these variations. From an intersectional perspective, it is also important to recognise that caregivers differ not only in demographic characteristics but also in terms of their identities, structural positions, and lived representations [27]. The qualitative secondary data analysis underscored that these diverse dimensions lead to significant differences in caregiver experiences, making it inappropriate to treat them as a homogeneous group. Instead, the data point to distinct stressors and coping strategies associated with different caregiving roles, each requiring tailored support approaches. The needs assessment thus identified several caregiver profiles, each characterised by specific diversity attributes and caregiving roles. Addressing these distinctions is crucial for strengthening caregivers’ self-management capacities and for informing the development of stories that are both relevant and inclusive. The following section outlines the key caregiver types that form the foundation for the story development process.

1. Caregiver who is under multiple pressures, juggling the demands of work with caring for the family member living with dementia and bringing up children.
2. Caregiver who feels lonely, overwhelmed and burdened by family conflicts and the fate of her family member’s illness.
3. Caregiver who is heavily burdened, suffering from differing views about dementia and how to deal with it within the family, unable to organise care as they would like.
4. Caregiver who accepts their role and puts their own needs and well-being second out of love and in order to meet the expectations of others and maintain social peace.
5. Caregiver who provides care due to favourable circumstances, traditional/cultural norms, family expectations and hierarchical structures.
6. Caregiver who is a sole, active, controlling and decisive caregiver, using professional support because they believe that other family members are not competent to do so.
7. Caregiver who makes care decisions at their own discretion, in line with their own life, also making use of professional support.
8. Caregiver who actively disengages from the caregiving situation, playing a maximally supportive, rather observing role.
9. Caregiver who is informed about dementia and care and therefore trusts only professionals and sees himself as a committed knowledge broker.

The classification of the nine caregiver personas was developed through a combined analysis of secondary qualitative data and existing literature on caregiving diversity and roles. Caregiving experiences are known to be highly heterogeneous, shaped by factors such as socio-economic status, cultural background, gender, family dynamics, and the specific care context [41, 42]. Prior research highlights that caregivers adopt various roles and face distinct challenges depending on their relationship to the care recipient, their personal resources, and cultural expectations [43, 44]. The personas reflect these complex dimensions by integrating empirical insights with theoretical frameworks on caregiving diversity, ensuring that the classifications capture the multifaceted nature of family caregiving within the Turkish migrant context.

Caregiving roles are shaped by diverse factors, requiring tailored stories to reflect these diverse realities authentically. Therefore, diversity-sensitive and intersectional stories for family caregivers are essential to address their varied and complex needs. The heterogeneity of the target group’s care requirements gives rise to divergent interests among family caregivers. While some family carergivers prioritise emotional topics such as the division of care within the family and conflicts, others focus on practical concerns like finances and formalities or the intricacies of the German healthcare system [11, 45]. To ensure a comprehensive understanding of the diversity of experiences and intersecting needs, it was imperative to explore a broad spectrum of topics, thereby ensuring no aspect was overlooked. Special attention was given to communication intensive, sensitive and often stigmatised issues.

However, there is a paucity of research conducted on the subject of enhancing self-management skills among Turkish family caregivers in Germany. A seminal study by Tezcan-Güntekin and Razum [29] analysed the factors that inhibit or promote the strengthening of self-management skills, and identified intersectionalities in the activation and strengthening of self-management skills. As part of an individualised intervention for family caregivers of Turkish individuals living with dementia (‘FörGes 5’) [30], existing self-management skills were examined through qualitative interviews to gain insight into health literacy and empowerment in caregiving. The findings revealed that the caregivers exhibited a wide range of self-management competencies, which were influenced by both existing supportive resources and diverse, intrafamily-specific psychosocial and life-world challenges in caregiving that had not been addressed. The development of self-management skills in family caregivers is determined by a complex interplay of both available resources that support these skills and challenging caregiving situations. This dynamic interplay gives rise to a range of levels of self-management ability among family caregivers [11].

### Development of the stories

Based on the needs assessment, elements of a ‘good story’, and quality assessment, a total of nine story packs were developed to support family caregivers of individuals living with dementia. Each of the story packs contains an introductory story and four follow-up stories. Nine introductory stories have been created to correspond to different types of family caregivers and their needs. These introductory stories serve as a starting point for the caregivers and deal with a specific protagonist tailored to their own diversity characteristics. The additional four stories address the key topics that were identified as part of the needs assessment. Consequently, each story pack contains five stories for a specific type of caregiver. With nine caregiving types, a total of 45 stories were developed.

All stories were developed in German and then translated into Turkish by native speakers from the project team to further increase cultural and linguistic accessibility for the target group. For the purposes of this article, the stories were also translated into English. The stories include cross-cultural protagonists in similar life situations as the type of caregivers represented. The protagonists and content take into account identified diversity characteristics such as caring roles, family support, religious beliefs, cultural traditions and other relevant dimensions. All nine story packs developed can be viewed and downloaded in English, German and Turkish in the Supplemental Files 1–3.

#### Introductory story

**Fig. 2:**
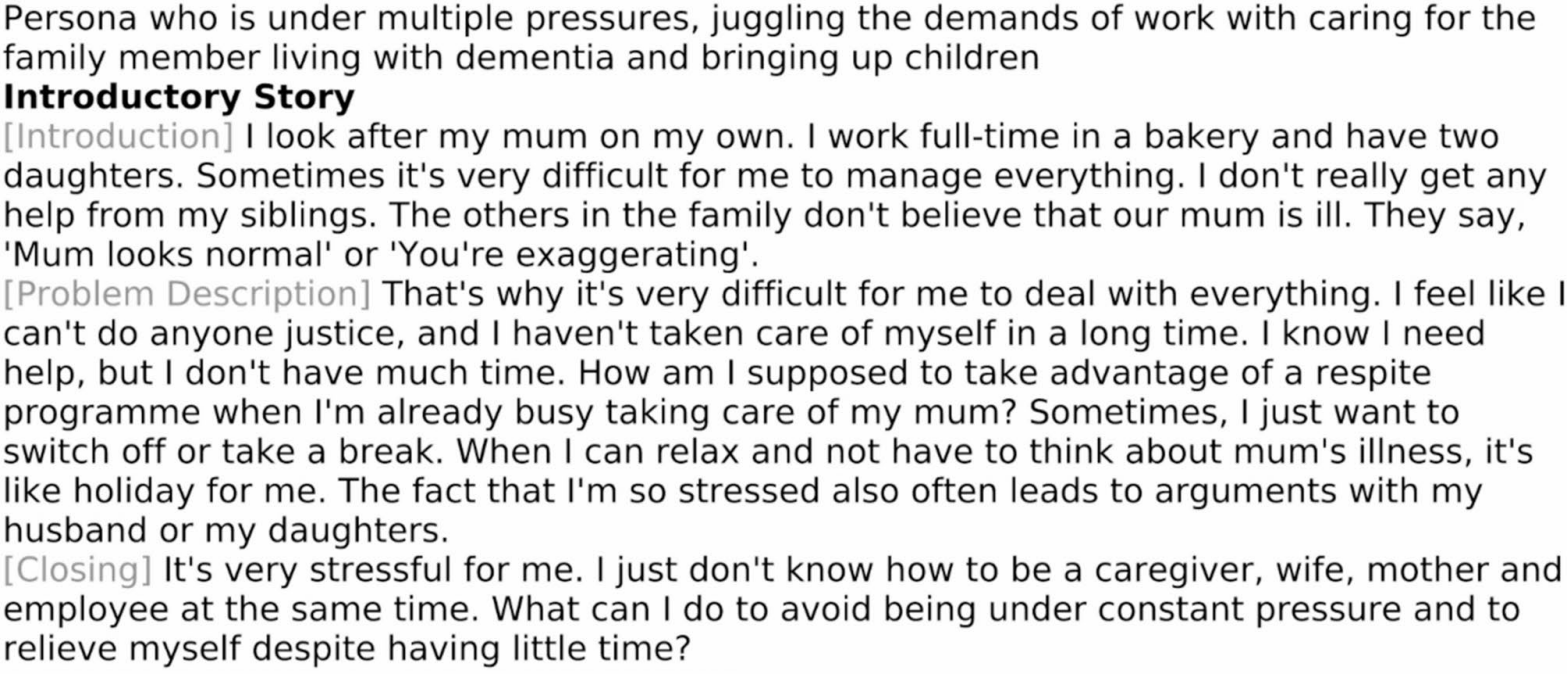
Introductory Story for Persona 1

The nine introductory stories serve as entry points for readers and listeners, each carefully tailored to reflect different caregiver types identified in the needs assessment. They portray relatable, everyday life scenarios and challenges, enabling the audience to connect emotionally and cognitively with the story’s protagonist. These stories are designed to align with a diverse range of caregiver characteristics, fostering identification with the protagonists and encouraging continued engagement with the subsequent storylines. Each introductory story centers on a distinct persona, reflecting the diversity of family caregiving roles outlined in the needs assessment. Figure 2 provides an example of an introductory story featuring Persona 1.

#### Follow-up stories

**Fig. 3:**
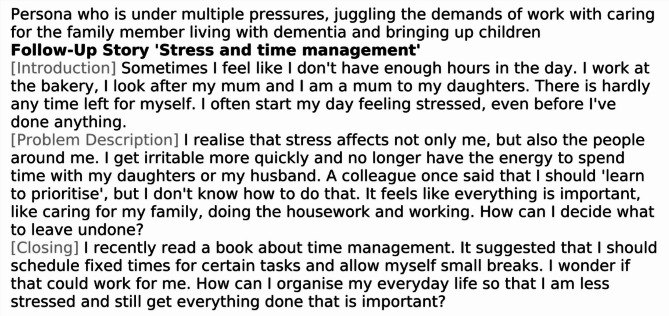
Example follow-up story for Persona 1 with the key topic ‘Stress and time management’

**Fig. 4:**
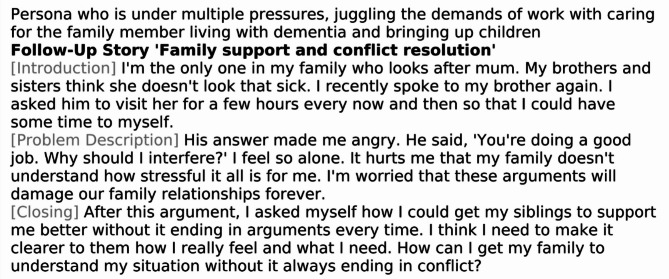
Example follow-up story for Persona 1 with the key topic ‘Family support and conflict resolution’

The follow-up stories are thematically aligned with the corresponding introductory stories in each narrative set and are designed to explore and elaborate on specific issues relevant to each persona. Each caregiver persona is characterized by unique strengths, needs, and ways of shaping their caregiving and living environments. As such, different themes emerge as particularly relevant depending on the individual’s circumstances. Some topics require greater emphasis, while others may be less pertinent. To ensure the stories reflect this diversity-sensitive approach, key thematic areas were identified for each persona based on the findings of the needs assessment. These findings were systematically reviewed and discussed during several project team meetings involving both researchers and practitioners. Table 3 presents the key topics addressed in the follow-up stories for each persona. These stories offer deeper insight into everyday caregiving challenges and provide content and practical guidance for further discussion. Figures 3 and 4 illustrate example follow-up stories for Persona 1, focusing on the topics of ‘Stress and time management’ and ‘Family support and conflict resolution’, respectively.

**Table 3:** Key topics of the follow-up stories for each persona in each story pack

Persona	Key topics of the follow-up stories			
1	Stress and time management	Family support and conflict resolution	Professional support services	Balancing different roles
2	Dealing with loneliness	Overload in care	Religious/spiritual support and inner strength	Loss of identity
3	Different views in the family	Dealing with family conflicts	Overcoming the stigma of dementia	Dealing with powerlessness
4	Loss of identity	Acceptance of support	Emotional burden of self-sacrifice	Dealing with own needs
5	Caregiving out of family duty	Social and cultural expectations	Support and responsibility	Self-fulfilment and independent living
6	Lack of trust in other family members	Control and overload in care	Need for perfection	Professional support
7	Care and organisation	Making decisions	Professional support	Finding emotional balance
8	Support in care	Distinction from care	Family support	Role in care
9	Trust in experts	Control and overload in care	Social and cultural expectations	Knowledge and support

### Quality assessment

The pretests conducted as part of the CBPR approach with three family caregivers of Turkish individuals living with dementia provided valuable insights into the quality and relevance of the stories. The feedback collected during these pretests helped assess whether the stories met the established standards and resonated with the caregivers’ experiences and expectations. The caregivers expressed satisfaction with the overall themes of the stories and the protagonists, indicating that no major changes to the content were necessary. However, they suggested small modifications with regard to the ‘language style’ criteria, specifically in wording and phrasing, to enhance clarity and emotional resonance.

Based on this feedback, the stories were refined and adjusted accordingly. The revisions focused on fine-tuning the language to ensure that the narratives were more engaging and easier to relate to, while maintaining their original content and intent. After these minor adjustments, the stories were finalized by the interdisciplinary project team, which includes experts in dementia care, caregiving, diversity sensitivity, and migration and health. The development and finalization process was guided by theoretical frameworks including intersectionality, the Health Belief Model, and storytelling. This ensured that the story packs not only reflected real-life experiences but also addressed the complex and diverse needs of Turkish family caregivers of individuals living with dementia. The pretest process ultimately ensured that the stories were not only informative but also aligned with the caregivers’ real-life experiences, making them more effective in addressing the challenges faced by the target group.

## Discussion

The development of diversity-sensitive and intersectional stories for family caregivers is highly relevant, as these narratives have the potential to address the complex and multifaceted needs of caregivers in a meaningful and nuanced way. Employing storytelling as a methodological foundation enables the creation of care-related, open-ended narratives that resonate with real-life experiences. This approach is particularly well-suited to capturing both the emotional and practical challenges commonly faced by caregivers [24]. Storytelling fosters empathy and connection by highlighting shared experiences, allowing caregivers to see themselves reflected in the stories and to draw strength and insight from them [23]. Despite the clear demand for such approaches, there remains a notable gap in the availability of culturally appropriate stories within the storytelling framework that specifically address the care and living circumstances of caregivers of Turkish individuals living with dementia. To bridge this gap, we developed nine story packs that authentically reflect the unique challenges and lived experiences of this target group, with the aim of enhancing the quality of care they provide. The open-ended nature of these stories allows for multiple perspectives and interpretations, making them particularly valuable in diverse caregiving contexts. Grounded in real-life situations, the stories offer points of identification and may help alleviate the emotional burden placed on caregivers. Preliminary findings from the development phase indicate that storytelling shows considerable promise as a tool for promoting self-help and emotional resilience among family caregivers.

The stories developed contribute to the existing literature on approaches to support for caregivers with multiple diversity characteristics. Caregiving roles are deeply influenced by a range of factors, including cultural background, gender, age, socio-economic status, educational level, and family dynamics, all of which shape the caregiving experience and the associated challenges [40, 41, 46]. Recognizing these intersectional dimensions ensures that stories resonate authentically with the diverse realities of caregivers, fostering a sense of representation and empowerment and facilitating greater identification and a sense of belonging. The findings of this study are consistent with those of previous research and contribute to the existing body of knowledge, particularly in relation to the importance of inclusion, participation, and emotional support for family caregivers [47]. The consideration of diversity characteristics, such as migration history, gender, socio-economic status and cultural influences is in line with the current discourse on intersectional approaches in care and health research [15, 17]. The intersectional perspective allows for a more in-depth examination of the complex challenges that arise from the confluence of multiple diversity characteristics, thereby taking into account the multifaceted realities of family caregivers. This approach has already been identified in the literature as a necessary step to reduce discrimination and inequalities in care [16]. While existing literature focuses primarily on narrative approaches and self-management [47], the created stories extend the discussion to include the need to consider other diversity characteristics such as religious affiliation, cultural traditions and social networks. This has been less emphasised in previous studies, highlighting the practical relevance and necessity of these additional dimensions. Furthermore, the study highlights the importance of linguistic and cultural accessibility in the context of narrative-based discourse on inclusive health approaches [7, 48, 49].

In addition to fostering representation and emotional validation, the stories can actively contribute to supporting caregivers’ emotional recovery and empowerment. By offering diverse role models and portraying various coping strategies, the stories enable caregivers to reflect on their own situation and draw strength from shared experiences. The open-ended structure also invites discussion and peer exchange, which can enhance mutual support and collective learning. Moreover, the stories incorporate themes of resilience, boundary-setting, and self-care, thereby promoting emotional self-help and improved well-being. As such, the story packs not only raise awareness about caregiving challenges but also serve as a practical tool for initiating dialogue and reflection, both individually and in group settings. This contributes to strengthening self-management and may ultimately enhance the quality of care provided to people with dementia.

The strengths of the methodical multi-stage approach used for the development of the stories in the present study lie in the adaptive and inclusive design of the stories, which are oriented towards the individual and often complex needs of family caregivers. The needs assessment served as a critical foundation for this process, as it illuminated the intricate interplay between structural and individual challenges faced by caregivers [13, 50]. The assessment uncovered variations in self-management abilities and caregiving experiences that are shaped by both supportive resources and unique psychosocial stressors. These variations in different factors resulted in nine different caring roles that could be identified. Further literature review showed that it is vital to take these roles into account when developing support services. This understanding underscores the importance of tailoring stories to reflect the specific needs, identities, and coping strategies of different caregiving roles [40, 41, 45]. Without the insights gained through the needs assessment, the development of such stories would risk oversimplifying the caregiving experience or neglecting the diversity of the target audience. Instead, the assessment provided a nuanced understanding of caregivers’ lived realities, enabling the creation of stories that are not only inclusive but also capable of addressing key barriers to self-management [51]. The combination of narrative and intersectional approaches encourages deeper engagement with caring issues and facilitates caregivers’ personal development. In addition, the cultural and linguistic accessibility of the stories facilitates wider participation of the target audience.

### Limitations

However, the complexity of diversity characteristics also entails potential limitations. The interpretation of the results must consider the specific focus of this study — family caregivers of Turkish individuals living with dementia in Germany — which may limit the generalizability of the findings to other cultural groups or caregiving contexts. Furthermore, the secondary data analysis is based on only three studies, which may restrict the breadth of the needs assessment. Nevertheless, these studies were conducted in diverse contexts, offering a certain degree of variation. Together, they include 31 qualitative interviews that were analyzed through a multi-stage intersectional multilevel approach, providing a comprehensive and nuanced needs assessment despite the limited number of studies. Additionally, while the developed stories underwent a multi-step quality assessment to enhance their authenticity and efficacy, the pretest phase involved only three family caregivers. Although the pretest involved only three participants, the process was conducted in alignment with the key principles of CBPR. This formative application aimed to actively engage family caregivers in providing feedback and shaping the development of the storytelling materials. We acknowledge that this small sample does not represent a full CBPR study but serves as an initial participatory step to ensure relevance and cultural sensitivity. Future research could benefit from involving a larger and more diverse group of participants to further validate and refine the storytelling approach.

Furthermore, it should be noted that the practical applications of the stories are subject to certain limitations. The need to consider a variety of different characteristics could hinder the development process and affect the speed of adaptation to new needs. Additionally, the implementation of such sensitive stories requires regular training and education of facilitators to prevent discrimination and ensure the support of all participants. Although these stories are thoughtfully designed to be adaptable, inclusive, and impactful, further evaluation studies are necessary to assess their effectiveness. Such studies are essential to understand how well the stories achieve their intended goals, such as raising awareness, promoting empathy, or driving behavioral change. Evaluation can provide valuable insights into the stories’ impact across different settings, populations, and contexts, ensuring they are not only theoretically sound but also practically effective. This evidence-based approach will help refine the stories and maximize their potential benefits.

It is essential that these stories are intersectional and sensitive to diversity, thoughtfully representing a wide range of individuals, experiences and roles. Rather than reinforcing stereotypes, the stories aim to challenge and dismantle them, fostering a more inclusive and nuanced understanding of diverse perspectives. The developed stories are highly versatile and can be used in various settings. They are adaptable to different population groups, patient demographics, illnesses, and life contexts, ensuring their long-term relevance. Storytelling has broad applications across many fields, making it a powerful tool for communication and education. Additionally, in aged, palliative, and end-of-life care contexts, stories can help raise awareness for the lived experiences of caregivers and care recipients alike. To ensure sustainable implementation, storytelling formats should be designed to complement existing workflows, potentially involving trained facilitators, volunteers, or digital tools, without adding to the burden of healthcare professionals.

### Conclusions

The development of stories has significant implications for both practice and further research. In practice, the stories could be established as a supportive tool in self-help groups for family caregivers, with the aim of reducing their psychological distress and strengthening self-management. In addition, the stories produced could be used in the training of professional caregivers and in the provision of counselling and support services by social services.

In terms of research, the narrative and intersectional methodology offers the potential for further studies focusing on the specific needs and challenges of caregivers with different diversity characteristics. Further development of narrative approaches could facilitate a more comprehensive examination of both the quantitative and qualitative aspects of caregivers’ self-care and emotional wellbeing, thereby strengthening the evidence base for their effectiveness. Future research should explore how storytelling can be systematically embedded in care practices to promote equity and well-being. This study highlights the importance of integrating intersectionality and narrative-based approaches, such as storytelling, into healthcare to address the multiple roles and challenges faced by family caregivers in the Turkish community living in Germany. Storytelling fosters empathy, cultural understanding, and deeper engagement with caregivers’ lived experiences, enabling more inclusive and personalised care. Particularly for vulnerable groups such as family caregivers, storytelling has the potential to serve as a valuable tool to reduce stress and improve self-management skills, thereby strengthening their resilience and ability to provide care in a sustainable way.

## Supplementary Information

Below is the link to the electronic supplementary material.

Supplementary Material 1

Supplementary Material 2

Supplementary Material 3

## Data Availability

The story packs developed are available in English, German and Turkish in the Supplemental Files 1-3.
