# Supplementary material for: Storytelling in family-provided care - developing diversity-sensitive stories for family caregivers of Turkish individuals living with dementia
Source: BMC Nurs. 2025 Aug 12;24:1060. doi: 10.1186/s12912-025-03712-7 (PMC12341143; doi:10.1186/s12912-025-03712-7)
Supplement: Supplementary file 1 — Supplementary Material 1 [file 12912_2025_3712_MOESM1_ESM.pdf]

Persona 1: Persona who is under multiple pressures, juggling the demands of work with caring for the family member living with dementia and bringing up children

Overview Story Pack

Story	Thema
Story 1.1	Introduction
Story 1.2	Stress and time management
Story 1.3	Family support and conflict resolution
Story 1.4	Professional support services
Story 1.5	Balancing different roles

Persona 1: Persona who is under multiple pressures, juggling the demands of work with caring for the family member living with dementia and bringing up children

Story 1.1: Introduction

[Introduction] I look after my mum on my own. I work full-time in a bakery and have two daughters. Sometimes it's very difficult for me to manage everything. I don't really get any help from my siblings. The others in the family don't believe that our mum is ill. They say, 'Mum looks normal' or 'You're exaggerating'.

[Problem Description] That's why it's very difficult for me to deal with everything. I feel like I can't do anyone justice, and I haven't taken care of myself in a long time. I know I need help, but I don't have much time. How am I supposed to take advantage of a respite programme when I'm already busy taking care of my mum? Sometimes, I just want to switch off or take a break. When I can relax and not have to think about mum's illness, it's like holiday for me. The fact that I'm so stressed also often leads to arguments with my husband or my daughters. It's very stressful for me.

[Closing] I just don't know how to be a caregiver, wife, mother and employee at the same time. What can I do to avoid being under constant pressure and to relieve myself despite having little time?

Persona 1: Persona who is under multiple pressures, juggling the demands of work with caring for the family member living with dementia and bringing up children

Story 1.2: Stress and time management

[Introduction] Sometimes I feel like I don't have enough hours in the day. I work at the bakery, I look after my mum and I am a mum to my daughters. There is hardly any time left for myself. I often start my day feeling stressed, even before I've done anything.

[Problem Description] I realise that stress affects not only me, but also the people around me. I get irritable more quickly and no longer have the energy to spend time with my daughters or my husband. A colleague once said that I should 'learn to prioritise', but I don't know how to do that. It feels like everything is important, like caring for my family, doing the housework and working. How can I decide what to leave undone?

[Closing] I recently read a book about time management. It suggested that I should schedule fixed times for certain tasks and allow myself small breaks. I wonder if that could work for me. How can I organise my everyday life so that I am less stressed and still get everything done that is important?

Persona 1: Persona who is under multiple pressures, juggling the demands of work with caring for the family member living with dementia and bringing up children

Story 1.3: Family support and conflict resolution

[Introduction] I'm the only one in my family who looks after mum. My brothers and sisters think she doesn't look that sick. I recently spoke to my brother again. I asked him to visit her for a few hours every now and then so that I could have some time to myself.

[Problem Description] His answer made me angry. He said, 'You're doing a good job. Why should I interfere?' I feel so alone. It hurts me that my family doesn't understand how stressful it all is for me. I'm worried that these arguments will damage our family relationships forever.

[Closing] After this argument, I asked myself how I could get my siblings to support me better without it ending in arguments every time. I think I need to make it clearer to them how I really feel and what I need. How can I get my family to understand my situation without it always ending in conflict?

Persona 1: Persona who is under multiple pressures, juggling the demands of work with caring for the family member living with dementia and bringing up children

Story 1.4: Professional support services

[Introduction] Last week I spoke to a colleague at work who told me about a care service in our neighbourhood. She said that she had found support for her mother-in-law there. I thought about whether that would be something for us, but I don't know if I could integrate it into my everyday life.

[Problem Description] I really need some relief, but I have the feeling that I don't have the time. Caring for mum is hard enough as it is, and now organising an appointment with a care service seems impossible. I also have doubts as to whether the carer is really right for us. I've heard that many carers don't speak Turkish. Mum barely understands German, so how is she supposed to communicate with them? I'm also worried that they won't take things into account that are important to mum - for example, that she doesn't eat pork. Mum has also told me that she doesn't want ‘strangers’ in the house. I don't know how to explain to her that we need this support.

[Conclusion] I wonder whether such a care service could really help and how I can convince mum to at least give it a try. Maybe I should ask beforehand if the carers can take things like language and diet into consideration or if there are even Turkish carers. How do I find a care service that understands our needs and how can I convince my mum to give it a try?

Persona 1: Persona who is under multiple pressures, juggling the demands of work with caring for the family member living with dementia and bringing up children

Story 1.5: Balancing different roles

[Introduction] Sometimes I wonder how I manage to fulfil all my roles. I am a caregiver, mother, wife, employee - but where does that leave me? Recently, my daughter asked me why I so rarely have time for her. It hit me hard to hear that.

[Problem Description] I realise that I am constantly trying to fulfil all expectations, but in the process I lose myself. When I spend more time with my daughters, the caring falls by the wayside. When I focus on caring, my relationship with my husband suffers. It feels like I can't please anyone.

[Closing] I've started to think about what I really want and what I should prioritise. Maybe I need to learn to communicate my boundaries more clearly and ask for more support, but I find that difficult. How can I manage to balance my different roles better and still find time for myself?

Persona 2: Persona who feels lonely, overwhelmed and burdened by family conflicts and the fate of her family member's illness

Overview Story Pack

**Story**	**Thema**
Story 2.1	Introduction
Story 2.2	Dealing with loneliness
Story 2.3	Overload in care
Story 2.4	Religious/spiritual support and inner strength
Story 2.5	Loss of identity

Persona 2: Persona who feels lonely, overwhelmed and burdened by family conflicts and the fate of her family member's illness

Story 2.1: Introduction

[Introduction] I have been taking care of my father alone since my mother passed away three years ago. There is no one who could help me, and I see caregiving as my duty before God. My father gave me life, and now it is my responsibility to be there for him. However, I increasingly feel the heavy weight of this responsibility on my shoulders.

[Problem Description] I no longer have real contact with people outside my family, and my days follow the same routine. I wake up, take care of my father, manage the household, and handle everything that needs to be done. There is no time left for myself, and sometimes I wonder who I am besides being a caregiver. I pray every day for the strength to carry on, but often, I feel overwhelmed and alone.

[Closing] Sometimes, I wish I had someone to talk to—someone who understands how hard it is to bear everything alone. But I have learned to keep my feelings to myself because it is not proper to complain. How can I find relief in my situation without neglecting my responsibilities or my values?

Persona 2: Persona who feels lonely, overwhelmed and burdened by family conflicts and the fate of her family member's illness

Story 2.2: Dealing with loneliness

[Introduction] Sometimes, I notice how quiet it is around me. My father hardly speaks anymore, and I have no one to talk to. I used to have friends, but I lost touch with them because I am always at home.

[Problem Description] I know that loneliness is not good for me, but I feel powerless to change it. Sometimes, I dream of simply talking to someone or even meeting someone who is going through a similar situation. But I wouldn’t even know how to do that without neglecting my caregiving responsibilities.

[Closing] I wonder if there are ways to reconnect with others despite my situation. Maybe it would help me feel less alone. How can I overcome my loneliness without neglecting my duties?

Persona 2: Persona who feels lonely, overwhelmed and burdened by family conflicts and the fate of her family member's illness

Story 2.3: Overload in care

[Introduction] The other night, my father called for me because he was in pain. I helped him, but after that, I couldn't fall back asleep. Nights like these are becoming more frequent, and I can feel my energy slipping away.

[Problem Description] Caregiving is becoming more exhausting, and I often don’t know how to handle it. There are moments when I feel completely overwhelmed. I worry that I’m not doing enough, even though I’m already giving everything I have.

[Closing] Sometimes, I wonder if there is someone who could show me how to manage caregiving better without breaking myself in the process. But I don’t know anyone, and I wouldn’t even know how to do things differently. How can I cope with feeling overwhelmed while still ensuring my father gets the care he needs?

Persona 2: Persona who feels lonely, overwhelmed and burdened by family conflicts and the fate of her family member's illness

Story 2.4: Religious/spiritual support and inner strength

[Introduction] I find comfort in my faith. Every day, I pray that Allah gives me the strength to care for my father. I see caregiving as my duty and also as a way to live my faith.

[Problem Description] Despite my faith, I sometimes have doubts. There are days when I wonder if I am doing enough or if I am truly meeting my father's needs. These thoughts stay with me, and I feel guilty when I realize that my strength is fading.

[Closing] I wonder how I can rely even more on my faith to overcome the challenges of caregiving. Perhaps there are ways to fulfill my duties with greater inner peace. How can my faith help me find strength in difficult moments?

Persona 2: Persona who feels lonely, overwhelmed and burdened by family conflicts and the fate of her family member's illness

Story 2.5: Loss of identity

[Introduction] Since I started caring for my father, my life has completely changed—though not intentionally. I used to have hobbies and dreams, but now everything revolves around caregiving. I have no time for friends and no desire to try new things. I no longer know who I am outside of this role.

[Problem Description] It feels like I have lost myself. I miss the person I once was, but I don’t know how to find my way back without neglecting my duty. Sometimes, I fear that I will never have time for myself again.

[Closing] I wonder if it’s possible to regain some part of my old life without giving up my role as a caregiver. Maybe there is a way to balance both. How can I reconnect with myself while still being there for my father?

Persona 3: Persona who is heavily burdened, suffering from differing views about dementia and how to deal with it within the family, unable to organise care as they would like

Overview Story Pack

Story	Thema
Story 3.1	Introduction
Story 3.2	Different views in the family
Story 3.3	Dealing with family conflicts
Story 3.4	Overcoming the stigma of dementia
Story 3.5	Dealing with powerlessness

Persona 3: Persona who is heavily burdened, suffering from differing views about dementia and how to deal with it within the family, unable to organise care as they would like

Story 3.1: Introduction

[Introduction] I support my father in taking care of my mother, who suffers from dementia. Although I am not the primary caregiver, I try to help as much as I can. Unfortunately, there are constant conflicts in our family about how we should handle Mom's illness.

[Problem Description] My father believes that Mom just needs more attention and that she could "pull herself together." My sister, on the other hand, thinks we should leave her more in peace and stop making so much drama. I believe we need professional help, but this suggestion is met with resistance. It feels like no one in the family is really taking me seriously. I know I can't change everything, but I want us to provide Mom with the best care possible – without constantly fighting.

[Conclusion] Sometimes, I feel like I'm caught in the middle of everything. I see my mother suffering, but I can't do anything to shape the care the way I think is right. How can I get my family to work together, and how can I better take on my role in the caregiving?

Persona 3: Persona who is heavily burdened, suffering from differing views about dementia and how to deal with it within the family, unable to organise care as they would like

Story 3.2: Different views in the family

[Introduction] Yesterday, we had another heated discussion about Mom. My father believes we shouldn't give her medication because he thinks it will only make things worse. My sister agrees with him, but I have a different opinion.

[Problem Description] It's so difficult to agree on a common path within the family. I feel like we all want something different and in the process, forget what Mom really needs. I am convinced that we would manage better with medical support, but my suggestions are either overruled or not taken seriously at all. I'm afraid that Mom will get worse if we don't do something about it.

[Conclusion] Sometimes I wonder if I can even make a difference. I want the best for Mom, but how can that be possible if we are constantly disagreeing? How can I make my family understand that we should seek professional advice without leading to more conflicts?

Persona 3: Persona who is heavily burdened, suffering from differing views about dementia and how to deal with it within the family, unable to organise care as they would like

Story 3.3: Dealing with family conflicts

[Introduction] After our last argument, I tried to calmly talk to my sister. I wanted to understand why she was so opposed to my suggestion of seeking caregiving advice. Her answer surprised me: she said she feels overwhelmed and is afraid that we might put Mom in a nursing home.

[Problem Description] I've realized that our conflicts often stem from fears and insecurities. But instead of talking honestly about it, we blame each other for everything we're doing wrong. This weighs heavily on me because I want us to find a solution together – for Mom and for us as a family.

[Conclusion] Maybe we all need to learn how to talk to each other better, without blaming each other. But how do you start such a conversation when everyone is so tense? How can we reduce the family tension and speak more openly about our feelings and fears?

Persona 3: Persona who is heavily burdened, suffering from differing views about dementia and how to deal with it within the family, unable to organise care as they would like

Story 3.4: Overcoming the stigma of dementia

[Introduction] A few days ago, a neighbor asked me why my mother is behaving so strangely. I explained to her that she suffers from dementia, but her reaction really hit me: "That must be so difficult for you – hopefully, people don't notice it too much."

[Problem Description] I feel like many people around us don't understand what dementia means. It's almost as if the illness is a taboo that can't be talked about. My father doesn't want anyone outside the family to know about it, and my sister completely avoids such conversations. But I think we should be open about it in order to break down prejudices and stigma. Even within the family, we find it difficult to talk openly about the disease.

[Conclusion] I wonder how we can find a way to talk about the disease without feeling bad or judged. Maybe that would also help us deal with it better as a family. How can we approach dementia openly while also considering the worries and fears of other family members?

Persona 3: Persona who is heavily burdened, suffering from differing views about dementia and how to deal with it within the family, unable to organise care as they would like

Story 3.5: Dealing with powerlessness

[Introduction] I keep feeling like I can't change anything. No matter how hard I try, others ultimately make the decisions, and I am left with my worries and frustration.

[Problem Description] I find it difficult to accept that I often play a secondary role in caregiving. I see things going wrong, but when I point them out, I'm either ignored or overridden. The feeling of being powerless weighs heavily on me. I wonder if I will ever be able to truly make a difference.

[Conclusion] Sometimes, I think I need to learn to let go – but how can I do that when I know my mother could suffer because of it? How can I deal with my powerlessness and still make a positive contribution to her care?

Persona 4: Persona who accepts the role of caregiver and puts their own needs and well-being second out of love and in order to meet the expectations of others and maintain social peace

Overview Story Pack

Story	Thema
Story 4.1	Introduction
Story 4.2	Loss of identity
Story 4.3	Acceptance of support
Story 4.4	Emotional burden of self-sacrifice
Story 4.5	Dealing with own needs

Persona 4: Persona who accepts the role of caregiver and puts their own needs and well-being second out of love and in order to meet the expectations of others and maintain social peace

Story 4.1: Introduction

[Introduction] I decided to take on the care for my mother after she began needing more and more help in her daily life. She has been living with me for two years now, and I take care of her around the clock. I could never think of putting her in a nursing home – it's just not the right thing for her. In my family, it's a tradition that we are there for each other, especially in difficult times. And as her daughter, it's simply my duty to take care of her.

[Problem Description] Sometimes I wonder how I even manage to keep going. I hardly have time for myself and often feel exhausted. But I don't want to complain – after all, it's my responsibility, and I've taken on this task with love. I feel it would be a sin to do something good for myself when my mother needs my help. But sometimes it just gets too much, and I don't know how long I can keep going like this.

[Closing] I can't stop taking care of her – it would feel wrong. But at the same time, I wonder how long I can maintain this without losing my own health and well-being. How can I handle this constant self-sacrifice without completely losing my own identity and quality of life?

Persona 4: Persona who accepts the role of caregiver and puts their own needs and well-being second out of love and in order to meet the expectations of others and maintain social peace

Story 4.2: Loss of identity

[Introduction] In recent months, I've noticed how much my life has changed. I used to have hobbies, friends, and my own desires and goals. But now, everything revolves around taking care of my mother. It feels as though I've lost myself.

[Problem Description] I often wonder who I am now if I'm not the "caregiver." I take care of Mom, manage the household, and even my sleep is often interrupted because she needs help at night. My friends haven't reached out in a long time, and I've lost touch with everything that used to define me. I'm sacrificing everything I had to be there for her.

[Closing] I feel like I've completely lost myself. I don't know how to find my way back to who I was when I'm constantly living just for her. How can I regain my own identity while still being there for my mother?

Persona 4: Persona who accepts the role of caregiver and puts their own needs and well-being second out of love and in order to meet the expectations of others and maintain social peace

Story 4.3: Acceptance of support

[Introduction] Yesterday, my brother offered to take Mom to the doctor so I could get some rest. I know he means well, but I couldn't accept the offer. It just doesn't feel right to let someone else care for my mom – I can't relax if I do.

[Problem Description] I find it incredibly hard to accept help. I always think I can do it better and that it's my duty to care for her on my own. Sometimes, it even feels embarrassing to ask for help because I fear it will be seen as a weakness. I feel that if I accept help, I would be abandoning my responsibility.

[Closing] I know that taking a break would do me good, but I just can't let go. It feels wrong to put myself first. How can I learn to accept help without feeling like I'm losing my responsibility?

Gefördert durch:
Gemeinsamer Bundesausschuss
Innovationsausschuss

Persona 4: Persona who accepts the role of caregiver and puts their own needs and well-being second out of love and in order to meet the expectations of others and maintain social peace

Story 4.4: Emotional burden of self-sacrifice

[Introduction] I feel like I have less and less of myself left. The emotional burden is immense – I always try to be strong and not let anyone see how exhausted I am. But in the past few weeks, I've realized that I'm completely drained inside.

[Problem Description] It's hard to constantly be there for my mother without ever having a break. I want to give her so much love and care, but at the same time, I feel the weight on my shoulders growing heavier. Sometimes, I cry secretly because I don't know how much longer I can endure this. But I have no one to talk to about it, and I don't want anyone to think I'm failing.

[Closing] I wonder how much longer I can bear this burden. It feels like I'm nothing more than the caregiver – with no room for my own feelings or needs. How can I cope with the emotional load without giving up the care or disappointing my family?

LVF Lehrstuhl für Versorgungsforschung
Universität Witten/Herdecke
ASH Berlin Alice Salomon Hochschule Berlin
University of Applied Sciences
Demenz Support Stuttgart
Zentrum für Informationstransfer

Persona 4: Persona who accepts the role of caregiver and puts their own needs and well-being second out of love and in order to meet the expectations of others and maintain social peace

Story 4.5: Dealing with own needs

[Introduction] It's difficult for me to acknowledge my own needs. I know I sometimes need a break, but I just can't bring myself to take one. It feels selfish to think about myself.

[Problem Description] Every day revolves around my mother's needs – and I feel like there's no time for me. I have no life outside of caregiving, no time for friendships, no time for my own interests. Often, I wonder what it would be like to have just a few hours for myself, but then I immediately realize I can't do that. It feels like my own needs are unimportant. The most important thing is that Mom is okay, and that I can take care of her.

[Closing] I wonder how I can manage to take care of myself now and then, even though I love caring for my mother, without feeling guilty. How can I learn to recognize my own needs and still take on my mother's care with love?

Persona 5: Persona who provides care due to favourable circumstances, traditional/cultural norms, family expectations and hierarchical structures

Overview Story Pack

**Story**	**Thema**
Story 5.1	Introduction
Story 5.2	Caring out of family duty
Story 5.3	Social and cultural expectations
Story 5.4	Support and responsibility
Story 5.5	Self-fulfilment and independent living

Persona 5: Persona who provides care due to favourable circumstances, traditional/cultural norms, family expectations and hierarchical structures

Story 5.1: Introduction

[Introduction] I take care of my mother-in-law, who suffers from dementia. It was never a question whether I would take on this responsibility – it was simply clear that I had to do it. In our family, it is traditional for the younger generations to care for the older ones, especially when they are ill. It is the women who are responsible for this. My husband has no sisters, so it is my duty to take care of her.

[Problem Description] I have never really thought about how I feel about it. It is just the way it has always been done. But the longer I take on the caregiving role, the more I realize that I often have to give up my own desires and needs. There is no time for personal hobbies or friends – everything revolves around caring for my mother-in-law. I feel like I am losing my own identity in the process.

[Closing] It is hard to shake the feeling that I didn't really have a choice but to take on the caregiving. I do it out of duty and love for the family, but sometimes I wonder how much longer I can keep going without neglecting my own desires. How can I find a balance between my responsibility for my mother-in-law and my own needs, without feeling like I am failing to meet the family's and society's expectations?

Persona 5: Persona who provides care due to favourable circumstances, traditional/cultural norms, family expectations and hierarchical structures

Story 5.2: Caring out of family duty

[Introduction] There was never any discussion in the family about me taking on the caregiving role for my mother-in-law. It is simply how things are done in our culture and family. The older generations have always taken care of the younger ones, and now it's my turn to do the same.

[Problem Description] Sometimes I wonder if I made this decision out of my own conviction or simply because it is what family tradition demands. It often feels like there is no choice – if I don't take on the caregiving, it would disappoint the whole family. But deep down, I question whether I am truly happy with this responsibility.

[Closing] There are moments when I wonder if I could focus more on my own life. Yet, the commitment to my family and the cultural norms always hold me back. How can I learn to honor my own desires without violating my family responsibility?

Persona 5: Persona who provides care due to favourable circumstances, traditional/cultural norms, family expectations and hierarchical structures

Story 5.3: Social and cultural expectations

[Introduction] My mother always told me that it was my duty to take care of the family, especially as she gets older. In our culture, it is taken for granted that children will take care of their parents. And in our society, it is seen as a strength to carry this responsibility. I enjoy helping those in need, but I would have liked to have made a conscious choice to do so.

[Problem Description] I feel pressured – both by my family's expectations and societal norms. When I accept help or need a break, I am often indirectly reminded by other family members that it is my duty to be strong and that I can manage. It's a constant challenge to assert my own needs against these expectations.

[Closing] I wonder if I will ever have the right to simply say, "I need a break," and whether I should even do that, because I also want my mother-in-law to be well. How can I get rid of this sense of obligation and allow myself to prioritize my own needs?

Persona 5: Persona who provides care due to favourable circumstances, traditional/cultural norms, family expectations and hierarchical structures

Story 5.4: Support and responsibility

[Introduction] I am the only one who can take care of my mother-in-law – no one else in the family seems really able or willing to support her. I always feel like I have to do everything on my own, which makes it even harder.

[Problem Description] There is little support, even though I feel that it is my duty to take care of everything. My husband and his brothers are either too busy or think that it is not necessary to get involved. I often feel like I have to carry everything on my shoulders.

[Closing] It is a heavy burden that I carry alone, and sometimes I wonder how I can keep managing it when no one else is really helping. How can I learn to accept help without feeling like I am losing control of the responsibility?

Persona 5: Persona who provides care due to favourable circumstances, traditional/cultural norms, family expectations and hierarchical structures

Story 5.5: Self-fulfilment and independent living

[Introduction] The last few years, I've spent solely taking care of my mother-in-law. I used to have plans, wanted to travel and try new things, but none of that has space anymore. All my time goes into caregiving, and I feel like I'm losing myself in the process.

[Problem Description] It's not just the physical strain of caregiving, but also the lack of fulfillment. I wonder what happened to my own dreams and goals. I never get the chance to do something just for myself. I feel like a shadow version of myself – always in the role of the "caregiver."

[Closing] It's hard to accept that my life is now almost entirely defined by caregiving. But the sense of duty to my family leaves me little room to fulfill myself. How can I find ways to live my own life again, without feeling like I'm abandoning my family?

Persona 6: Persona who is a sole, active, controlling and decisive caregiver, using professional support because they believe that other family members are not competent to do so

Overview Story Pack

Story	Thema
Story 6.1	Introduction
Story 6.2	Lack of trust in other family members
Story 6.3	Control and overload in care
Story 6.4	Need for perfection
Story 6.5	Professional support

Persona 6: Persona who is a sole, active, controlling and decisive caregiver, using professional support because they believe that other family members are not competent to do so

Story 6.1: Introduction

[Introduction] I take care of my mother because I believe no one else would do it right. My siblings are either too busy or think it's not a big deal. They don't take the care of my mother as seriously as I do. That's why I decided to take control and handle everything myself. I make sure she is in the best hands, even if it means putting my own needs aside.

[Problem Description] I find it difficult to delegate tasks to other family members. Sometimes I'm not sure they will do it right. That's why I also use professional support. But even then, I keep control because I want to make sure everything runs smoothly and my mother's care is guaranteed.

[Closing] It's exhausting to monitor everything, and sometimes I feel drained. But I just can't stop because I fear that someone else might not take the responsibility as seriously, and my mother could suffer because of it. How can I learn to share responsibility without feeling like I'm losing control?

Persona 6: Persona who is a sole, active, controlling and decisive caregiver, using professional support because they believe that other family members are not competent to do so

Story 6.2: Lack of trust in other family members

[Introduction] My siblings are always ready to help me, but I just can't rely on them. They've never really understood what it means to take care of our mother. And even though they mean well, I know they wouldn't do it as well or as sufficiently as I would.

[Problem Description] I find it hard to give up responsibility. I just can't feel at ease if I don't know exactly what's happening. They might overlook something or do it wrong. These thoughts make me anxious and lead me to do everything myself – even if it overwhelms me. Then I think I should just let go and delegate responsibility.

[Closing] I wonder how long I can keep going like this. Maybe eventually I won't have the strength anymore, but the thought of someone else taking over the care makes me nervous and uncertain. How can I learn to trust others and share responsibility without constantly feeling like I'm losing control?

Persona 6: Persona who is a sole, active, controlling and decisive caregiver, using professional support because they believe that other family members are not competent to do so

Story 6.3: Control and overload in care

[Introduction] It's not easy to handle everything on my own. I know I have to keep control over the care to make sure nothing goes wrong. But at the same time, I feel how much this responsibility is weighing on me. It feels like I always have to be on guard.

[Problem Description] Despite the support of professional caregivers, I feel like I have to be present all the time. I check everything that happens and make sure it goes according to my plans. It's exhausting to maintain this control, but I feel like no one else can do it as well. Yet, the more I try to control everything, the more I realize how it's draining me.

[Closing] It's a constant challenge to find the balance between control and overwhelm. Sometimes I wonder how much longer I can keep this up without it completely wearing me out. How can I learn to give up some control without feeling like my mother's care will suffer because of it?

Persona 6: Persona who is a sole, active, controlling and decisive caregiver, using professional support because they believe that other family members are not competent to do so

Story 6.4: Need for perfection

[Introduction] I make sure everything is in the best condition for my mother. I have hired professional help, but I control everything because I've witnessed them forget things on several occasions. I want my mother to be in the best hands and receive the best support. Therefore, it's difficult for me to cope when someone does something wrong or uses a different method than the one I believe is right.

[Problem Description] I notice that it's becoming increasingly difficult to make everything perfect, especially because I can no longer do everything on my own. I have to delegate some tasks for my mother to be well taken care of. But in doing so, I feel like I'm constantly stressed. I almost feel like a guardian, ensuring that everything stays in perfect order.

[Closing] I wonder if I'll ever be able to give my mother perfect care, or if I'll eventually have to accept that things won't always be perfect. But the thought of letting go scares me because I don't want something to go wrong, and my mother suffers the consequences. How can I learn to control the care less strictly without feeling like it leads to compromises?

Persona 6: Persona who is a sole, active, controlling and decisive caregiver, using professional support because they believe that other family members are not competent to do so

Story 6.5: Professional support

[Introduction] I've hired professional caregivers because I can't handle everything on my own anymore. This was a big step for both me and my mother. To make sure everything goes well, I keep a close eye on the care. I talk a lot with the caregivers and am the direct point of contact if something needs to be discussed.

[Problem Description] I know I need help, but I find it hard to truly accept it. It just doesn't feel right when someone else decides how the care should be provided. I want to make sure they do exactly what I believe is best for my mother. But of course, I also know that the caregivers are the experts in caregiving, and I could give up control – at least a little.

[Closing] It's an ongoing challenge to deal with the help while still feeling like I'm making all the decisions. I wonder if I'll ever really be able to let go and trust the professional caregivers. How can I learn to make use of the support offered to me without feeling unnecessary or controlled?

Persona 7: Persona who makes care decisions rationally and in an organized manner, at their own discretion, in alignment with their own life, with professional support

Overview Story Pack

Story	Thema
Story 7.1	Introduction
Story 7.2	Care and organisation
Story 7.3	Making decisions
Story 7.4	Professional support
Story 7.5	Finding emotional balance

Persona 7: Persona who makes care decisions rationally and in an organized manner, at their own discretion, in alignment with their own life, with professional support

Story 7.1: Introduction

[Introduction] I am not actually the primary caregiver, but I make sure that everything runs smoothly in the care of our mother. Since I have a job and a family of my own, the actual caregiving is handled by my sister and, most importantly, by the care services I have hired. I try to take responsibility for organizing the care. Together with my mother, I decide what she needs and ensure that she gets the right help. I have also hired professional caregivers to support me and my sister.

[Problem Description] It is important to me that everything is well organized, but I am not very emotionally involved in the care. I see it as a task that needs to be done, so I plan everything accordingly. I make sure my mother gets everything she needs, but I don’t let caregiving take too much of a toll on my own needs.

[Closing] This setup works quite well. I can organize everything without having to deal too much with the emotional aspects. However, sometimes my sister makes me feel like I am "over-organizing" and ignoring the emotional aspects of my mother’s dementia and her health condition. How can I ensure that the care remains well organized while also providing enough emotional support for my mother and my sister?

Persona 7: Persona who makes care decisions rationally and in an organized manner, at their own discretion, in alignment with their own life, with professional support

Story 7.2: Care and organisation

[Introduction] I see caregiving as an organizational task. I make sure that appointments are kept, the right people are available for support, and everything my mother needs is properly arranged. My goal is to coordinate everything so that nothing gets forgotten. My sister, however, sees things very differently. She is too emotionally involved.

[Problem Description] I do not personally handle every single task, but I ensure that everything runs according to plan. I regularly communicate with the caregivers and make sure that everything is working well. I also accompany my mother to her doctor’s appointments. However, my sister sometimes accuses me of being too neutral about the situation and neglecting the emotional side of caregiving.

[Closing] I want everything to run smoothly, but maybe I should listen more to my mother’s needs and focus less on the organizational aspects. How can I find a better balance between managing the care and providing emotional support for my mother?

Persona 7: Persona who makes care decisions rationally and in an organized manner, at their own discretion, in alignment with their own life, with professional support

Story 7.3: Making decisions

[Introduction] I make the decisions regarding my mother's care, but without letting it affect me emotionally. I involve the family and the caregivers, but I am the one who sets the direction. It feels good because I have control, but sometimes I wonder if I talk to my mother enough to truly understand how she feels.

[Problem Description] It is important that everything functions well, but I don't constantly focus on the emotional aspects. I make pragmatic decisions about what is best for her. However, with this approach, I might overlook something that is important to my mother, even if it is not directly related to caregiving.

[Closing] I know that I am good at organizing, but I wonder if I should pay more attention to my mother's emotional needs when making care decisions. I also question whether I should involve my sister more, as she spends a lot of time with our mother. How can I better recognize when my mother needs emotional support while still keeping the practical organization on track?

Persona 7: Persona who makes care decisions rationally and in an organized manner, at their own discretion, in alignment with their own life, with professional support

Story 7.4: Professional support

[Introduction] I have hired professional caregivers to handle the daily tasks. They are well-trained and can assist my mother wherever she needs help. I am always available when it comes to making decisions, but I let the caregivers take care of the practical work. This also relieves my sister.

[Problem Description] I manage the financial and organizational aspects while leaving the caregiving tasks to the professionals. Everything runs smoothly, but sometimes I feel disconnected from the daily care process.

[Closing] I want to ensure that my mother receives the best possible help. However, at times, I feel somewhat detached from the emotional connection that other caregivers or my sister have. How can I become more involved in daily care without taking on all the responsibility?

Persona 7: Persona who makes care decisions rationally and in an organized manner, at their own discretion, in alignment with their own life, with professional support

Story 7.5: Finding emotional balance

[Introduction] It’s not easy for me to get emotional when caring for my mother. I organize everything, but when it comes to feelings, I am not very involved. I make sure she is in good hands and receives the right support, but sometimes I wonder if I am doing enough for her emotional needs. I also question whether it is normal that I feel so detached from the fact that my mother is ill and needs care.

[Problem Description] I notice that I sometimes keep my distance when it comes to the emotional aspects. Maybe because I don’t want to deal with my own fears and worries. I take care of the finances and planning, but I don’t often engage in conversations about her feelings or wishes. I want to ensure she is well cared for, but I wonder if I am not listening enough to what she truly needs.

[Closing] Maybe I should take more time to truly address her emotional needs, even though it’s not my strength. How can I better integrate the emotional aspects of caregiving into my daily life without neglecting my organizational role?

Persona 8: Persona who actively disengages from the caring situation, playing a maximally supportive, rather observing role

Overview Story Pack

Story	Thema
Story 8.1	Introduction
Story 8.2	Support in care
Story 8.3	Distinction from care
Story 8.4	Family support
Story 8.5	Role in care

Persona 8: Persona who actively disengages from the caring situation, playing a maximally supportive, rather observing role

Story 8.1: Introduction

[Introduction] I help where I can, but I'm mostly on the outside. The care of my mother is primarily my sister's responsibility. I support her when needed, but I am not really involved in the daily caregiving routine. I focus more on other things and leave the caregiving to my sister.

[Problem Description] I am there when she needs help, but I don't feel very responsible for the care. There are always tasks I take on, like shopping or cooking, but the care itself is not really my thing. I'm not sure how I could contribute more without getting too involved.

[Closing] I wonder if I should do more, but somehow I don't really feel responsible. I try to support the care, but not actively participate. How can I change my role in caregiving without feeling overwhelmed?

Persona 8: Persona who actively disengages from the caring situation, playing a maximally supportive, rather observing role

Story 8.2: Support in care

[Introduction] I'm sometimes there when help is needed, but honestly, I don't know much about caregiving. My sister handles almost everything. I take care of practical support, like getting groceries or organizing doctor appointments. But when it comes to the care itself, I don't have much knowledge.

[Problem Description] I often feel uncertain about what the right help is. I wonder if I'm doing enough or if I should be more involved. But somehow, I lack the knowledge to really help actively. It's hard for me to understand what my mother needs, especially when it comes to the caregiving itself.

[Closing] I want to understand more about what my mother needs, but I don't know where to start. Maybe I should educate myself more. How can I learn more about caregiving without overwhelming myself?

Persona 8: Persona who actively disengages from the caring situation, playing a maximally supportive, rather observing role

Story 8.3: Distinction from care

[Introduction] I help when necessary, but my family mainly takes care of the caregiving. I'm there when they need me, but I don't feel responsible for everything. There are situations where I'm asked to take over something, but I find it hard to really become active. After all, it's my mother who is ill.

[Problem Description] I often think that other family members could do more, but I feel like I'm not the one who should constantly intervene in the caregiving. I want to support, but I notice that I emotionally distance myself. It's difficult for me to fully engage in caregiving, and I often feel that my family can handle it better among themselves. On the other hand, I want to support and relieve my family, and I don't want it to seem like I don't care about the situation. But I'm actually content with the situation.

[Closing] I'm there when needed, but somehow, I feel like I can't really change much when it comes to my mother's care. I do want to help. How can I help without getting too involved in caregiving?

Persona 8: Persona who actively disengages from the caring situation, playing a maximally supportive, rather observing role

Story 8.4: Family support

[Introduction] I help when necessary, but my family mainly takes care of the caregiving. I'm there when they need me, but I don't feel responsible for everything. There are situations where I'm asked to take over something, but I find it hard to really become active. After all, it's my mother who is ill.

[Problem Description] I often think that other family members could do more, but I feel like I'm not the one who should constantly intervene in the caregiving. I want to support, but I notice that I emotionally distance myself. It's difficult for me to fully engage in caregiving, and I often feel that my family can handle it better among themselves. On the other hand, I want to support and relieve my family, and I don't want it to seem like I don't care about the situation. But I'm actually content with the situation.

[Closing] I'm there when needed, but somehow, I feel like I can't really change much when it comes to my mother's care. I do want to help. How can I help without getting too involved in caregiving?

Persona 8: Persona who actively disengages from the caring situation, playing a maximally supportive, rather observing role

Story 8.5: Role in care

[Introduction] I see my mother's care more from a structural perspective. There are specific tasks that need to be done, and I am here to support those tasks. But when it comes to how my mother is doing or how everyone is feeling, I tend to be more distant. I do what I need to do, but nothing more.

[Problem Description] There are moments when I realize that I withdraw from caregiving because I don't want to be as involved with the feelings or the emotional aspect of caregiving. I handle organizational things, like medical care, but I leave my mother's emotional needs to others.

[Closing] I find it difficult to deal with the emotional side of caregiving. I do what is necessary, but it doesn't feel like I'm truly engaged in the caregiving. How can I define my role in caregiving more clearly while not feeling overwhelmed?

Persona 9: Persona who is informed about dementia and care and therefore trusts only professionals and sees himself as a committed knowledge broker

Overview Story Pack

Story	Thema
Story 9.1	Introduction
Story 9.2	Trust in experts
Story 9.3	Control and overload in care
Story 9.4	Social and cultural expectations
Story 9.5	Knowledge and support

Persona 9: Persona who is informed about dementia and care and therefore trusts only professionals and sees himself as a committed knowledge broker

Story 9.1: Introduction

[Introduction] I am the primary caregiver for my mother, who has dementia. I have invested a lot of time learning about the disease and caregiving because I want to ensure she receives the best care. When it comes to her care, I only trust professional experts – doctors, nurses, and specialized counseling services.

[Problem Description] I find it difficult to trust other family members when it comes to caregiving. I know a lot about dementia and caregiving, but often I feel that others don't know enough. Sometimes I receive advice from my family, but I'm not sure they truly understand what my mother needs. I try to meet her needs as best as I can, but managing everything is difficult.

[Closing] I realize that handling everything on my own is overwhelming. But even when I seek help from outside, I don't fully trust anyone. I want to maintain control and ensure everything is done correctly. How can I learn to trust others more without losing control?

Persona 9: Persona who is informed about dementia and care and therefore trusts only professionals and sees himself as a committed knowledge broker

Story 9.2: Trust in experts

[Introduction] I regularly get in touch with doctors, nurses, and dementia counseling services to ensure that we always receive the best information and support. In my family, I am the one who makes decisions about caregiving because I have the most experience and knowledge. The other family members are aware of this and trust me because of it. They gladly entrust me with the responsibility of caregiving.

[Problem Description] On the other hand, they often don't understand why I insist on professional help. They think I could manage the caregiving on my own without external support. But I know that dementia is a disease where experts are very important. There are many things I don't know, and I don't want to risk my mother receiving incorrect treatment.

[Closing] It's sometimes difficult to convince my family that professional help is necessary. But I stand by my belief that professionals have more knowledge and know better what's important for my mother. How can I better explain to my family why I insist on professional help?

Persona 9: Persona who is informed about dementia and care and therefore trusts only professionals and sees himself as a committed knowledge broker

Story 9.3: Control and overload in care

[Introduction] Since I am the primary caregiver for my mother, I carry most of the responsibility. I organize everything – from doctor visits to daily caregiving tasks. Sometimes it feels like everything depends on me. It's hard to manage all of this, especially with my own family and work.

[Problem Description] I often feel like I have to maintain control over everything to ensure things are done correctly. However, there are also moments when I feel overwhelmed. It's hard to find a balance between my caregiving duties and my own life. I want to give everything to my mother, but there's barely any time left for myself.

[Closing] It’s a constant challenge to keep everything together. But I am determined to provide the best care for my mother, even though it's exhausting. How can I better organize the caregiving without losing myself?

Persona 9: Persona who is informed about dementia and care and therefore trusts only professionals and sees himself as a committed knowledge broker

Story 9.4: Social and cultural expectations

[Introduction] In my family, there is a strong tradition of caring for the older generations. I understand this tradition, but at the same time, I have realized that dementia requires a different type of care that not everyone understands. I try to respect the cultural expectations, but I know that my mother's care needs to take place on a different level.

[Problem Description] There are situations where my family expects me to care for my mother in a certain way. But I know that this isn't always the best solution. For example, some traditional caregiving rituals will no longer work when my mother is no longer able to help herself. I try to find this balance – respecting the tradition, but also ensuring the right care.

[Closing] It’s difficult to reconcile both worlds – the traditional values of my family and what my mother truly needs. But I try to make the best of both aspects. How can I align my family's cultural expectations with my mother's needs?

Persona 9: Persona who is informed about dementia and care and therefore trusts only professionals and sees himself as a committed knowledge broker

Story 9.5: Knowledge and support

[Introduction] Since I know so much about dementia and caregiving, I enjoy sharing this knowledge with others. I think it's important that everyone understands what my mother needs and how she should be properly cared for. But sometimes, even though I know that outside support could be very helpful, I find it difficult to ask for help myself.

[Problem Description] I talk a lot about dementia, but it feels like no one truly understands how difficult it is. Even though I know a lot, I can't do everything on my own. But I also have trouble accepting the help offered to me because I'm not sure if the other people have as much knowledge as I do.

[Closing] It's a constant balancing act between sharing knowledge and accepting help. But I know I need support, even if it's hard for me to accept. Maybe I should share my knowledge more and then accept help when I know the others are on my level of understanding. How can I learn to share my knowledge and then accept support that can ease my burden?
