# Supplementary material for: Storytelling in family-provided care - developing diversity-sensitive stories for family caregivers of Turkish individuals living with dementia
Source: BMC Nurs. 2025 Aug 12;24:1060. doi: 10.1186/s12912-025-03712-7 (PMC12341143; doi:10.1186/s12912-025-03712-7)
Supplement: Supplementary file 2 — Supplementary Material 2 [file 12912_2025_3712_MOESM2_ESM.pdf]

Persona 1: Persona, die in einem Rollenkonflikt und unter mehrfachem Druck steht und die Anforderungen der Berufstätigkeit mit der Pflege des erkrankten Familienmitglieds und der eigenen Familienaufrechterhaltung in Einklang bringen muss

Übersicht Storypaket

Story	Thema
Story 1.1	Einführung
Story 1.2	Stressbewältigung und Zeitmanagement
Story 1.3	Familiäre Unterstützung und Konfliktlösung
Story 1.4	Zugang und Nutzung professioneller Entlastungsangebote
Story 1.5	Herstellung einer Care-Life-Balance

Persona 1: Persona, die in einem Rollenkonflikt und unter mehrfachem Druck steht und die Anforderungen der Berufstätigkeit mit der Pflege des erkrankten Familienmitglieds und der eigenen Familienaufrechterhaltung in Einklang bringen muss

Story 1.1: Einführung

[Einführung] Ich kümmere mich alleine um meine Mutter. Ich arbeite Vollzeit in einer Bäckerei und habe zwei Töchter. Manchmal ist es sehr schwer für mich, alles zu schaffen. Ich bekomme eigentlich keine Hilfe von meinen Geschwistern. Die anderen in der Familie glauben nicht, dass unsere Mutter krank ist. Sie sagen: „Mama sieht doch normal aus.“ oder „Du übertreibst.“.

[Problembeschreibung] Deshalb ist es sehr schwer für mich, alles unter einen Hut zu bekommen. Ich habe das Gefühl, ich kann niemandem gerecht werden. Und mir selber werde ich schon lange nicht mehr gerecht. Ich weiß, dass ich Hilfe brauche, um alles zu stemmen. Aber ich habe so wenig Zeit. Wie soll ich da noch ein Entlastungsangebot nutzen? Ich bin froh, wenn ich überhaupt Zeit für die Pflege von Mama finde.

[Abschluss] Manchmal will ich einfach nur noch abschalten oder einen Pause-Knopf drücken. Wenn ich mal entspannen kann und nicht an die Krankheit von Mama denken muss, ist das wie Urlaub für mich. Dass ich so gestresst bin, führt auch immer wieder zu Streitereien mit meinem Mann oder mit meinen Töchtern. Das ist sehr belastend für mich. Ich weiß einfach nicht, wie ich gleichzeitig Pflegende, Ehefrau, Mutter und Beschäftigte sein soll. Was kann ich tun, um nicht ständig unter Druck zu stehen und mich trotz wenig Zeit zu entlasten?

Persona 1: Persona, die in einem Rollenkonflikt und unter mehrfachem Druck steht und die Anforderungen der Berufstätigkeit mit der Pflege des erkrankten Familienmitglieds und der

eigenen Familienaufrechterhaltung in Einklang bringen muss

Story 1.2: Stressbewältigung und Zeitmanagement

[Einführung] Manchmal habe ich das Gefühl, dass mein Tag viel zu wenige Stunden hat. Zwischen meiner Arbeit in der Bäckerei, der Pflege meiner Mutter und meiner Rolle als Mutter für meine Töchter bleibt kaum Zeit für mich selbst. Oft fange ich schon morgens an, To-do-Listen im Kopf durchzugehen, und fühle mich gestresst, bevor der Tag überhaupt richtig begonnen hat.

[Problembeschreibung] Ich merke, dass der Stress nicht nur mich, sondern auch mein Umfeld belastet. Ich werde schneller gereizt und habe keine Energie mehr, mit meinen Töchtern oder meinem Mann Zeit zu verbringen. Ein Kollege meinte einmal, ich sollte „lernen, Prioritäten zu setzen", aber ich weiß nicht, wie das gehen soll. Es scheint alles wichtig zu sein – Pflege, Haushalt, Arbeit. Wie soll ich da entscheiden, was liegen bleiben kann?

[Abschluss] Neulich habe ich ein Buch über Zeitmanagement gelesen. Es hat mich auf die Idee gebracht, feste Zeiten für bestimmte Aufgaben einzuplanen und mir bewusst kleine Pausen zu gönnen. Ich frage mich, ob das bei mir funktionieren könnte. Wie kann ich meinen Alltag so strukturieren, dass ich weniger gestresst bin und trotzdem alles schaffe, was wichtig ist?

Persona 1: Persona, die in einem Rollenkonflikt und unter mehrfachem Druck steht und die

Anforderungen der Berufstätigkeit mit der Pflege des erkrankten Familienmitglieds und der eigenen Familienaufrechterhaltung in Einklang bringen muss

Story 1.3: Familiäre Unterstützung und Konfliktlösung

[Einführung] Ich bin immer noch die Einzige in meiner Familie, die sich um Mama kümmert. Meine Geschwister sind der Meinung, dass sie „gar nicht so krank aussieht". Kürzlich hatte ich wieder eine Diskussion mit meinem Bruder. Ich habe ihn gebeten, Mama wenigstens ab und zu für ein paar Stunden zu besuchen, damit ich etwas Zeit für mich habe.

[Problembeschreibung] Seine Antwort hat mich wütend gemacht: „Du machst das doch gut. Warum soll ich mich einmischen?" Ich fühle mich so allein gelassen. Es verletzt mich, dass meine Familie nicht versteht, wie anstrengend das alles für mich ist. Gleichzeitig habe ich Angst, dass solche Konflikte die Beziehungen in unserer Familie dauerhaft belasten.

[Abschluss] Nach diesem Streit habe ich mich gefragt, wie ich meine Geschwister dazu bringen könnte, mich besser zu unterstützen, ohne dass es jedes Mal in Streit endet. Vielleicht müsste ich ihnen klarer machen, wie es mir wirklich geht und was ich brauche. Wie kann ich meiner Familie meine Situation verständlich machen, ohne dass es immer wieder zu Konflikten kommt?

Persona 1: Persona, die in einem Rollenkonflikt und unter mehrfachem Druck steht und die Anforderungen der Berufstätigkeit mit der Pflege des erkrankten Familienmitglieds und der eigenen Familienaufrechterhaltung in Einklang bringen muss

Story 1.4: Zugang und Nutzung professioneller Entlastungsangebote

[Einführung] Letzte Woche habe ich bei der Arbeit mit einer Kollegin gesprochen, die mir von einem Pflegedienst in unserer Nähe erzählt hat. Sie meinte, dass sie dort Unterstützung für ihre Schwiegermutter gefunden hat. Ich habe darüber nachgedacht, ob das auch etwas für uns wäre, aber ich weiß nicht, ob ich das in meinen Alltag integrieren kann.

[Problembeschreibung] Eigentlich brauche ich dringend Entlastung, aber ich habe das Gefühl, dafür fehlt mir die Zeit. Die Pflege von Mama ist so schon schwer genug, und jetzt auch noch einen Termin mit einem Pflegedienst zu organisieren, erscheint mir unmöglich. Außerdem habe ich Bedenken, ob der Pflegedienst wirklich zu uns passt. Ich habe gehört, dass viele Pflegekräfte kein Türkisch sprechen. Mama versteht kaum Deutsch, wie soll sie sich da mit ihnen verständigen? Außerdem habe ich Angst, dass sie Dinge nicht berücksichtigen, die für Mama wichtig sind – zum Beispiel, dass sie kein Schweinefleisch isst. Mama hat mir auch gesagt, dass sie keine „fremden Leute“ im Haus möchte. Ich weiß nicht, wie ich ihr erklären soll, dass wir diese Unterstützung brauchen.

[Abschluss] Ich frage mich, ob so ein Pflegedienst wirklich helfen könnte und wie ich Mama überzeugen kann, es wenigstens auszuprobieren. Vielleicht sollte ich vorher fragen, ob die Pflegekräfte auf solche Dinge wie Sprache und Ernährung Rücksicht nehmen können oder ob es sogar türkische Pflegekräfte gibt. Doch wie finde ich einen Pflegedienst, der unsere Bedürfnisse versteht, und wie kann ich meine Mutter davon überzeugen, ihn auszuprobieren?

Persona 1: Persona, die in einem Rollenkonflikt und unter mehrfachem Druck steht und die Anforderungen der Berufstätigkeit mit der Pflege des erkrankten Familienmitglieds und der eigenen Familienaufrechterhaltung in Einklang bringen muss

Story 1.5: Herstellung einer Care-Life-Balance

[Einführung] Manchmal frage ich mich, wie ich es schaffen soll, all meinen Rollen gerecht zu werden. Ich bin Pflegende, Mutter, Ehefrau, Arbeitnehmerin – aber wo bleibe ich selbst? Vor kurzem hat meine Tochter mich gefragt, warum ich so selten Zeit für sie habe. Es hat mich sehr getroffen, das zu hören.

[Problembeschreibung] Ich merke, dass ich ständig versuche, alle Erwartungen zu erfüllen, aber dabei gehe ich selbst verloren. Wenn ich mehr Zeit mit meinen Töchtern verbringe, bleibt die Pflege liegen. Wenn ich mich auf die Pflege konzentriere, leidet meine Beziehung zu meinem Mann. Es fühlt sich an, als könnte ich es niemandem recht machen.

[Abschluss] Ich habe angefangen, darüber nachzudenken, was ich wirklich will und welche Prioritäten ich setzen sollte. Vielleicht müsste ich lernen, meine Grenzen klarer zu kommunizieren und mehr Unterstützung einzufordern, aber das fällt mir schwer. Wie kann ich es schaffen, meine verschiedenen Rollen besser auszubalancieren und trotzdem noch Zeit für mich selbst zu finden?

Persona 2: Persona, die sich einsam, überfordert und belastet fühlt durch familiäre Konflikte und das Schicksal der Krankheit ihres Familienmitglieds

Übersicht Storypaket

Story	Thema
Story 2.1	Einführung
Story 2.2	Umgang mit Einsamkeit
Story 2.3	Überforderung in der Pflege
Story 2.4	Religiöser Halt und innere Stärke
Story 2.5	Verlust der eigenen Identität

Persona 2: Persona, die sich einsam, überfordert und belastet fühlt durch familiäre Konflikte und das Schicksal der Krankheit ihres Familienmitglieds

Story 2.1: Einführung

[Einführung] Ich pflege meinen Vater alleine, seit meine Mutter vor drei Jahren gestorben ist. Es gibt niemanden, der mir helfen könnte, und ich sehe die Pflege als meine Aufgabe vor Gott. Mein Vater hat mir das Leben geschenkt, und jetzt ist es meine Pflicht, für ihn da zu sein. Trotzdem merke ich immer öfter, wie schwer diese Verantwortung auf meinen Schultern lastet.

[Problembeschreibung] Ich habe inzwischen auch keinen richtigen Kontakt zu Menschen außerhalb der Familie, und meine Tage verlaufen immer gleich. Ich wache auf, kümmere mich um meinen Vater, mache den Haushalt und erledige alles, was anfällt. Es bleibt keine Zeit für mich selbst, und manchmal frage ich mich, wer ich eigentlich bin, außer die Pflegende. Ich bete jeden Tag, dass ich die Kraft finde, weiterzumachen, aber oft fühle ich mich überfordert und allein.

[Abschluss] Manchmal wünschte ich, ich hätte jemanden, mit dem ich reden könnte – jemanden, der versteht, wie schwer es ist, alleine alles zu tragen. Doch ich habe gelernt, meine Gefühle für mich zu behalten, denn es gehört sich nicht, zu klagen. Wie kann ich in meiner Situation Erleichterung finden, ohne meine Verantwortung oder meine Werte zu vernachlässigen?

Persona 2: Persona, die sich einsam, überfordert und belastet fühlt durch familiäre Konflikte und das Schicksal der Krankheit ihres Familienmitglieds

Story 2.2: Umgang mit Einsamkeit

[Einführung] Manchmal merke ich, wie still es um mich herum ist. Mein Vater spricht kaum noch, und ich habe niemanden, mit dem ich mich austauschen kann. Früher hatte ich Freundinnen, aber ich habe den Kontakt verloren, weil ich immer zu Hause bin.

[Problembeschreibung] Ich weiß, dass Einsamkeit mir nicht guttut, aber ich fühle mich machtlos, etwas daran zu ändern. Manchmal träume ich davon, einfach mit jemandem zu reden oder sogar jemanden zu treffen, der eine ähnliche Situation erlebt. Doch ich wüsste gar nicht, wie ich das machen soll, ohne die Pflege zu vernachlässigen.

[Abschluss] Ich frage mich, ob es Möglichkeiten gibt, trotz meiner Situation wieder Kontakte zu knüpfen. Vielleicht würde es mir helfen, mich weniger allein zu fühlen. Wie kann ich meine Einsamkeit überwinden, ohne meine Aufgaben zu vernachlässigen?

Persona 2: Persona, die sich einsam, überfordert und belastet fühlt durch familiäre Konflikte und das Schicksal der Krankheit ihres Familienmitglieds

Story 2.3: Überforderung in der Pflege

[Einführung] Neulich hat mein Vater mich nachts gerufen, weil er Schmerzen hatte. Ich habe ihm geholfen, aber danach konnte ich nicht mehr schlafen. Solche Nächte kommen immer häufiger vor, und ich merke, wie mir die Energie ausgeht.

[Problembeschreibung] Die Pflege wird immer anstrengender, und ich weiß oft nicht, wie ich damit umgehen soll. Es gibt Momente, in denen ich das Gefühl habe, alles wächst mir über den Kopf. Ich mache mir Sorgen, dass ich nicht genug tue, obwohl ich schon alles gebe.

[Abschluss] Manchmal frage ich mich, ob es jemanden gibt, der mir zeigen kann, wie ich die Pflege besser bewältigen kann, ohne selbst daran kaputtzugehen. Aber ich kenne ja niemanden und ich wüsste auch nicht, wie es besser gehen sollte. Wie kann ich mit der Überforderung umgehen und gleichzeitig die Pflege meines Vaters sicherstellen?

Persona 2: Persona, die sich einsam, überfordert und belastet fühlt durch familiäre Konflikte und das Schicksal der Krankheit ihres Familienmitglieds

Story 2.4: Religiöser Halt und innere Stärke

[Einführung] Ich finde Trost in meinem Glauben. Jeden Tag bete ich dafür, dass Allah mir die Kraft gibt, meinen Vater zu pflegen. Ich sehe die Pflege als meine Aufgabe und auch als einen Weg, meinen Glauben zu leben.

[Problembeschreibung] Trotz meines Glaubens habe ich manchmal Zweifel. Es gibt Tage, an denen ich mich frage, ob ich genug tue oder ob ich meinem Vater gerecht werde. Diese Gedanken lassen mich nicht los, und ich fühle mich schuldig, wenn ich merke, dass mir die Kraft fehlt.

[Abschluss] Ich frage mich, wie ich meinen Glauben noch stärker nutzen kann, um die Herausforderungen der Pflege zu meistern. Vielleicht gibt es Wege, meine Aufgaben mit mehr innerem Frieden zu erfüllen. Wie kann mein Glaube mir helfen, in schwierigen Momenten stärker zu sein?

Persona 2: Persona, die sich einsam, überfordert und belastet fühlt durch familiäre Konflikte und das Schicksal der Krankheit ihres Familienmitglieds

Story 2.5: Verlust der eigenen Identität

[Einführung] Seit ich meinen Vater pflege, habe ich mein Leben komplett verändert. Eigentlich nicht mit Absicht. Früher hatte ich Hobbys und Träume, aber jetzt geht es nur noch um die Pflege. Ich habe keine Zeit für Freunde und keine Lust, neue Dinge auszuprobieren. Ich weiß nicht mehr, wer ich außerhalb dieser Rolle bin.

[Problembeschreibung] Es fühlt sich an, als hätte ich mich selbst verloren. Ich vermisse die Person, die ich einmal war, aber ich weiß nicht, wie ich dorthin zurückfinden kann, ohne meine Pflicht zu vernachlässigen. Manchmal denke ich, dass ich nie wieder Zeit für mich selbst haben werde.

[Abschluss] Ich frage mich, ob es möglich ist, etwas von meinem alten Leben zurückzugewinnen, ohne meine Aufgabe als Pflegende aufzugeben. Vielleicht gibt es einen Weg, beides zu verbinden. Wie kann ich wieder ein Gefühl für mich selbst entwickeln und gleichzeitig für meinen Vater da sein?

Persona 3: Persona, die stark belastet ist, unter unterschiedlichen Ansichten über Demenz und den Umgang damit innerhalb der Familie leidet und die Pflege nicht so organisieren kann, wie sie es gerne würde

Übersicht Storypaket

Story	Thema
Story 3.1	Einführung
Story 3.2	Unterschiedliche Ansichten in der Familie
Story 3.3	Umgang mit familiären Konflikten
Story 3.4	Abgrenzung von Stigmata bezüglich Demenz
Story 3.5	Umgang mit Machtlosigkeit

Persona 3: Persona, die stark belastet ist, unter unterschiedlichen Ansichten über Demenz und den Umgang damit innerhalb der Familie leidet und die Pflege nicht so organisieren kann, wie sie es gerne würde

Story 3.1: Einführung

[Einführung] Ich unterstütze meinen Vater bei der Pflege meiner Mutter, die an Demenz leidet. Obwohl ich nicht die Hauptpflegeperson bin, versuche ich, so viel wie möglich zu helfen. Leider gibt es in unserer Familie ständig Konflikte darüber, wie wir mit Mamas Krankheit umgehen sollen.

[Problembeschreibung] Mein Vater glaubt, dass Mama einfach mehr Zuwendung braucht und dass sie „sich zusammenreißen“ könnte. Meine Schwester hingegen meint, wir sollten sie mehr in Ruhe lassen und aufhören, so viel Drama zu machen. Ich bin der Meinung, dass wir professionelle Hilfe brauchen, aber das stößt auf Widerstand. Es fühlt sich an, als würde niemand in der Familie mich wirklich ernst nehmen. Ich weiß, dass ich nicht alles ändern kann, aber ich möchte, dass wir Mama die bestmögliche Pflege bieten können – ohne ständig miteinander zu streiten.

[Abschluss] Manchmal habe ich das Gefühl, zwischen allen Fronten zu stehen. Ich sehe, wie meine Mutter leidet, aber ich kann nichts tun, um die Pflege so zu gestalten, wie ich es für richtig halte. Wie kann ich meine Familie dazu bringen, an einem Strang zu ziehen, und wie kann ich meine Rolle in der Pflege besser wahrnehmen?

Persona 3: Persona, die stark belastet ist, unter unterschiedlichen Ansichten über Demenz und den Umgang damit innerhalb der Familie leidet und die Pflege nicht so organisieren kann, wie sie es gerne würde

Story 3.2: Unterschiedliche Ansichten in der Familie

[Einführung] Gestern hatten wir wieder eine hitzige Diskussion über Mama. Mein Vater glaubt, dass wir ihr keine Medikamente geben sollten, weil er meint, das würde alles nur schlimmer machen. Meine Schwester sieht das genauso, aber ich bin anderer Meinung.

[Problembeschreibung] Es ist so schwierig, sich in der Familie auf einen gemeinsamen Weg zu einigen. Ich habe das Gefühl, dass wir alle etwas anderes wollen und dabei vergessen, was Mama wirklich braucht. Ich bin überzeugt, dass wir mit ärztlicher Unterstützung besser zurechtkommen würden, aber ich werde überstimmt oder gar nicht erst ernst genommen. Ich habe Angst, dass es Mama schlechter gehen wird, wenn wir nicht etwas unternehmen.

[Abschluss] Manchmal frage ich mich, ob ich überhaupt etwas bewirken kann. Ich möchte das Beste für Mama, aber wie soll das gehen, wenn wir uns ständig uneinig sind? Wie kann ich meiner Familie klar machen, dass wir professionellen Rat einholen sollten, ohne dass es zu weiteren Konflikten kommt?

Persona 3: Persona, die stark belastet ist, unter unterschiedlichen Ansichten über Demenz und den Umgang damit innerhalb der Familie leidet und die Pflege nicht so organisieren kann, wie sie es gerne würde

Story 3.3: Umgang mit familiären Konflikten

[Einführung] Nach unserem letzten Streit habe ich versucht, mit meiner Schwester in Ruhe zu reden. Ich wollte verstehen, warum sie so gegen meinen Vorschlag war, eine Pflegeberatung in Anspruch zu nehmen. Ihre Antwort hat mich überrascht: Sie sagte, sie fühlt sich überfordert und hat Angst, dass wir Mama in ein Heim geben könnten.

[Problembeschreibung] Ich habe gemerkt, dass unsere Konflikte oft aus Ängsten und Unsicherheiten entstehen. Aber anstatt ehrlich darüber zu sprechen, werfen wir uns gegenseitig vor, was wir alles falsch machen. Das belastet mich sehr, denn ich will, dass wir gemeinsam eine Lösung finden – für Mama und für uns als Familie.

[Abschluss] Vielleicht müssten wir alle besser lernen, miteinander zu reden, ohne uns gegenseitig Vorwürfe zu machen. Aber wie fängt man so ein Gespräch an, wenn alle so angespannt sind? Wie können wir die familiären Spannungen abbauen und offener über unsere Gefühle und Ängste sprechen?

Persona 3: Persona, die stark belastet ist, unter unterschiedlichen Ansichten über Demenz und den Umgang damit innerhalb der Familie leidet und die Pflege nicht so organisieren kann, wie sie es gerne würde

Story 3.4: Abgrenzung von Stigmata bezüglich Demenz

[Einführung] Vor ein paar Tagen hat eine Nachbarin mich gefragt, warum meine Mutter sich so merkwürdig verhält. Ich habe ihr erklärt, dass sie an Demenz leidet, aber ihre Reaktion hat mich getroffen: „Das ist doch bestimmt schlimm für euch – hoffentlich merken die Leute das nicht zu sehr."

[Problembeschreibung] Ich habe das Gefühl, dass viele Menschen in unserer Umgebung nicht verstehen, was Demenz bedeutet. Es ist, als wäre die Krankheit ein Tabu, über das man nicht sprechen darf. Mein Vater will nicht, dass jemand außerhalb der Familie davon erfährt, und meine Schwester geht solchen Gesprächen komplett aus dem Weg. Aber ich finde, wir sollten offen damit umgehen, um Vorurteile und Stigmata abzubauen. Auch innerhalb der Familie tun wir uns schwer, offen über die Krankheit zu sprechen.

[Abschluss] Ich frage mich, wie wir einen Weg finden können, über die Krankheit zu sprechen, ohne uns dabei schlecht oder verurteilt zu fühlen. Vielleicht würde uns das auch helfen, als Familie besser damit umzugehen. Wie können wir offen mit Demenz umgehen und gleichzeitig die Sorgen und Ängste anderer Menschen in der Familie berücksichtigen?

Persona 3: Persona, die stark belastet ist, unter unterschiedlichen Ansichten über Demenz und den Umgang damit innerhalb der Familie leidet und die Pflege nicht so organisieren kann, wie sie es gerne würde

Story 3.5: Umgang mit Machtlosigkeit

[Einführung] Ich habe immer wieder das Gefühl, dass ich nichts ändern kann. Egal wie sehr ich mich bemühe, die Entscheidungen treffen letztlich andere, und ich bleibe zurück mit meinen Sorgen und meiner Frustration.

[Problembeschreibung] Es fällt mir schwer, damit umzugehen, dass ich in der Pflege oft nur eine Nebenrolle spiele. Ich sehe, dass Dinge falsch laufen, aber wenn ich darauf hinweise, werde ich ignoriert oder übergangen. Das Gefühl, machtlos zu sein, belastet mich sehr. Ich frage mich, ob ich jemals in der Lage sein werde, wirklich etwas zu bewirken.

[Abschluss] Manchmal denke ich, dass ich lernen muss, loszulassen – aber wie soll ich das machen, wenn ich weiß, dass meine Mutter darunter leiden könnte? Wie kann ich mit meiner Machtlosigkeit umgehen und trotzdem einen positiven Beitrag zur Pflege leisten?

Persona 4: Persona, die die Rolle der Pflegeperson akzeptiert und ihre eigenen Bedürfnisse und ihr Wohlbefinden aus Liebe und im Einklang mit den Erwartungen anderer zurückstellt, um den sozialen Frieden zu wahren

Übersicht Storypaket

**Story**	**Thema**
Story 4.1	Einführung
Story 4.2	Verlust der eigenen Identität
Story 4.3	Akzeptanz von Unterstützung
Story 4.4	Emotionale Belastung durch Selbstaufopferung
Story 4.5	Umgang mit eigenen Bedürfnissen

Persona 4: Persona, die die Rolle der Pflegeperson akzeptiert und ihre eigenen Bedürfnisse und ihr Wohlbefinden aus Liebe und im Einklang mit den Erwartungen anderer zurückstellt, um den sozialen Frieden zu wahren

Story 4.1: Einführung

[Einführung] Ich habe mich entschieden, die Pflege für meine Mutter zu übernehmen, nachdem sie immer mehr Hilfe im Alltag benötigte. Sie lebt jetzt seit zwei Jahren bei mir, und ich kümmere mich rund um die Uhr um sie. Ich könnte nie daran denken, sie in ein Pflegeheim zu geben – es ist einfach nicht das Richtige für sie. In meiner Familie ist es Tradition, dass wir füreinander da sind, besonders in schwierigen Zeiten. Und als ihre Tochter ist es einfach meine Pflicht, mich um sie zu kümmern.

[Problembeschreibung] Manchmal frage ich mich, wie ich es überhaupt schaffe, noch zu funktionieren. Ich habe kaum noch Zeit für mich selbst und fühle mich oft erschöpft. Aber ich will nicht klagen – es ist schließlich meine Verantwortung, und ich habe diese Aufgabe mit Liebe übernommen. Ich empfinde es als Sünde, mir etwas Gutes zu tun, wenn meine Mutter meine Hilfe braucht. Aber manchmal wird es einfach zu viel, und ich weiß nicht, wie lange ich noch so weitermachen kann.

[Abschluss] Ich kann nicht aufhören, mich um sie zu kümmern – das würde sich falsch anfühlen. Aber gleichzeitig frage ich mich, wie lange ich das aufrechterhalten kann, ohne meine eigene Gesundheit und mein Wohlbefinden zu verlieren. Wie kann ich mit dieser ständigen Aufopferung umgehen, ohne meine eigene Identität und Lebensqualität komplett zu verlieren?

Persona 4: Persona, die die Rolle der Pflegeperson akzeptiert und ihre eigenen Bedürfnisse und ihr Wohlbefinden aus Liebe und im Einklang mit den Erwartungen anderer zurückstellt, um den sozialen Frieden zu wahren

Story 4.2: Verlust der eigenen Identität

[Einführung] In den letzten Monaten habe ich bemerkt, wie sehr sich mein Leben verändert hat. Früher hatte ich Hobbys, Freunde und meine eigenen Wünsche und Ziele. Doch jetzt dreht sich alles nur noch um die Pflege meiner Mutter. Es fühlt sich an, als hätte ich mich selbst verloren.

[Problembeschreibung] Ich frage mich oft, wer ich noch bin, wenn ich nicht die „Pflegende" bin. Ich kümmere mich um Mama, mache den Haushalt, und selbst mein Schlaf ist oft unterbrochen, weil sie nachts Hilfe braucht. Meine Freunde melden sich schon lange nicht mehr, und ich habe den Kontakt zu allem verloren, was mich früher einmal ausgemacht hat. Ich opfere alles, was ich hatte, um für sie da zu sein.

[Abschluss] Ich habe das Gefühl, dass ich mich vollkommen verloren habe. Ich weiß nicht, wie ich wieder zu mir selbst finden kann, wenn ich ständig nur für sie lebe. Wie kann ich meine eigene Identität zurückgewinnen, während ich immer noch für meine Mutter da bin?

Persona 4: Persona, die die Rolle der Pflegeperson akzeptiert und ihre eigenen Bedürfnisse und ihr Wohlbefinden aus Liebe und im Einklang mit den Erwartungen anderer zurückstellt, um den sozialen Frieden zu wahren

Story 4.3: Akzeptanz von Unterstützung

[Einführung] Mein Bruder hat mir gestern angeboten, Mama zum Arzt zu fahren, damit ich etwas Ruhe bekomme. Ich weiß, dass er es gut meint, aber ich konnte das Angebot nicht annehmen. Es fühlt sich einfach nicht richtig an, jemand anderen für meine Mutter sorgen zu lassen – dann kann ich auch nicht abschalten.

[Problembeschreibung] Es fällt mir unglaublich schwer, Hilfe anzunehmen. Ich denke immer, dass ich es besser machen kann und dass es meine Pflicht ist, sie allein zu pflegen. Manchmal ist es mir sogar unangenehm, um Hilfe zu bitten, weil ich glaube, dass es als Schwäche wahrgenommen wird. Ich habe das Gefühl, dass, wenn ich Hilfe annehme, ich meine Verantwortung aufgeben würde.

[Abschluss] Ich weiß, dass es mir guttun würde, mal eine Pause zu machen, aber ich kann einfach nicht loslassen. Es fühlt sich falsch an, mich selbst in den Vordergrund zu stellen. Wie kann ich lernen, Hilfe zu akzeptieren, ohne das Gefühl zu haben, meine Verantwortung zu verlieren?

Persona 4: Persona, die die Rolle der Pflegeperson akzeptiert und ihre eigenen Bedürfnisse und ihr Wohlbefinden aus Liebe und im Einklang mit den Erwartungen anderer zurückstellt, um den sozialen Frieden zu wahren

Story 4.4: Emotionale Belastung durch Selbstaufopferung

[Einführung] Ich habe das Gefühl, dass ich immer weniger von mir selbst übrig habe. Die emotionale Belastung ist enorm – ich versuche immer, stark zu sein und mich nicht anmerken zu lassen, wie erschöpft ich bin. Aber in den letzten Wochen merke ich, dass ich innerlich völlig ausgelaugt bin.

[Problembeschreibung] Es ist schwer, ständig für meine Mutter da zu sein, ohne jemals eine Pause zu haben. Ich möchte ihr so viel Liebe und Fürsorge geben, aber gleichzeitig spüre ich, wie die Last auf meinen Schultern immer schwerer wird. Ich weine manchmal heimlich, weil ich nicht weiß, wie ich es noch länger ertragen kann. Doch ich habe niemanden, mit dem ich darüber sprechen kann, und ich will nicht, dass jemand denkt, dass ich versage.

[Abschluss] Ich frage mich, wie lange ich diese Belastung noch ertragen kann. Es fühlt sich an, als ob ich nichts anderes mehr bin als die Pflegende – ohne Raum für meine eigenen Gefühle oder Bedürfnisse. Wie kann ich mit der emotionalen Last umgehen, ohne die Pflege aufzugeben oder meine Familie enttäuschen zu müssen?

Persona 4: Persona, die die Rolle der Pflegeperson akzeptiert und ihre eigenen Bedürfnisse und ihr Wohlbefinden aus Liebe und im Einklang mit den Erwartungen anderer zurückstellt, um den sozialen Frieden zu wahren

Story 4.5: Umgang mit eigenen Bedürfnissen

[Einführung] Es ist schwierig für mich, meine eigenen Bedürfnisse anzuerkennen. Ich weiß, dass ich manchmal eine Pause brauche, aber ich kann mich einfach nicht dazu durchringen, sie zu nehmen. Es fühlt sich an, als wäre es egoistisch, an mich selbst zu denken.

[Problembeschreibung] Jeder Tag dreht sich um die Bedürfnisse meiner Mutter – und ich habe das Gefühl, dass es keine Zeit für mich gibt. Ich habe kein Leben außerhalb der Pflege, keine Zeit für Freundschaften, keine Zeit für meine eigenen Interessen. Oft frage ich mich, wie es wäre, einfach mal ein paar Stunden für mich zu haben, aber dann wird mir sofort klar, dass ich das nicht tun kann. Es fühlt sich an, als ob meine eigenen Bedürfnisse unwichtig sind. Hauptsache Mama geht es gut und ich kann mich um sie kümmern.

[Abschluss] Ich frage mich, wie ich es schaffen kann, trotz dass ich mich gerne um meine Mutter kümmere, auch hin und wieder auf mich selbst zu achten, ohne mich schuldig zu fühlen. Wie kann ich lernen, meine eigenen Bedürfnisse zu erkennen und trotzdem die Pflege meiner Mutter mit Liebe zu übernehmen?

Persona 5: Persona, die vorrangig aufgrund günstiger Umstände, traditioneller/kultureller Normen, familiärer Erwartungen und hierarchischer Strukturen die Pflege übernimmt

Übersicht Storypaket

**Story**	**Thema**
Story 5.1	Einführung
Story 5.2	Pflege aus familiärer Pflicht
Story 5.3	Gesellschaftliche und kulturelle Erwartungen
Story 5.4	Geringe Unterstützung und Alleinverantwortung
Story 5.5	Selbstverwirklichung und Eigenleben

Persona 5: Persona, die vorrangig aufgrund günstiger Umstände, traditioneller/kultureller Normen, familiärer Erwartungen und hierarchischer Strukturen die Pflege übernimmt

Story 5.1: Einführung

[Einführung] Ich kümmere mich um meine Schwiegermutter, die an Demenz leidet. Es war nie eine Frage, ob ich diese Verantwortung übernehme – es war einfach klar, dass ich das tun muss. In unserer Familie ist es traditionell so, dass die jüngeren Generationen sich um die älteren kümmern, besonders wenn sie krank sind. Gerade die Frauen sind dafür zuständig. Mein Mann hat keine Schwestern und daher ist es meine Pflicht, mich um sie zu kümmern.

[Problembeschreibung] Ich habe nie wirklich darüber nachgedacht, wie ich mich dabei fühle. Es ist einfach so, wie es immer gemacht wurde. Aber je länger ich die Pflege übernehme, desto mehr merke ich, dass ich dabei oft auf meine eigenen Wünsche und Bedürfnisse verzichten muss. Es gibt keine Zeit für eigene Hobbys oder Freunde – alles dreht sich nur noch um die Pflege von meiner Schwiegermutter. Ich habe das Gefühl, dass ich dabei meine eigene Identität verliere.

[Abschluss] Es ist schwer, das Gefühl loszuwerden, dass ich nicht wirklich eine Wahl hatte, die Pflege zu übernehmen. Ich tue es aus Pflicht und Liebe zur Familie, aber manchmal frage ich mich, wie lange ich das noch so weitermachen kann, ohne meine eigenen Wünsche zu vernachlässigen. Wie kann ich die Balance zwischen meiner Verantwortung für meine Schwiegermutter und meinen eigenen Bedürfnissen finden, ohne das Gefühl zu haben, der familiären und gesellschaftlichen Erwartung nicht gerecht zu werden?

Persona 5: Persona, die vorrangig aufgrund günstiger Umstände, traditioneller/kultureller Normen, familiärer Erwartungen und hierarchischer Strukturen die Pflege übernimmt

Story 5.2: Pflege aus familiärer Pflicht

[Einführung] Es war nie eine Diskussion in der Familie, dass ich die Pflege für meine Schwiegermutter übernehmen würde. Es ist einfach die Art und Weise, wie es in unserer Kultur und Familie gehandhabt wird. Die Älteren haben sich immer um die Jüngeren gekümmert, und jetzt ist es an mir, dasselbe zu tun.

[Problembeschreibung] Manchmal frage ich mich, ob ich diese Entscheidung aus eigener Überzeugung getroffen habe oder einfach, weil es die familiäre Tradition verlangt. Es fühlt sich manchmal so an, als ob es keine Wahl gibt – wenn ich die Pflege nicht übernehme, würde das die ganze Familie enttäuschen. Aber in meinem Inneren frage ich mich, ob ich selbst glücklich bin mit dieser Verantwortung.

[Abschluss] Es gibt Momente, in denen ich mich frage, ob ich mich mehr auf mein eigenes Leben konzentrieren könnte. Doch die Verpflichtung gegenüber meiner Familie und die kulturellen Normen halten mich immer wieder zurück. Wie kann ich lernen, meinen eigenen Wünschen gerecht zu werden, ohne meine familiäre Verantwortung zu verletzen?

Persona 5: Persona, die vorrangig aufgrund günstiger Umstände, traditioneller/kultureller Normen, familiärer Erwartungen und hierarchischer Strukturen die Pflege übernimmt

Story 5.3: Gesellschaftliche und kulturelle Erwartungen

[Einführung] Meine Mutter hat mir immer gesagt, dass es meine Aufgabe sei, mich um die Familie zu kümmern, besonders wenn sie älter wird. In unserer Kultur wird es als selbstverständlich angesehen, dass die Kinder die Pflege der Eltern übernehmen. Und auch in unserer Gesellschaft wird es als Stärke angesehen, diese Verantwortung zu tragen. Ich kümmere mich gerne um Hilfebedürftige, aber ich hätte mich gerne bewusst dafür entschieden.

[Problembeschreibung] Ich fühle mich unter Druck gesetzt – sowohl durch die Erwartungen meiner Familie als auch durch die gesellschaftlichen Normen. Wenn ich Hilfe annehme oder mal eine Pause brauche, werde ich oft von anderen aus meiner Familie indirekt darauf hingewiesen, dass es meine Pflicht sei, stark zu sein und dass ich das schon schaffe. Es ist eine ständige Herausforderung, meine eigenen Bedürfnisse gegen diese Erwartungen durchzusetzen.

[Abschluss] Ich frage mich, ob ich jemals das Recht habe, einfach mal zu sagen: „Ich brauche eine Pause.“ und ob ich das überhaupt tun sollte, denn ich möchte ja auch, dass es meiner Schwiegermutter gut geht. Wie kann ich das Gefühl der Verpflichtung loswerden und mir selbst zugestehen, meine eigenen Bedürfnisse in den Vordergrund zu stellen?

Persona 5: Persona, die vorrangig aufgrund günstiger Umstände, traditioneller/kultureller Normen, familiärer Erwartungen und hierarchischer Strukturen die Pflege übernimmt

Story 5.4: Geringe Unterstützung und Alleinverantwortung

[Einführung] Ich bin die einzige, die sich um meine Schwiegermutter kümmern kann – niemand anderes in der Familie scheint wirklich in der Lage oder willens zu sein, sie zu unterstützen. Ich habe immer das Gefühl, alles allein machen zu müssen, was es noch schwieriger macht.

[Problembeschreibung] Es gibt kaum Unterstützung, obwohl ich das Gefühl habe, dass es meine Pflicht ist, alles zu erledigen. Mein Mann und seine Brüder sind entweder zu beschäftigt oder meinen, dass es nicht notwendig ist, sich einzumischen. Ich fühle mich oft, als müsste ich alles auf meinen Schultern tragen.

[Abschluss] Es ist eine große Last, die ich alleine trage, und manchmal frage ich mich, wie ich es überhaupt noch bewältigen kann, wenn niemand anderes wirklich hilft. Wie kann ich lernen, Hilfe anzunehmen, ohne das Gefühl zu haben, die Verantwortung zu verlieren?

Persona 5: Persona, die vorrangig aufgrund günstiger Umstände, traditioneller/kultureller Normen, familiärer Erwartungen und hierarchischer Strukturen die Pflege übernimmt

Story 5.5: Selbstverwirklichung und Eigenleben

[Einführung] Die letzten Jahre habe ich damit verbracht, mich nur um meine Schwiegermutter zu kümmern. Früher hatte ich Pläne, wollte reisen und neue Dinge ausprobieren, aber das hat alles keinen Platz mehr. Meine gesamte Zeit geht in die Pflege, und ich habe das Gefühl, dass ich mich selbst dabei verliere.

[Problembeschreibung] Es ist nicht nur die körperliche Belastung der Pflege, sondern auch das Fehlen von Erfüllung. Ich frage mich, was aus meinen eigenen Träumen und Zielen geworden ist. Ich habe nie die Chance, etwas nur für mich selbst zu tun. Ich fühle mich wie eine Schattenversion meiner selbst – immer nur in der Rolle der „Pflegenden".

[Abschluss] Es fällt mir schwer zu akzeptieren, dass mein Leben jetzt fast nur noch von der Pflege bestimmt wird. Aber das Pflichtgefühl gegenüber der Familie lässt mir wenig Raum, mich selbst zu verwirklichen. Wie kann ich Wege finden, wieder ein eigenes Leben zu führen, ohne das Gefühl zu haben, meine Familie im Stich zu lassen?

Persona 6: Persona, die eine alleinige, aktive, kontrollierende und entscheidende Pflegeperson ist und professionelle Unterstützung in Anspruch nimmt, weil sie glaubt, dass andere Familienmitglieder dazu nicht kompetent sind

Übersicht Storypaket

Story	Thema
Story 6.1	Einführung
Story 6.2	Missvertrauen in andere Familienmitglieder
Story 6.3	Kontrolle und Überforderung
Story 6.4	Das Bedürfnis nach Perfektion
Story 6.5	Professionelle Unterstützung

Persona 6: Persona, die eine alleinige, aktive, kontrollierende und entscheidende Pflegeperson ist und professionelle Unterstützung in Anspruch nimmt, weil sie glaubt, dass andere Familienmitglieder dazu nicht kompetent sind

Story 6.1: Einführung

[Einführung] Ich übernehme die Pflege meiner Mutter, weil ich der Meinung bin, dass niemand anders es richtig machen würde. Meine Geschwister sind entweder zu beschäftigt oder glauben, dass es keine große Sache ist. Sie sehen die Pflege meiner Mutter nicht so ernst wie ich. Deshalb habe ich mich entschieden, die Kontrolle zu übernehmen und alles selbst zu regeln. Ich stelle sicher, dass sie in den besten Händen ist, auch wenn das bedeutet, dass ich meine eigenen Bedürfnisse in den Hintergrund stelle.

[Problembeschreibung] Es fällt mir schwer, Aufgaben an andere Familienmitglieder zu delegieren. Ich bin mir manchmal nicht sicher, ob sie es richtig machen. Daher nehme ich auch professionelle Unterstützung in Anspruch. Aber selbst hier behalte ich die Kontrolle, weil ich sicherstellen möchte, dass alles gut läuft und die Pflege meiner Mutter sichergestellt ist.

[Abschluss] Es ist anstrengend, alles zu überwachen, und manchmal fühle ich mich erschöpft. Aber ich kann es einfach nicht lassen, weil ich fürchte, dass jemand anders die Verantwortung nicht genauso ernst nimmt und meine Mutter dann darunter leiden muss. Wie kann ich lernen, Verantwortung zu teilen, ohne das Gefühl zu haben, dass ich die Kontrolle verliere?

Persona 6: Persona, die eine alleinige, aktive, kontrollierende und entscheidende Pflegeperson ist und professionelle Unterstützung in Anspruch nimmt, weil sie glaubt, dass andere Familienmitglieder dazu nicht kompetent sind

Story 6.2: Missvertrauen in andere Familienmitglieder

[Einführung] Meine Geschwister sind immer bereit, mir zu helfen, aber ich kann mich einfach nicht darauf verlassen. Sie haben nie wirklich verstanden, was es bedeutet, sich um unsere Mutter zu kümmern. Und auch wenn sie es gut meinen, ich weiß, dass sie es nicht so gut und ausreichend tun würden, wie ich es machen würde.

[Problembeschreibung] Es fällt mir schwer, die Verantwortung abzugeben. Ich kann einfach nicht beruhigt sein, wenn ich nicht genau weiß, was gerade passiert. Sie könnten etwas übersehen oder falsch machen. Diese Gedanken machen mich unruhig und führen dazu, dass ich alles selbst mache – auch wenn es mich überfordert. Dann denke ich, dass ich einfach loslassen muss und Verantwortung abgeben sollte.

[Abschluss] Ich frage mich, wie lange ich so weitermachen kann. Vielleicht habe ich wirklich irgendwann keine Kraft mehr, aber die Vorstellung, dass jemand anders die Pflege übernimmt, macht mich nervös und unsicher. Wie kann ich lernen, anderen zu vertrauen und Verantwortung zu teilen, ohne ständig das Gefühl zu haben, dass ich die Kontrolle verliere?

Persona 6: Persona, die eine alleinige, aktive, kontrollierende und entscheidende Pflegeperson ist und professionelle Unterstützung in Anspruch nimmt, weil sie glaubt, dass andere Familienmitglieder dazu nicht kompetent sind

Story 6.3: Kontrolle und Überforderung

[Einführung] Es ist nicht einfach, alles allein zu regeln. Ich weiß, dass ich die Kontrolle über die Pflege behalten muss, um sicherzustellen, dass nichts schiefgeht. Aber gleichzeitig merke ich, wie sehr mich diese Verantwortung belastet. Es fühlt sich an, als ob ich ständig auf der Hut sein muss.

[Problembeschreibung] Trotz der Unterstützung durch professionelle Pflegekräfte habe ich das Gefühl, dass ich immer präsent sein muss. Ich überprüfe alles, was passiert, und stelle sicher, dass es nach meinen Vorstellungen läuft. Es ist anstrengend, diese Kontrolle zu bewahren, aber ich habe das Gefühl, dass niemand sonst es so gut machen kann. Doch je mehr ich versuche, alles zu steuern, desto mehr merke ich, wie mich das erschöpft.

[Abschluss] Es ist eine ständige Herausforderung, das Gleichgewicht zwischen Kontrolle und Überforderung zu finden. Manchmal frage ich mich, wie viel länger ich das durchhalten kann, ohne dass es mich völlig aufreibt. Wie kann ich lernen, ein gewisses Maß an Kontrolle abzugeben, ohne das Gefühl zu haben, dass die Pflege meiner Mutter darunter leidet?

Persona 6: Persona, die eine alleinige, aktive, kontrollierende und entscheidende Pflegeperson ist und professionelle Unterstützung in Anspruch nimmt, weil sie glaubt, dass andere Familienmitglieder dazu nicht kompetent sind

Story 6.4: Das Bedürfnis nach Perfektion

[Einführung] Ich stelle sicher, dass alles im besten Zustand für meine Mutter ist. Ich habe professionelle Hilfe eingestellt, aber ich kontrolliere alles, denn ich wurde schon mehrfach Zeuge davon, dass sie etwas vergessen haben. Ich möchte, dass meine Mutter in den besten Händen ist und die beste Unterstützung erhält. Daher fällt es mir schwer damit umzugehen, wenn jemand etwas nicht richtig macht oder eine andere Methode anwendet als die, die ich für richtig halte.

[Problembeschreibung] Ich merke, dass es immer schwieriger wird, alles perfekt zu machen. Vor allem, weil ich es nicht mehr schaffe, alles alleine zu machen. Ich muss einige Aufgaben abgeben, damit es meiner Mutter gut geht. Dabei habe ich aber das Gefühl, dass ich ständig gestresst bin. Ich fühle mich fast wie ein Wächter, der sicherstellt, dass alles in perfekter Ordnung bleibt.

[Abschluss] Ich frage mich, ob ich meiner Mutter die perfekte Pflege jemals geben kann oder ob ich mich irgendwann damit abfinden muss, dass es nicht immer perfekt laufen wird. Doch die Vorstellung, nachzulassen, macht mir Angst, denn ich möchte nicht, dass etwas schief läuft und meine Mutter die Konsequenzen dafür tragen muss. Wie kann ich lernen, die Pflege weniger streng zu kontrollieren, ohne das Gefühl zu haben, dass es zu Kompromissen führt?

Persona 6: Persona, die eine alleinige, aktive, kontrollierende und entscheidende Pflegeperson ist und professionelle Unterstützung in Anspruch nimmt, weil sie glaubt, dass andere Familienmitglieder dazu nicht kompetent sind

Story 6.5: Professionelle Unterstützung

[Einführung] Ich habe professionelle Pflegekräfte eingestellt, weil ich nicht mehr alles alleine stemmen kann. Das war schon ein sehr großer Schritt für mich und auch für meine Mutter. Damit alles gut geht, habe ich immer ein Auge auf die Betreuung. Ich spreche viel mit den Pflegekräften und bin die direkte Ansprechperson, wenn es etwas zu besprechen gibt.

[Problembeschreibung] Ich weiß, dass ich Hilfe brauche, aber es fällt mir schwer, diese Hilfe wirklich anzunehmen. Es fühlt sich einfach nicht richtig an, wenn jemand anders entscheidet, wie die Pflege durchgeführt wird. Ich will sicherstellen, dass sie genau das tun, was ich für meine Mutter für das Beste halte. Doch ich weiß natürlich auch, dass die Pflegekräfte die Experten in Sachen Pflege sind und ich das Zepter – zumindest ein bisschen – abgeben könnte.

[Abschluss] Es ist eine ständige Herausforderung, mit der Hilfe zurechtzukommen und gleichzeitig das Gefühl zu haben, alles selbst zu bestimmen. Ich frage mich, ob ich irgendwann wirklich loslassen und den professionellen Fachkräften vertrauen kann. Wie kann ich lernen, die Unterstützung zu nutzen, die mir angeboten wird, ohne mich dabei überflüssig oder kontrolliert zu fühlen?

Persona 7: Persona, die Pflegeentscheidungen rational und organisiert nach eigenem Ermessen, im Einklang mit ihrem eigenen Leben und mit professioneller Unterstützung trifft

Übersicht Storypaket

Story	Thema
Story 7.1	Einführung
Story 7.2	Pflege und Organisation
Story 7.3	Entscheidungen treffen
Story 7.4	Professionelle Unterstützung
Story 7.5	Emotionales Gleichgewicht finden

Persona 7: Persona, die Pflegeentscheidungen rational und organisiert nach eigenem Ermessen, im Einklang mit ihrem eigenen Leben und mit professioneller Unterstützung trifft

Story 7.1: Einführung

[Einführung] Ich bin eigentlich nicht die Hauptpflegeperson, aber ich kümmere mich darum, dass alles gut läuft bei der Pflege unserer Mutter. Weil ich berufstätig bin und eine eigene Familie habe, übernimmt die eigentliche Pflege meine Schwester und vor allem aber auch Pflegedienste, die ich engagiert habe. Ich versuche, die Verantwortung für die Organisation der Pflege zu übernehmen. Ich entscheide gemeinsam mit meiner Mutter, was sie braucht und kümmere mich darum, dass sie die richtige Hilfe bekommt. Ich habe auch professionelle Pflegekräfte eingestellt, die mich und meine Schwester unterstützen.

[Problembeschreibung] Es ist mir wichtig, dass alles gut organisiert ist, aber ich bin nicht so emotional in der Pflege. Ich weiß, dass es eine Aufgabe ist, die erledigt werden muss, und deshalb plane ich alles so, dass es passt. Ich sorge dafür, dass meine Mutter alles bekommt, was sie braucht, aber ich lasse nicht zu, dass die Pflege zu viel von meinen eigenen Bedürfnissen verlangt.

[Abschluss] Es funktioniert ganz gut so. Ich kann alles organisieren, ohne mich zu sehr mit den emotionalen Aspekten auseinanderzusetzen. Aber manchmal gibt mir meine Schwester das Gefühl, dass ich zu sehr „organisiere“ und die emotionalen Aspekte der Demenz meiner Mutter und ihren gesundheitlichen Zustand ignoriere. Wie kann ich sicherstellen, dass die Pflege gut organisiert ist, aber auch genug emotionale Unterstützung für meine Mutter und meine Schwester bietet?

Persona 7: Persona, die Pflegeentscheidungen rational und organisiert nach eigenem Ermessen, im Einklang mit ihrem eigenen Leben und mit professioneller Unterstützung trifft

Story 7.2: Pflege und Organisation

[Einführung] Ich sehe die Pflege als eine organisatorische Aufgabe. Ich sorge dafür, dass Termine eingehalten werden, die richtigen Leute für die Unterstützung da sind und dass alles, was für meine Mutter nötig ist, organisiert wird. Es geht mir darum, alles zu koordinieren, damit nichts vergessen wird. Meine Schwester sieht das ganz anders. Sie ist zu sehr emotional darin involviert.

[Problembeschreibung] Ich kümmere mich nicht um jede einzelne Aufgabe persönlich, aber ich sorge dafür, dass alles nach Plan läuft. Ich spreche regelmäßig mit den Pflegekräften und stelle sicher, dass alles gut funktioniert. Ich begleite meine Mutter auch zu ihren Arztterminen. Meine Schwester wirft mir manchmal vor, dass ich zu neutral mit der Situation umgehe und den emotionalen Teil der Pflege vernachlässige.

[Abschluss] Ich möchte, dass alles reibungslos läuft, aber vielleicht sollte ich mehr auf die Bedürfnisse meiner Mutter hören und mich weniger auf das Organisatorische konzentrieren. Wie finde ich einen besseren Ausgleich zwischen der Organisation der Pflege und der emotionalen Unterstützung für meine Mutter?

Persona 7: Persona, die Pflegeentscheidungen rational und organisiert nach eigenem Ermessen, im Einklang mit ihrem eigenen Leben und mit professioneller Unterstützung trifft

Story 7.3: Entscheidungen treffen

[Einführung] Ich treffe die Entscheidungen für die Pflege meiner Mutter, aber ohne dass es mich emotional belastet. Ich beziehe die Familie und die Pflegekräfte ein, aber ich bin diejenige Person, die die Richtung vorgibt. Es fühlt sich gut an, weil ich die Kontrolle habe, aber manchmal frage ich mich, ob ich genug mit meiner Mutter spreche, um herauszufinden, wie sie sich fühlt.

[Problembeschreibung] Es ist wichtig, dass alles funktioniert, aber ich denke nicht ständig an die emotionalen Aspekte. Ich entscheide pragmatisch, was für sie am besten ist. Aber durch diese Haltung könnte ich vielleicht etwas übersehen, was für meine Mutter wichtig ist, auch wenn es nicht direkt mit der Pflege zu tun hat.

[Abschluss] Ich weiß, dass ich gut organisieren kann, aber ich frage mich, ob ich beim Treffen von Entscheidungen in der Pflege nicht mehr auf die emotionalen Bedürfnisse meiner Mutter eingehen sollte und ob ich dabei auch meine Schwester mehr einbeziehen sollte, die sehr viel Zeit mit meiner Mutter verbringt. Wie kann ich besser erkennen, wenn meine Mutter emotionale Unterstützung braucht, ohne die praktische Organisation aus den Augen zu verlieren?

Persona 7: Persona, die Pflegeentscheidungen rational und organisiert nach eigenem Ermessen, im Einklang mit ihrem eigenen Leben und mit professioneller Unterstützung trifft

Story 7.4: Professionelle Unterstützung

[Einführung] Ich habe professionelle Pflegekräfte eingestellt, die sich um die täglichen Aufgaben kümmern. Sie sind gut ausgebildet und können meiner Mutter helfen, wo sie es braucht. Ich stehe immer zur Verfügung, wenn es um Entscheidungen geht, aber ich lasse die Pflegekräfte die praktische Arbeit übernehmen. Meine Schwester entlaste ich dadurch auch.

[Problembeschreibung] Ich kümmere mich um die finanziellen und organisatorischen Aspekte und lasse die Pflegekräfte die pflegerische Arbeit erledigen. Es läuft gut, aber manchmal fühle ich mich nicht so eingebunden, wenn es um die alltägliche Pflege geht.

[Abschluss] Ich möchte sicherstellen, dass meine Mutter die bestmögliche Hilfe bekommt. Aber irgendwie fühle ich mich manchmal abgekoppelt von der emotionalen Verbindung, die andere Pflegepersonen oder auch meine Schwester haben. Wie kann ich mich mehr in die tägliche Pflege einbringen, ohne die Verantwortung für alles zu übernehmen?

Persona 7: Persona, die Pflegeentscheidungen rational und organisiert nach eigenem Ermessen, im Einklang mit ihrem eigenen Leben und mit professioneller Unterstützung trifft

Story 7.5: Emotionales Gleichgewicht finden

[Einführung] Es fällt mir nicht leicht, bei der Pflege meiner Mutter emotional zu werden. Ich organisiere alles, aber wenn es um Gefühle geht, bin ich nicht so sehr involviert. Ich sorge dafür, dass sie in guten Händen ist und die richtige Unterstützung bekommt, aber manchmal frage ich mich, ob ich genug für ihre emotionalen Bedürfnisse tue und ob es normal ist, dass es mich so kalt lässt, dass meine Mutter krank ist und Pflege braucht.

[Problembeschreibung] Ich merke, dass ich manchmal die Distanz bewahre, wenn es um die emotionalen Aspekte geht. Vielleicht, weil ich mich nicht mit meinen Ängsten und Sorgen befassen möchte. Ich kümmere mich um die Finanzen und den Plan, aber die Gespräche über ihre Gefühle oder Wünsche führe ich nicht oft. Ich will sicherstellen, dass sie gut versorgt ist, aber ich frage mich, ob ich zu wenig auf das höre, was sie wirklich braucht.

[Abschluss] Vielleicht sollte ich mir mehr Zeit nehmen, um wirklich auf ihre emotionalen Bedürfnisse einzugehen, auch wenn es nicht meine Stärke ist. Wie kann ich den emotionalen Teil der Pflege besser in meinen Alltag integrieren, ohne meine organisatorische Rolle zu vernachlässigen?

Persona 8: Persona, die sich aktiv aus der Pflegesituation zurückzieht und eine maximal unterstützende, eher beobachtende Rolle einnimmt

Übersicht Storypaket

Story	Thema
Story 8.1	Einführung
Story 8.2	Unterstützung in der Pflege
Story 8.3	Abgrenzung von der Pflege
Story 8.4	Familie und Unterstützung
Story 8.5	Rolle in der Pflege

Persona 8: Persona, die sich aktiv aus der Pflegesituation zurückzieht und eine maximal unterstützende, eher beobachtende Rolle einnimmt

Story 8.1: Einführung

[Einführung] Ich helfe, wo ich kann, aber eigentlich stehe ich eher draußen. Die Pflege meiner Mutter ist vor allem die Aufgabe meiner Schwester. Ich unterstütze sie, wenn es nötig ist, aber ich bin nicht wirklich in den Alltag der Pflege eingebunden. Ich kümmere mich mehr um andere Dinge und überlasse die Pflege meiner Schwester.

[Problembeschreibung] Ich bin zwar da, wenn sie Hilfe braucht, aber ich fühle mich nicht so verantwortlich für die Pflege. Es gibt immer Aufgaben, die ich übernehme, wie Einkaufen oder Kochen, aber die Pflege selbst, das ist nicht wirklich mein Ding. Ich bin mir nicht sicher, wie ich mich anders einbringen könnte, ohne mich zu sehr zu involvieren.

[Abschluss] Ich frage mich, ob ich nicht mehr tun sollte, aber irgendwie fühle ich mich nicht wirklich zuständig. Ich versuche, die Pflege zu unterstützen, aber nicht aktiv teilzunehmen. Wie kann ich meine Rolle in der Pflege verändern, ohne mich überfordert zu fühlen?

Persona 8: Persona, die sich aktiv aus der Pflegesituation zurückzieht und eine maximal unterstützende, eher beobachtende Rolle einnimmt

Story 8.2: Unterstützung in der Pflege

[Einführung] Ich bin hin und wieder da, wenn Hilfe gebraucht wird, aber ehrlich gesagt weiß ich nicht viel über die Pflege. Meine Schwester übernimmt fast alles. Ich kümmere mich um die praktische Unterstützung, wie das Besorgen von Lebensmitteln oder das Organisieren von Arztterminen. Aber über die Pflege selbst habe ich nicht viel Ahnung.

[Problembeschreibung] Ich fühle mich oft unsicher, was die richtige Hilfe ist. Ich frage mich, ob ich genug tue oder ob ich mich mehr einbringen sollte. Aber irgendwie fehlt mir das Wissen, um wirklich aktiv zu helfen. Es ist schwer für mich, zu verstehen, was meine Mutter braucht, vor allem, wenn es um die Pflege selbst geht.

[Abschluss] Ich möchte mehr verstehen, was meine Mutter braucht, aber ich weiß nicht, wo ich anfangen soll. Vielleicht sollte ich mich mehr informieren. Wie kann ich mehr über die Pflege lernen, ohne mich zu überfordern?

Persona 8: Persona, die sich aktiv aus der Pflegesituation zurückzieht und eine maximal unterstützende, eher beobachtende Rolle einnimmt

Story 8.3: Abgrenzung von der Pflege

[Einführung] Ich habe die Pflege nicht übernommen, weil ich das Gefühl habe, dass es besser ist, wenn meine Schwester sich darum kümmert. Sie hat mehr Erfahrung, und ich möchte mich nicht in eine Rolle drängen, die ich nicht will. Ich helfe bei den Aufgaben, die ich mir zutraue, aber ansonsten halte ich mich aus der Pflege heraus.

[Problembeschreibung] Es fällt mir schwer, mich richtig einzubringen, weil ich nicht so viel Verantwortung übernehmen möchte. Ich fühle mich nicht wohl dabei, täglich in die Pflege involviert zu sein. Vielleicht bin ich deshalb manchmal frustriert, dass die Pflege auf nur wenigen Schultern lastet, aber ich möchte nicht mehr tun, als ich gerade tue.

[Abschluss] Es ist eine schwierige Situation, weil ich mich nicht als Hauptpflegeperson sehe. Aber irgendwie fühle ich mich auch schuldig, dass ich nicht mehr tue. Wie kann ich mich mehr einbringen, ohne meine Grenzen zu überschreiten?

Persona 8: Persona, die sich aktiv aus der Pflegesituation zurückzieht und eine maximal unterstützende, eher beobachtende Rolle einnimmt

Story 8.4: Familie und Unterstützung

[Einführung] Ich helfe, wo es nötig ist, aber meine Familie kümmert sich hauptsächlich um die Pflege. Ich bin da, wenn sie mich brauchen, aber ich fühle mich nicht verantwortlich für alles. Es gibt immer wieder Situationen, in denen ich gefragt werde, etwas zu übernehmen, aber es fällt mir schwer, wirklich aktiv zu werden. Dabei geht es ja auch um meine Mutter, die krank ist.

[Problembeschreibung] Ich denke oft, dass andere Familienmitglieder mehr tun könnten, aber ich habe das Gefühl, dass ich nicht die Person bin, die ständig in die Pflege eingreifen sollte. Ich möchte zwar unterstützen, aber ich merke, dass ich mich emotional abgrenze. Es fällt mir schwer, mich in die Pflege voll einzubringen, und ich habe oft das Gefühl, dass meine Familie das untereinander besser regeln kann. Andererseits möchte ich meine Familie auch unterstützen und entlasten und ich möchte nicht, dass es so wirkt, als wäre mir die Situation egal. Aber eigentlich bin ich auch zufrieden mit der Situation.

[Abschluss] Ich bin da, wenn ich gebraucht werde, aber irgendwie habe ich das Gefühl, dass ich nicht wirklich viel verändern kann, wenn es um die Pflege meiner Mutter geht. Dabei möchte ich helfen. Wie kann ich helfen, ohne mich zu sehr in die Pflege einzugreifen?

Persona 8: Persona, die sich aktiv aus der Pflegesituation zurückzieht und eine maximal unterstützende, eher beobachtende Rolle einnimmt

Story 8.5: Emotionales Gleichgewicht finden

[Einführung] Ich sehe die Pflege meiner Mutter eher aus der strukturellen Perspektive. Es gibt bestimmte Aufgaben, die erledigt werden müssen, und ich bin da, um diese Aufgaben zu unterstützen. Aber wenn es darum geht, wie es meiner Mutter geht oder wie sich alle fühlen, bin ich eher distanziert. Ich tue, was ich tun muss, aber mehr nicht.

[Problembeschreibung] Es gibt Momente, in denen ich merke, dass ich mich aus der Pflege herausnehme, weil ich nicht so sehr mit den Gefühlen oder dem emotionalen Aspekt der Pflege beschäftigt sein möchte. Ich kümmere mich um organisatorische Dinge, wie die medizinische Versorgung, aber die emotionalen Bedürfnisse meiner Mutter überlasse ich anderen.

[Abschluss] Es fällt mir schwer, mich mit der emotionalen Seite der Pflege auseinanderzusetzen. Ich mache, was notwendig ist, aber es fühlt sich nicht so an, als ob ich wirklich aktiv in die Pflege eingebunden bin. Wie kann ich meine Rolle in der Pflege klarer definieren und mich gleichzeitig nicht überfordert fühlen?

Persona 9: Persona, die über Demenz und Pflege informiert ist und daher nur Fachleuten vertraut und sich selbst als engagierten Wissensvermittler sieht

Übersicht Storypaket

Story	Thema
Story 9.1	Einführung
Story 9.2	Vertrauen in Fachleute
Story 9.3	Überlastung durch Verantwortung
Story 9.4	Kulturelle Erwartungen und Pflege
Story 9.5	Wissen und Unterstützung

Persona 9: Persona, die über Demenz und Pflege informiert ist und daher nur Fachleuten vertraut und sich selbst als engagierten Wissensvermittler sieht

Story 9.1: Einführung

[Einführung] Ich bin die Hauptpflegeperson für meine Mutter, die an Demenz erkrankt ist. Ich habe mich intensiv mit der Krankheit und der Pflege beschäftigt, weil ich sicherstellen möchte, dass sie die beste Versorgung bekommt. Ich vertraue nur professionellen Fachleuten, wenn es um ihre Pflege geht – Ärzte, Pflegekräfte und spezialisierte Beratungsstellen.

[Problembeschreibung] Es fällt mir schwer, anderen Familienmitgliedern zu vertrauen, wenn es um die Pflege geht. Ich weiß viel über Demenz und Pflege, aber oft habe ich das Gefühl, dass andere nicht genug wissen. Manchmal bekomme ich von meiner Familie Ratschläge, aber ich bin mir nicht sicher, ob sie wirklich verstehen, was meine Mutter braucht. Ich versuche, ihre Bedürfnisse so gut wie möglich zu erfüllen, aber es ist schwer, alles zu managen.

[Abschluss] Ich merke, dass es mich überfordert, alles alleine zu regeln. Aber ich vertraue niemandem so richtig, auch wenn ich mir Hilfe von außen hole. Ich möchte die Kontrolle behalten und sicherstellen, dass alles richtig gemacht wird. Wie kann ich lernen, mehr Vertrauen in andere zu setzen, ohne dabei die Kontrolle zu verlieren?

Persona 9: Persona, die über Demenz und Pflege informiert ist und daher nur Fachleuten vertraut und sich selbst als engagierten Wissensvermittler sieht

Story 9.2: Vertrauen in Fachleute

[Einführung] Ich nehme regelmäßig Kontakt zu Ärzten, Pflegekräften und Demenzberatungsstellen auf, um sicherzustellen, dass wir immer die besten Informationen und Unterstützung bekommen. In meiner Familie bin ich diejenige, die über die Pflege entscheidet, weil ich die meiste Erfahrung und das Wissen habe. Die anderen Familienangehörigen wissen das auch und vertrauen mir deshalb. Sie geben mir gerne die Verantwortung für die Pflege ab.

[Problembeschreibung] Andererseits verstehen sie dann aber oft nicht, warum ich so auf professionelle Hilfe bestehe. Sie denken, ich könnte die Pflege auch ohne externe Unterstützung schaffen. Aber ich weiß, dass Demenz eine Krankheit ist, bei der Fachleute sehr wichtig sind. Es gibt viele Dinge, die ich alleine nicht weiß, und ich möchte nicht riskieren, dass meine Mutter falsche Behandlung bekommt.

[Abschluss] Es ist manchmal schwierig, meine Familie davon zu überzeugen, dass professionelle Hilfe notwendig ist. Aber ich bleibe bei meiner Meinung, dass Fachleute mehr Wissen haben und besser wissen, was für meine Mutter wichtig ist. Wie kann ich meiner Familie besser erklären, warum ich so auf professionelle Hilfe bestehe?

Persona 9: Persona, die über Demenz und Pflege informiert ist und daher nur Fachleuten vertraut und sich selbst als engagierten Wissensvermittler sieht

Story 9.3: Überlastung durch Verantwortung

[Einführung] Da ich die Hauptpflegeperson für meine Mutter bin, trage ich die meiste Verantwortung. Ich organisiere alles – vom Arztbesuch bis hin zu den täglichen Pflegeaufgaben. Manchmal fühlt es sich so an, als würde alles nur an mir hängen. Es ist schwer, das alles zu managen, besonders mit meiner eigenen Familie und Arbeit.

[Problembeschreibung] Ich habe oft das Gefühl, dass ich in allem die Kontrolle behalten muss, um sicherzustellen, dass alles richtig läuft. Doch es gibt auch Momente, in denen ich überfordert bin. Es fällt mir schwer, eine Balance zwischen meiner Pflegeaufgabe und meinem eigenen Leben zu finden. Ich möchte meiner Mutter alles geben, aber es bleibt kaum Zeit für mich.

[Abschluss] Es ist eine ständige Herausforderung, alles unter einen Hut zu bekommen. Aber ich bin fest entschlossen, meine Mutter bestmöglich zu betreuen, auch wenn es anstrengend ist. Wie kann ich die Pflege besser organisieren, ohne mich selbst zu verlieren?

Persona 9: Persona, die über Demenz und Pflege informiert ist und daher nur Fachleuten vertraut und sich selbst als engagierten Wissensvermittler sieht

Story 9.4: Kulturelle Erwartungen und Pflege

[Einführung] In meiner Familie gibt es eine starke Tradition, sich um die älteren Generationen zu kümmern. Ich verstehe diese Tradition, aber gleichzeitig habe ich festgestellt, dass Demenz eine ganz andere Art der Pflege erfordert, die nicht jeder versteht. Ich achte darauf, die kulturellen Erwartungen zu wahren, aber ich weiß, dass die Pflege meiner Mutter auf einer anderen Ebene stattfinden muss.

[Problembeschreibung] Es gibt immer wieder Situationen, in denen meine Familie erwartet, dass ich meine Mutter auf eine bestimmte Weise pflege. Aber ich weiß, dass das nicht immer die beste Lösung ist. Zum Beispiel werden manche traditionelle Pflegerituale nicht mehr funktionieren, wenn meine Mutter nicht mehr in der Lage ist, sich selbst zu helfen. Ich versuche, diese Balance zu finden – einerseits die Tradition zu respektieren, andererseits aber die richtige Pflege sicherzustellen.

[Abschluss] Es ist schwierig, beide Welten miteinander zu verbinden – die traditionellen Werte meiner Familie und das, was meine Mutter wirklich braucht. Aber ich versuche, das Beste aus beiden Aspekten zu machen. Wie kann ich die kulturellen Erwartungen der Familie mit den Bedürfnissen meiner Mutter in Einklang bringen?

Persona 9: Persona, die über Demenz und Pflege informiert ist und daher nur Fachleuten vertraut und sich selbst als engagierten Wissensvermittler sieht

Story 9.5: Wissen und Unterstützung

[Einführung] Da ich so viel über Demenz und Pflege weiß, teile ich dieses Wissen auch gerne mit anderen. Ich finde es wichtig, dass alle verstehen, was meine Mutter braucht und wie sie richtig gepflegt wird. Aber manchmal fällt es mir schwer, mir selbst die Hilfe zu holen, die ich brauche, obwohl ich weiß, dass Unterstützung von außen sehr hilfreich sein könnte.

[Problembeschreibung] Ich rede viel mit anderen über Demenz, aber es fühlt sich an, als würde niemand wirklich verstehen, wie schwierig es ist. Obwohl ich viel weiß, kann ich nicht alles alleine machen. Aber ich habe auch Schwierigkeiten, die Hilfe anzunehmen, die mir angeboten wird, da ich mir nicht sicher bin, ob die anderen Personen auch über so viel Wissen verfügen.

[Abschluss] Es ist ein ständiger Balanceakt zwischen dem Teilen von Wissen und dem Akzeptieren von Hilfe. Aber ich weiß, dass ich Unterstützung brauche, auch wenn es mir schwerfällt, diese anzunehmen. Vielleicht müsste ich mein Wissen mehr teilen und könnte dann auch Unterstützung annehmen, wenn ich weiß, die anderen Personen sind auf meinem Wissensstand. Wie kann ich lernen, mein Wissen zu teilen und dann auch Unterstützung anzunehmen, die mich entlasten kann?
