# Supplementary material for: Storytelling in family-provided care - developing diversity-sensitive stories for family caregivers of Turkish individuals living with dementia
Source: BMC Nurs. 2025 Aug 12;24:1060. doi: 10.1186/s12912-025-03712-7 (PMC12341143; doi:10.1186/s12912-025-03712-7)
Supplement: Supplementary file 3 — Supplementary Material 3 [file 12912_2025_3712_MOESM3_ESM.pdf]

Persona 1: Rol çatışması yaşayan ve birden fazla baskı altında olan bir kişi, iş hayatının gerekliliklerini, hasta bir aile üyesine bakmayı ve kendi ailesini ayakta tutmayı dengelemek zorunda kalan bir kişi

Hikaye paketine genel bakış

Hikaye	Konu
Hikaye 1.1	Giriş
Hikaye 1.2	Stresle Başa Çıkma ve Zaman Yönetimi
Hikaye 1.3	Aile Desteği ve Çatışma Çözme
Hikaye 1.4	Profesyonel Destek Hizmetlerine Erişim ve Kullanım
Hikaye 1.5	Farklı Roller Arasında Denge Bulma

Persona 1: Rol çatışması yaşayan ve birden fazla baskı altında olan bir kişi, iş hayatının gerekliliklerini, hasta bir aile üyesine bakmayı ve kendi ailesini ayakta tutmayı dengelemek zorunda kalan bir kişi

Hikaye 1.1: Giriş

[Giriş] Anneme tek başıma bakıyorum. Bir fırında tam zamanlı çalışıyorum ve iki kızım var. Bazen her şeyi idare etmek benim için çok zor oluyor. Kardeşlerimden gerçekten hiç yardım almıyorum. Ailedeki diğer kişiler annemizin hasta olduğuna inanmıyor. “Annem normal görünüyor.” ya da “Abartıyorsun.” diyorlar.

[Sorun tanımı] Her şeyi bağdaştırmak benim için çok zor. Kimseye haksızlık ediyormuşum gibi bir his var içimde. Ben de uzun zamandır kendime haksızlık ediyorum. Her şeyi idare etmek için yardıma ihtiyacım olduğunu biliyorum. Ama çok az zamanım var. Bir mola programından nasıl faydalanabilirim ki? Anneme bakmak için zaman bulabilirsem ne mutlu bana.

[Sonuç] Bazen sadece kapanmak ya da duraklatma düğmesine basmak istiyorum. Rahatlayabildiğim ve annemin hastalığı hakkında düşünmek zorunda kalmadığım zamanlar benim için tatil gibi oluyor. Bu kadar stresli olmam her zaman kocamla ya da kızlarımla tartışmama neden oluyor. Bu benim için çok stresli. Aynı anda nasıl bakıcı, eş, anne ve çalışan olacağımı bilemiyorum. Sürekli baskı altında kalmamak ve az zamanım olmasına rağmen kendimi rahatlatmak için ne yapabilirim?

Persona 1: Rol çatışması yaşayan ve birden fazla baskı altında olan bir kişi, iş hayatının gerekliliklerini, hasta bir aile üyesine bakmayı ve kendi ailesini ayakta tutmayı dengelemek zorunda kalan bir kişi

Story 1.2: Stresle Başa Çıkma ve Zaman Yönetimi

[Giriş] Bazen günümde yeterince saat olmadığını hissediyorum. Fırında çalışmam, anneme bakmam ve kızlarıma annelik yapmam arasında kendime neredeyse hiç zaman ayıramıyorum. Çoğu zaman daha sabah kalkar kalkmaz yapılacaklar listemi kafamdan geçirmeye başlıyorum ve gün tam olarak başlamadan önce bile stres hissediyorum.

[Sorun Tanımı] Stresin sadece beni değil, çevremdekileri de etkilediğini fark ediyorum. Daha çabuk sinirleniyorum ve kızlarımla ya da eşimle vakit geçirecek enerjim kalmıyor. Bir iş arkadaşım bir keresinde bana "önceliklerini belirlemeyi öğrenmelisin" demişti ama bunu nasıl yapacağımı bilmiyorum. Her şey önemli görünüyor – bakım, ev işleri, iş... Hangisinin bekleyebileceğine nasıl karar verebilirim?

[Sonuç] Geçenlerde zaman yönetimiyle ilgili bir kitap okudum. Bu kitap, belirli görevler için sabit zaman dilimleri ayırma ve kendime bilinçli olarak küçük molalar verme fikrini bana aşıladı. Bunun benim için işe yarayıp yaramayacağını merak ediyorum. Günlük hayatımı nasıl daha iyi organize edebilirim ki daha az stres yaşayayım ama yine de önemli olan her şeyi yapabileyim?

Persona 1: Rol çatışması yaşayan ve birden fazla baskı altında olan bir kişi, iş hayatının gerekliliklerini, hasta bir aile üyesine bakmayı ve kendi ailesini ayakta tutmayı dengelemek zorunda kalan bir kişi

Hikaye 1.3: Aile Desteği ve Çatışma Çözme

[Giriş] Hâlâ annemle ilgilenen tek kişi benim. Kardeşlerim, onun "o kadar da hasta görünmediğini" düşünüyor. Geçenlerde abim ile yine bir tartışma yaşadım. Ondan en azından arada bir gelip annemi birkaç saatliğine ziyaret etmesini istedim ki biraz kendime zaman ayırabileyim.

[Sorun Tanımı] Verdiği cevap beni çok sinirlendirdi: "Sen zaten iyi yapıyorsun, neden ben karışayım ki?" Kendimi tamamen yalnız hissediyorum. Ailem, bunun benim için ne kadar yorucu olduğunu anlamadığı için üzülüyorum. Aynı zamanda, bu tür çatışmaların aile içindeki ilişkilerimizi kalıcı olarak bozmasından korkuyorum.

[Sonuç] Bu tartışmadan sonra, kardeşlerimi nasıl daha fazla destek olmaya teşvik edebileceğimi düşündüm—hem de her seferinde kavga etmeden. Belki de onlara gerçekten nasıl hissettiğimi ve neye ihtiyacım olduğunu daha net anlatmam gerekir. Aileme içinde bulunduğum durumu nasıl anlatabilirim ki sürekli tartışmalar yaşanmadan beni anlayabilsinler?

Persona 1: Rol çatışması yaşayan ve birden fazla baskı altında olan bir kişi, iş hayatının gerekliliklerini, hasta bir aile üyesine bakmayı ve kendi ailesini ayakta tutmayı dengelemek zorunda kalan bir kişi

Story 1.4: Profesyonel Destek Hizmetlerine Erişim ve Kullanım

[Giriş] Geçen hafta işyerinde bir iş arkadaşımla konuştum, bana yakınlardaki bir bakım hizmetinden bahsetti. Kayınvalidesi için oradan destek aldıklarını söyledi. Bunun bizim için de bir çözüm olup olmayacağını düşündüm, ama bunu günlük hayatıma nasıl entegre edebileceğimi bilemiyorum.

[Sorun Tanımı] Aslında acilen bir rahatlamaya ihtiyacım var, ama bunun için zamanım olmadığını hissediyorum. Anneme bakmak zaten yeterince zor, bir de bakım hizmetiyle görüşme ayarlamak bana imkânsız görünüyor. Ayrıca, bu hizmetin bize uygun olup olmayacağı konusunda endişelerim var. Duyduğuma göre, birçok bakıcı Türkçe konuşmuyor. Annem ise Almanca'yı çok az anlıyor; bu durumda onlarla nasıl iletişim kuracak? Bunun yanı sıra, annemin hassas olduğu konuların göz ardı edilmesinden korkuyorum—örneğin, domuz eti yememesi gibi. Annem de bana "eve yabancı birini almak istemediğini" söyledi. Ona bu desteğe ihtiyacımız olduğunu nasıl anlatacağımı bilmiyorum.

[Sonuç] Böyle bir bakım hizmetinin gerçekten yardımcı olup olmayacağını ve annemi en azından denemesi için nasıl ikna edebileceğimi düşünüyorum. Belki de önce, bakım personelinin dil ve beslenme gibi konulara özen gösterip göstermediğini sormalıyım. Hatta belki Türkçe konuşan bakım görevlileri bile vardır. Bizim ihtiyaçlarımızı anlayabilecek bir bakım hizmetini nasıl bulabilirim ve annemi bunu denemeye nasıl ikna edebilirim?

Persona 1: Rol çatışması yaşayan ve birden fazla baskı altında olan bir kişi, iş hayatının gerekliliklerini, hasta bir aile üyesine bakmayı ve kendi ailesini ayakta tutmayı dengelemek zorunda kalan bir kişi

Story 1.5: Farklı Roller Arasında Denge Bulma

[Giriş] Bazen tüm rollerimi nasıl hakkıyla yerine getirebileceğimi düşünüyorum. Bakıcıyım, anneyim, eşim, çalışan bir kadınım – ama peki ya ben? Geçenlerde kızım bana neden onunla bu kadar az vakit geçirdiğimi sordu. Bunu duymak beni derinden etkiledi.

[Sorun Tanımı] Sürekli herkesten gelen beklentileri karşılamaya çalıştığımı fark ediyorum, ama bu süreçte kendimi kaybediyorum. Kızlarımla daha fazla vakit geçirirsem annemin bakımı aksıyor. Bakıma odaklanırsam eşimle olan ilişkim zarar görüyor. Sanki ne yaparsam yapayım kimseyi mutlu edemiyorum.

[Sonuç] Ne istediğimi ve önceliklerimi nasıl belirlemem gerektiğini düşünmeye başladım. Belki de sınırlarımı daha net ifade etmeyi ve daha fazla destek istemeyi öğrenmem gerekiyor, ama bunu yapmak benim için kolay değil. Farklı rollerimi daha iyi dengeleyip aynı zamanda kendime de zaman ayırmayı nasıl başarabilirim?

Persona 2: Kendini yalnız, bunalmış ve aile çatışmaları ve aile üyesinin hastalığının kaderi tarafından yüklenmiş hisseden bir kişi

Hikaye paketine genel bakış

Hikaye	Konu
Hikaye 2.1	Giriş
Hikaye 2.2	Yalnızlıkla Başa Çıkma
Hikaye 2.3	Aşırı Bakım Yükü
Hikaye 2.4	Dini Destek ve İçsel Güç
Hikaye 2.5	Kendi Kişiliğini Kaybetmesi

LVF Lehrstuhl für Versorgungsforschung Universität Witten/Herdecke
ASH Berlin Alice Salomon Hochschule Berlin University of Applied Sciences
Demenz Support Stuttgart Zentrum für Informationstransfer

Persona 2: Kendini yalnız, bunalmış ve aile çatışmaları ve aile üyesinin hastalığının kaderi tarafından yüklenmiş hisseden bir kişi

Hikaye 2.1: Giriş

[Giriş] Annem üç yıl önce vefat ettiğinden beri babama tek başıma bakıyorum. Bana yardım edebilecek kimse yok ve bakımı Tanrı'ya karşı bir görevim olarak görüyorum. Babam bana hayat verdi, şimdi ise benim görevim ona bakmak. Ancak, bu sorumluluğun omuzlarıma gittikçe daha fazla ağırlık yaptığını hissediyorum.

[Sorun Tanımı] Artık ailem dışında insanlarla doğru düzgün bir iletişimim de yok ve günlerim hep aynı geçiyor. Uyanıyorum, babamla ilgileniyorum, ev işleri yapıyorum ve gereken her şeyi hallediyorum. Kendime ayıracak zamanım kalmıyor ve bazen, "Bakıcılık yapan kişi olmaktan başka kimim?" diye düşünüyorum. Her gün dua ediyorum, bu yükü taşımaya devam edebilmek için güç bulmayı diliyorum, ama çoğu zaman kendimi bunalmış ve yalnız hissediyorum.

[Sonuç] Bazen konuşabileceğim, her şeyi tek başıma taşımamın ne kadar zor olduğunu anlayan birinin olmasını diliyorum. Ama duygularımı içimde tutmayı öğrendim, çünkü şikâyet etmek doğru olmaz. Sorumluluğumu veya değerlerimi ihmal etmeden, içinde bulunduğum durumda nasıl bir rahatlama bulabilirim?

Persona 2: Kendini yalnız, bunalmış ve aile çatışmaları ve aile üyesinin hastalığının kaderi tarafından yüklenmiş hisseden bir kişi

Story 2.2: Yalnızlıkla Başa Çıkma

[Giriş] Bazen etrafımın ne kadar sessiz olduğunu fark ediyorum. Babam artık neredeyse hiç konuşmuyor ve kendimi ifade edebileceğim kimsem yok. Eskiden arkadaşlarım vardı, ama sürekli evde olduğum için onlarla olan bağımı kaybettim.

[Sorun Tanımı] Yalnızlığın bana iyi gelmediğini biliyorum, ama bunu değiştirmek konusunda kendimi çaresiz hissediyorum. Bazen sadece biriyle sohbet etmeyi ya da benimle benzer bir durum yaşayan birini tanımayı hayal ediyorum. Ancak, bakımı aksatmadan bunu nasıl yapabileceğimi bilmiyorum.

[Sonuç] İçinde bulunduğum duruma rağmen yeniden insanlarla bağlantı kurmanın bir yolu olup olmadığını merak ediyorum. Belki de bu, kendimi daha az yalnız hissetmeme yardımcı olur. Görevlerimi ihmal etmeden yalnızlığımı nasıl aşabilirim?

Persona 2: Kendini yalnız, bunalmış ve aile çatışmaları ve aile üyesinin hastalığının kaderi tarafından yüklenmiş hisseden bir kişi

Hikaye 2.3: Aşırı Bakım Yükü

[Giriş] Geçenlerde babam gece acı çektiği için beni çağırdı. Ona yardımcı oldum, ama sonrasında bir daha uyuyamadım. Böyle geceler gittikçe daha sık yaşanıyor ve enerjimin tükendiğini hissediyorum.

[Sorun Tanımı] Bakım her geçen gün daha yorucu hale geliyor ve bununla nasıl başa çıkacağımı bazen bilemiyorum. Öyle anlar oluyor ki, her şeyin üzerime geldiğini hissediyorum. Zaten elimden gelen her şeyi yaparken, yeterince çaba göstermediğimden endişe ediyorum.

[Sonuç] Bazen, bakımı daha iyi nasıl yönetebileceğimi ve bunu yaparken kendimi nasıl koruyabileceğimi gösterecek birinin olup olmadığını merak ediyorum. Ama kimseyi tanımıyorum ve nasıl daha iyi olabileceğini de bilmiyorum. Hem babamın bakımını en iyi şekilde sağlamak hem de tükenmişlik hissiyle nasıl başa çıkabilirim?

Persona 2: Kendini yalnız, bunalmış ve aile çatışmaları ve aile üyesinin hastalığının kaderi tarafından yüklenmiş hisseden bir kişi

Story 2.4: Dini Destek ve İçsel Güç

[Giriş] İnancımda teselli buluyorum. Her gün Allah'tan bana babama bakma gücü vermesini diliyorum. Bakımı, hem bir görevim hem de inancımı yaşamanın bir yolu olarak görüyorum.

[Sorun Tanımı] Ancak, inancıma rağmen bazen şüpheye kapılıyorum. Öyle günler oluyor ki, yeterince çaba gösterip göstermediğimi ya da babama gerçekten hakkını verip vermediğimi sorguluyorum. Bu düşünceler aklımdan çıkmıyor ve gücümün tükendiğini hissettiğimde suçluluk duyuyorum.

[Sonuç] İnancımı daha da güçlendirerek bakımın getirdiği zorluklarla nasıl baş edebileceğimi merak ediyorum. Belki de sorumluluklarımı daha fazla iç huzurla yerine getirmenin yolları vardır. Zor zamanlarda inancımın bana daha fazla güç vermesi için ne yapabilirim?

Persona 2: Kendini yalnız, bunalmış ve aile çatışmaları ve aile üyesinin hastalığının kaderi tarafından yüklenmiş hisseden bir kişi

Story 2.5: Kendi Kişiliğini Kaybetmesi

[Giriş] Babama bakmaya başladığımdan beri hayatımı tamamen değiştirdim. Aslında bunu bilinçli olarak yapmadım. Eskiden hobilerim ve hayallerim vardı, ama şimdi her şey bakımla ilgili. Arkadaşlarımla vakit geçiremiyorum ve yeni şeyler denemek için isteğim bile yok. Bakıcı rolümün dışında kim olduğumu artık bilmiyorum.

[Sorun Tanımı] Kendimi kaybetmiş gibi hissediyorum. Eskiden olduğum kişiyi özlüyorum, ama sorumluluklarımı aksatmadan ona nasıl geri dönebileceğimi bilmiyorum. Bazen kendime hiç vakit ayıramayacağımı ve hep böyle devam edeceğini düşünüyorum.

[Sonuç] Hem eski hayatımdan bir şeyler geri kazanmanın hem de bakım görevimi yerine getirmenin mümkün olup olmadığını merak ediyorum. Belki ikisini bir arada yürütmenin bir yolu vardır. Hem kendimi yeniden nasıl bulabilirim hem de babama en iyi şekilde bakmaya devam edebilirim?

Persona 3: Ağır bir yük altında olan, demans ve bununla nasıl başa çıkılacağı konusunda aile içinde farklı görüşler nedeniyle sıkıntı çeken, bakımı istediği gibi organize edemeyen bir kişi

Hikaye paketine genel bakış

**Hikaye**	**Konu**
Hikaye 3.1	Giriş
Hikaye 3.2	Aile içinde farklı görüşler
Hikaye 3.3	Ailevi çatışmalarla başa çıkma
Hikaye 3.4	Demansla ilgili damgalamalardan uzak durma
Hikaye 3.5	Güçsüzlükle başa çıkma

Persona 3: Ağır bir yük altında olan, demans ve bununla nasıl başa çıkılacağı konusunda aile içinde farklı görüşler nedeniyle sıkıntı çeken, bakımı istediği gibi organize edemeyen bir kişi

Hikaye 3.1: Giriş

[Giriş] Annem demans hastası ve babama onun bakımında destek oluyorum. Ana bakım veren kişi ben olmasam da elimden geldiğince yardımcı olmaya çalışıyorum. Ne yazık ki aile içinde annemin hastalığıyla nasıl başa çıkmamız gerektiği konusunda sürekli çatışmalar yaşanıyor.

[Sorun Tanımı] Babam, annemin sadece daha fazla ilgiye ihtiyacı olduğunu ve kendini toparlayabileceğini düşünüyor. Kız kardeşim ise onu daha çok kendi haline bırakmamız ve bu kadar mesele yapmamamız gerektiğini savunuyor. Ben ise profesyonel yardım almamız gerektiğine inanıyorum, ancak bu fikir aile içinde dirençle karşılanıyor. Kendimi ifade etmeye çalışıyorum ama kimse beni ciddiye almıyor gibi hissediyorum. Her şeyi değiştiremeyeceğimi biliyorum, ama anneme en iyi bakımı sağlayabilmek istiyorum—sürekli tartışmak zorunda kalmadan.

[Sonuç] Bazen kendimi iki taraf arasında sıkışıp kalmış gibi hissediyorum. Annemin acı çektiğini görüyorum, ancak bakımı olması gerektiği gibi yönlendirebilmek için elimden hiçbir şey gelmiyor. Ailemi birlikte hareket etmeye nasıl teşvik edebilirim ve bakım sürecindeki rolümü daha iyi nasıl üstlenebilirim?

LVF Lehrstuhl für Versorgungsforschung Universität Witten/Herdecke
ASH Berlin Alice Salomon Hochschule Berlin University of Applied Sciences
Demenz Support Stuttgart Zentrum für Informationstransfer

Persona 3: Ağır bir yük altında olan, demans ve bununla nasıl başa çıkılacağı konusunda aile içinde farklı görüşler nedeniyle sıkıntı çeken, bakımı istediği gibi organize edemeyen bir kişi

Story 3.2: Aile içinde farklı görüşler

[Giriş] Dün yine annemiz hakkında hararetli bir tartışma yaşadık. Babam, ona ilaç vermememiz gerektiğini düşünüyor çünkü bunun her şeyi daha da kötüleştireceğini söylüyor. Kız kardeşim de aynı fikirde, ancak ben farklı düşünüyorum.

[Sorun Tanımı] Aile içinde ortak bir yol bulmak gerçekten çok zor. Hepimizin farklı şeyler istediğini hissediyorum ve bu, annemin gerçekten neye ihtiyacı olduğunu unutmamıza neden oluyor. Tıbbi destek alarak daha iyi bir şekilde başa çıkabileceğimize inanıyorum, ancak sürekli olarak fikrim göz ardı ediliyor ya da ciddiye alınmıyorum. Annemin durumu kötüleşecek diye korkuyorum, eğer bir şey yapmazsak.

[Sonuç] Bazen, gerçekten bir şey değiştirebilir miyim diye düşünüyorum. Annem için en iyisini istiyorum, ama bu nasıl mümkün olacak, eğer sürekli birbirimizle anlaşmazlık yaşıyoruz? Aileme, profesyonel yardım almamız gerektiğini nasıl anlatabilirim, daha fazla çatışma yaşamadan?

Persona 3: Ağır bir yük altında olan, demans ve bununla nasıl başa çıkılacağı konusunda aile içinde farklı görüşler nedeniyle sıkıntı çeken, bakımı istediği gibi organize edemeyen bir kişi

Hikaye 3.3: Ailevi çatışmalarla başa çıkma

[Giriş] Son tartışmamızın ardından, kız kardeşimle sakin bir şekilde konuşmayı denedim. Neden bakım danışmanlığı almak gibi bir önerime karşı bu kadar karşı olduğunu anlamak istedim. Onun cevabı beni şaşırttı: Beni, çok bunalmış hissediyor ve annemizi bir bakımevine gönderebileceğimizden korkuyor.

[Sorun Tanımı] Çatışmalarımızın çoğunun korkular ve belirsizliklerden kaynaklandığını fark ettim. Ama bunlar hakkında dürüstçe konuşmak yerine, birbirimize ne kadar yanlış yaptığımızı söylüyoruz. Bu beni çok yıpratıyor çünkü birlikte bir çözüm bulmamızı istiyorum – annemiz için ve aile olarak bizim için.

[Sonuç] Belki hepimiz, birbirimize suçlamadan konuşmayı daha iyi öğrenmeliyiz. Ama böyle bir konuşmaya nasıl başlanır, herkes bu kadar gerginken? Aile içindeki gerilimi nasıl azaltabiliriz ve duygularımız ve korkularımız hakkında daha açık bir şekilde konuşabiliriz?

Persona 3: Ağır bir yük altında olan, demans ve bununla nasıl başa çıkılacağı konusunda aile içinde farklı görüşler nedeniyle sıkıntı çeken, bakımı istediği gibi organize edemeyen bir kişi

Story 3.4: Demansla ilgili damgalamalardan uzak durma

[Giriş] Birkaç gün önce bir komşum bana, annemin neden bu kadar garip davrandığını sordu. Ona demans hastalığına yakalandığını açıkladım, ama tepkisi beni çok etkiledi: “Bu kesinlikle zor olmalı sizin için – umarım millet bunu fazla fark etmez.”

[Sorun Tanımı] Çevremizdeki birçok kişinin demansın ne anlama geldiğini anlamadığını hissediyorum. Sanki hastalık, hakkında konuşulmaması gereken bir tabu. Babam, aile dışında kimseye bundan bahsedilmesini istemiyor, kız kardeşim ise bu tür sohbetlerden tamamen kaçınıyor. Ama bence, önyargıları ve damgalamayı kırmak için açıkça konuşmalıyız. Aile içinde de hastalık hakkında açıkça konuşmakta zorlanıyoruz.

[Sonuç] Bu hastalık hakkında konuşmanın, kendimizi kötü ya da yargılanmış hissetmeden nasıl mümkün olabileceğini merak ediyorum. Belki bu, aile olarak daha iyi başa çıkmamıza da yardımcı olur. Demansla nasıl açıkça konuşabiliriz ve aynı zamanda ailedeki diğer insanların endişe ve korkularını nasıl dikkate alabiliriz?

Persona 3: Ağır bir yük altında olan, demans ve bununla nasıl başa çıkılacağı konusunda aile içinde farklı görüşler nedeniyle sıkıntı çeken, bakımı istediği gibi organize edemeyen bir kişi

Story 3.5: Güçsüzlükle başa çıkma

[Giriş] Sürekli olarak hiçbir şeyi değiştiremeyeceğimi hissediyorum. Ne kadar çaba sarf edersem ederim, nihai kararları başkaları veriyor ve ben endişelerim ve hayal kırıklıklarımla geride kalıyorum.

[Sorun Tanımı] Bakımda çoğu zaman sadece yan bir rol oynadığımı kabullenmekte zorlanıyorum. Yanlış giden şeyleri görüyorum, ama buna dikkat çektiğimde ya görmezden geliniyor ya da dikkate alınmıyorum. Güçsüz hissetmek beni çok yoruyor. Gerçekten bir şeyler değiştirebilecek miyim, diye düşünüyorum.

[Sonuç] Bazen bırakmayı öğrenmem gerektiğini düşünüyorum – ama annemin bundan zarar görebileceğini bildiğimde bunu nasıl yapabilirim? Güçsüzlüğümle nasıl başa çıkabilirim ve yine de bakıma olumlu bir katkı sağlayabilirim?

Persona 4: Kendi ihtiyaçlarını ve iyiliğini, başkalarının beklentilerini karşılamak ve sosyal barışı sağlamak için sevgiyle geri planda tutan ve bakım veren rolünü kabul eden bir kişi

Hikaye paketine genel bakış

Hikaye	Konu
Hikaye 4.1	Giriş
Hikaye 4.2	Kendi Kişiliğini Kaybetmesi
Hikaye 4.3	Destek kabulü
Hikaye 4.4	Özverinin duygusal yükü
Hikaye 4.5	Kendi ihtiyaçlarıyla başa çıkma

Persona 4: Kendi ihtiyaçlarını ve iyiliğini, başkalarının beklentilerini karşılamak ve sosyal barışı sağlamak için sevgiyle geri planda tutan ve bakım veren rolünü kabul eden bir kişi

Hikaye 4.1: Giriş

[Giriş] Anneme bakmaya karar verdim, çünkü günlük hayatında giderek daha fazla yardıma ihtiyacı olmaya başladı. Şu anda iki yıldır benimle yaşıyor ve ona günün her saati bakıyorum. Onu bir huzur evine vermeyi asla düşünmem – bu onun için doğru bir şey değil. Ailemde, özellikle zor zamanlarda birbirimize destek olmak bir gelenektir. Ve onun kızı olarak, ona bakmak benim görevim.

[Sorun Tanımı] Bazen nasıl hala işleyebildiğimi merak ediyorum. Kendime neredeyse hiç zamanım kalmıyor ve çoğu zaman tükenmiş hissediyorum. Ama şikayet etmek istemiyorum – sonuçta bu benim sorumluluğum, ve bu görevi sevgiyle üstlendim. Annem yardımımı ihtiyaç duyduğunda, kendime iyi bir şey yapmayı günah olarak hissediyorum. Ama bazen her şey fazla geliyor ve böyle devam edebileceğimden emin değilim.

[Sonuç] Ona bakmayı bırakamam – bu yanlış hissederdim. Ama aynı zamanda, kendi sağlığımı ve refahımı kaybetmeden bunu ne kadar sürdürebileceğimi merak ediyorum. Bu sürekli özveriyle nasıl başa çıkabilirim, kimliğimi ve yaşam kalitemi tamamen kaybetmeden?

Persona 4: Kendi ihtiyaçlarını ve iyiliğini, başkalarının beklentilerini karşılamak ve sosyal barışı sağlamak için sevgiyle geri planda tutan ve bakım veren rolünü kabul eden bir kişi

Story 4.2: Kendi Kişiliğini Kaybetmesi

[Giriş] Son birkaç ayda, hayatımın ne kadar değiştiğini fark ettim. Eskiden hobilerim, arkadaşlarım ve kendi isteklerim ve hedeflerim vardı. Ama şimdi her şey annemin bakımına odaklanmış durumda. Kendimi kaybetmiş gibi hissediyorum.

[Sorun Tanımı] Sık sık, "bakıcı" olmasam kim olduğumu merak ediyorum. Anneme bakıyor, ev işlerini yapıyor ve gece yardımına ihtiyaç duyduğunda uykum bile sık sık kesiliyor. Arkadaşlarım uzun zamandır benimle iletişime geçmedi ve beni bir zamanlar tanımlayan her şeyle bağlantımı kaybettim. Onun için var olmak adına sahip olduğum her şeyi feda ediyorum.

[Sonuç] Tamamen kaybolmuş gibi hissediyorum. Sürekli olarak sadece onun için yaşarken, kendime nasıl geri dönebilirim? Anneme hala bakarken, kendi kimliğimi nasıl geri kazanabilirim?

Persona 4: Kendi ihtiyaçlarını ve iyiliğini, başkalarının beklentilerini karşılamak ve sosyal barışı sağlamak için sevgiyle geri planda tutan ve bakım veren rolünü kabul eden bir kişi

Hikaye 4.3: Destek kabulü

[Giriş] Dün, kardeşim annemi doktora götürmeyi teklif etti, böylece biraz dinlenebileceğim. İyi niyetli olduğunu biliyorum ama teklifi kabul edemedim. Başkasının anneme bakmasına izin vermek bana doğru gelmiyor – o zaman rahatlayamam.

[Sorun Tanımı] Yardım kabul etmek bana inanılmaz derecede zor geliyor. Her zaman daha iyi yapabileceğimi düşünüyorum ve onu yalnız başıma bakmanın benim görevim olduğunu hissediyorum. Bazen yardım istemek bile utanılacak bir şey gibi geliyor çünkü zayıflık olarak algılanacağını düşünüyorum. Yardım kabul edersem sorumluluğumu terk etmiş olacağımı hissediyorum.

[Sonuç] Bir ara vermenin bana iyi geleceğini biliyorum ama bırakmak bir türlü içime sinmiyor. Kendimi öne çıkarmak yanlış gibi geliyor. Yardım kabul etmeyi, sorumluluğumu kaybetmiş gibi hissetmeden nasıl öğrenebilirim?

Persona 4: Kendi ihtiyaçlarını ve iyiliğini, başkalarının beklentilerini karşılamak ve sosyal barışı sağlamak için sevgiyle geri planda tutan ve bakım veren rolünü kabul eden bir kişi

Story 4.4: Özverinin duygusal yükü

[Giriş] Kendimden giderek daha az şey kaldığını hissediyorum. Duygusal yük çok ağır – her zaman güçlü olmaya çalışıyorum ve ne kadar yorgun olduğumu kimseye belli etmemeye çalışıyorum. Ama son birkaç haftadır içsel olarak tamamen tükenmiş hissettiğimi fark ediyorum.

[Sorun Tanımı] Annem için sürekli burada olmak, hiç ara vermemek çok zor. Ona ne kadar çok sevgi ve bakım vermek istesem de, aynı zamanda omuzlarımdaki yükün gittikçe daha da ağırlaştığını hissediyorum. Bazen gizlice ağlıyorum çünkü bunu daha ne kadar dayanabileceğimi bilmiyorum. Ama bu konuda konuşabileceğim kimse yok ve başarısız olduğumuzu düşünmelerini istemiyorum.

[Sonuç] Bu yükü daha ne kadar taşıyabileceğimi merak ediyorum. Sanki sadece bakıcıymışım gibi hissediyorum – kendi duygularım veya ihtiyaçlarım için hiçbir alanım yok. Duygusal yükle nasıl başa çıkabilirim, bakımı bırakmak ya da ailemi hayal kırıklığına uğratmak zorunda kalmadan?

Persona 4: Kendi ihtiyaçlarını ve iyiliğini, başkalarının beklentilerini karşılamak ve sosyal barışı sağlamak için sevgiyle geri planda tutan ve bakım veren rolünü kabul eden bir kişi

Story 4.5: Kendi ihtiyaçlarıyla başa çıkma

[Giriş] Kendi ihtiyaçlarımı kabul etmek benim için zor. Bazen bir molaya ihtiyacım olduğunu biliyorum, ama bir türlü bunu almaya karar veremiyorum. Kendimi düşünmenin bencilce olduğunu hissediyorum.

[Sorun Tanımı] Her gün annemin ihtiyaçları etrafında dönüyor – ve kendim için hiç zamanım yokmuş gibi hissediyorum. Bakıcılık dışında bir hayatım yok, arkadaşlıklarım için zamanım yok, kendi ilgi alanlarım için zamanım yok. Sık sık birkaç saat sadece kendim için olmanın nasıl bir şey olacağını düşünüyorum, ama hemen fark ediyorum ki bunu yapamam. Kendi ihtiyaçlarım önemsiz gibi hissediyorum. Önemli olan tek şey, annemin iyi olması ve ona bakabilmem.

[Sonuç] Anneme bakmayı çok sevsem de, bazen kendime de dikkat etmeyi nasıl başarabileceğimi merak ediyorum, bunun için suçluluk hissetmeden. Kendi ihtiyaçlarımı tanımayı nasıl öğrenebilirim ve annemin bakımını sevgiyle üstlenmeye devam edebilirim?

Persona 5: Bakım sorumluluğunu öncelikle uygun koşullar, geleneksel/kültürel normlar, aile beklentileri ve hiyerarşik yapılar nedeniyle üstlenen bir kişi

Hikaye paketine genel bakış

Hikaye	Konu
Hikaye 5.1	Giriş
Hikaye 5.2	Ailevi sorumluluklar
Hikaye 5.3	Toplumsal ve kültürel beklentiler
Hikaye 5.4	Az destek ve yalnız sorumluluk
Hikaye 5.5	Kendini gerçekleştirme ve kişisel yaşam

Persona 5: Bakım sorumluluğunu öncelikle uygun koşullar, geleneksel/kültürel normlar, aile beklentileri ve hiyerarşik yapılar nedeniyle üstlenen bir kişi

Hikaye 5.1: Giriş

[Giriş] Demans hastalığına yakalanmış kayınvalideme bakıyorum. Bu sorumluluğu üstlenip üstlenmeyeceğim hiç bir soru olmadı – bunu yapmam gerektiği belliydi. Ailemizde, genç nesillerin yaşlılara bakması gelenekseldir, özellikle hasta olduklarında. Bu sorumluluk genellikle kadınlara aittir. Eşimde kız kardeş olmadığı için, kayınvalideme bakmak benim görevim.

[Sorun Tanımı] Bunun hakkında nasıl hissettiğimi hiç gerçekten düşünmemiştim. Her şeyin her zaman böyle yapıldığı gibi. Ama bakım sorumluluğunu üstlendikçe, çoğu zaman kendi isteklerimden ve ihtiyaçlarımdan feragat etmek zorunda kaldığımı daha çok fark ediyorum. Kendi hobilerime ya da arkadaşlarıma zaman ayıracak hiç vaktim yok – her şey kayınvalideme bakmakla ilgili. Bu süreçte kendi kimliğimi kaybettiğimi hissediyorum.

[Sonuç] Bakımı üstlenmek zorunda olduğum hissinden kurtulmak zor. Aileme karşı görev ve sevgiyle yapıyorum bunu, ama bazen kendi isteklerimi ihmal etmeden daha ne kadar devam edebileceğimi merak ediyorum. Kayınvalideme olan sorumluluğumla kendi ihtiyaçlarım arasında, ailevi ve toplumsal beklentileri yerine getirmediğimi hissetmeden nasıl bir denge kurabilirim?

Persona 5: Bakım sorumluluğunu öncelikle uygun koşullar, geleneksel/kültürel normlar, aile beklentileri ve hiyerarşik yapılar nedeniyle üstlenen bir kişi

Story 5.2: Ailevi sorumluluklar

[Giriş] Ailemde kayınvalideme bakım verme kararı hiç tartışılmadı. Bu, bizim kültürümüzde ve ailemizde yapılan bir şeydir. Yaşlılar her zaman gençlere bakmışlardır, şimdi ise sıranın bana geldiği bir dönemdesiyim.

[Sorun Tanımı] Bazen bu kararı kendi inancımla mı aldım, yoksa sadece aile geleneği gereği mi aldım diye düşünüyorum. Çoğu zaman, bir seçim yokmuş gibi hissediyorum – bakım sorumluluğunu üstlenmezsem, tüm aileyi hayal kırıklığına uğratırım. Ama derinlerde, bu sorumluluktan gerçekten mutlu olup olmadığımı sorguluyorum.

[Sonuç] Bazen, kendi hayatıma daha fazla odaklanabilir miyim diye düşünüyorum. Ancak aileme karşı olan sorumluluğum ve kültürel normlar her seferinde beni geri tutuyor. Kendi isteklerimi yerine getirirken, ailevi sorumluluğumu ihlal etmeden bunu nasıl öğrenebilirim?

Persona 5: Bakım sorumluluğunu öncelikle uygun koşullar, geleneksel/kültürel normlar, aile beklentileri ve hiyerarşik yapılar nedeniyle üstlenen bir kişi

Hikaye 5.3: Toplumsal ve kültürel beklentiler

[Giriş] Annem her zaman, aileyle ilgilenmenin benim görevim olduğunu, özellikle yaşlandıkça, söyledi. Bizim kültürümüzde, çocukların ebeveynlerine bakması beklenen bir şeydir. Ayrıca toplumumuzda, bu sorumluluğu taşımak bir güç olarak görülür. Yardıma ihtiyacı olanlara yardım etmeyi severim, ancak bunu bilinçli olarak seçmeyi dilerdim.

[Sorun Tanımı] Kendimi baskı altında hissediyorum – hem ailemin beklentileri hem de toplumsal normlar nedeniyle. Yardım kabul ettiğimde veya biraz ara vermek istediğimde, diğer aile üyeleri tarafından sıklıkla dolaylı yoldan bana, güçlü olmam gerektiği ve bunu başarabileceğim hatırlatılıyor. Kendi ihtiyaçlarımı bu beklentilerle karşı karşıya koymak sürekli bir zorluk.

[Sonuç] Acaba, "Bir ara vermem gerekiyor" diyebilme hakkım olacak mı? Ve aslında bunu yapmalı mıyım? Çünkü kayınvalidemin iyi olmasını da istiyorum. Bu sorumluluk hissinden nasıl kurtulabilirim ve kendi ihtiyaçlarımı ön plana çıkarmama nasıl izin verebilirim?

Persona 5: Bakım sorumluluğunu öncelikle uygun koşullar, geleneksel/kültürel normlar, aile beklentileri ve hiyerarşik yapılar nedeniyle üstlenen bir kişi

Story 5.4: Az destek ve yalnız sorumluluk

[Giriş] Kayınvalideme bakmak için tek başıma ben varım – ailede kimse gerçekten destek olmaya istekli ya da uygun görünmüyor. Her zaman her şeyi tek başıma yapmak zorundaymışım gibi hissediyorum, bu da her şeyi daha da zorlaştırıyor.

[Sorun Tanımı] Destek neredeyse hiç yok, ama her şeyi yapmanın benim görevim olduğunu hissediyorum. Eşim ve kardeşleri ya çok meşguller ya da müdahale etmelerinin gerekli olmadığını düşünüyorlar. Sıklıkla her şeyi tek başıma omuzlamam gerektiğini hissediyorum.

[Sonuç] Yalnızca ben taşıyorum, bu büyük bir yük ve bazen kimse yardımcı olmuyorken bunu nasıl başarabildiğimi merak ediyorum. Yardım kabul etmeyi nasıl öğrenebilirim, sorumluluğu kaybetmiş gibi hissetmeden?

Persona 5: Bakım sorumluluğunu öncelikle uygun koşullar, geleneksel/kültürel normlar, aile beklentileri ve hiyerarşik yapılar nedeniyle üstlenen bir kişi

Story 5.5: Kendini gerçekleştirme ve kişisel yaşam

[Giriş] Son birkaç yılımı sadece kayınvalideme bakmakla geçirdim. Eskiden planlarım vardı, seyahat etmek ve yeni şeyler denemek istiyordum, ama artık bunların hiçbirine yer yok. Tüm zamanım bakıma gidiyor ve kendimi kaybettiğimi hissediyorum.

[Sorun Tanımı] Sadece bakımın fiziksel yükü değil, aynı zamanda tatmin eksikliği de var. Kendi hayallerim ve hedeflerim ne oldu diye düşünüyorum. Kendim için bir şey yapma şansım hiç olmadı. Kendimi bir gölge versiyonu gibi hissediyorum – her zaman sadece “bakıcı” rolünde.

[Sonuç] Şu an hayatımın neredeyse tamamen bakım ile belirlendiğini kabul etmek zor. Ama aileme karşı duyduğum sorumluluk, kendimi gerçekleştirmem için çok az yer bırakıyor. Ailemden birini terk etmiş gibi hissetmeden kendi hayatımı yeniden nasıl yaşayabilirim?

Persona 6: Diğer aile üyelerinin bakım yapmaya yeterli olmadığına inanan ve profesyonel destek kullanan, tek başına, aktif, kontrolcü ve kararlı bir kişi

Hikaye paketine genel bakış

Hikaye	Konu
Hikaye 6.1	Giriş
Hikaye 6.2	Diğer aile üyelerine güvenmeme
Hikaye 6.3	Kontrol ve bunaltılma
Hikaye 6.4	Mükemmeliyetçilik ihtiyacı
Hikaye 6.5	Profesyonel destek

Persona 6: Diğer aile üyelerinin bakım yapmaya yeterli olmadığına inanan ve profesyonel destek kullanan, tek başına, aktif, kontrolcü ve kararlı bir kişi

Hikaye 6.1: Giriş

[Giriş] Anneme ben bakıyorum çünkü başkasının doğru yapacağına inanmıyorum. Kardeşlerim ya çok meşgul ya da bunun büyük bir şey olmadığını düşünüyorlar. Annemin bakımını benim kadar ciddiye almıyorlar. Bu yüzden kontrolü ele almaya ve her şeyi kendim halletmeye karar verdim. Onun en iyi ellerde olduğundan emin oluyorum, bu da demek oluyor ki kendi ihtiyaçlarımı arka plana atıyorum.

[Sorun Tanımı] Diğer aile üyelerine görev devretmekte zorlanıyorum. Bazen bunun doğru şekilde yapılıp yapılmayacağından emin olamıyorum. Bu yüzden profesyonel destek de alıyorum. Ama burada da kontrolü elinde tutuyorum çünkü her şeyin düzgün gitmesini ve annemin bakımının garanti altına alınmasını istiyorum.

[Sonuç] Her şeyi izlemek yorucu ve bazen tükenmiş hissediyorum. Ama duramıyorum çünkü başkasının sorumluluğu aynı ciddiyetle almayacağını ve annemin bundan zarar görebileceğini düşünüyorum. Kontrolü kaybetmeden sorumluluğu nasıl paylaşmayı öğrenebilirim?

Persona 6: Diğer aile üyelerinin bakım yapmaya yeterli olmadığına inanan ve profesyonel destek kullanan, tek başına, aktif, kontrolcü ve kararlı bir kişi

Story 6.2: Diğer aile üyelerine güvenmeme

[Giriş] Kardeşlerim her zaman bana yardımcı olmaya hazır, ama onlara güvenemiyorum. Annemize bakmanın ne anlama geldiğini hiç gerçekten anlamadılar. Ve ne kadar iyi niyetli olsalar da, bunun bana olduğu kadar iyi ve yeterince iyi yapacaklarını bilmiyorum.

[Sorun Tanımı] Sorumluluğu devretmekte zorlanıyorum. Ne olup bittiğini tam olarak bilmediğimde rahat olamıyorum. Bir şeyleri gözden kaçırabilirler ya da yanlış yapabilirler. Bu düşünceler beni huzursuz ediyor ve her şeyi kendim yapmama neden oluyor – bu beni bunaltıyor. Sonra bırakmam gerektiğini ve sorumluluğu devretmem gerektiğini düşünüyorum.

[Sonuç] Böyle devam edebileceğimi merak ediyorum. Belki bir süre sonra gerçekten gücüm kalmayacak, ama başkasının bakımı devralması düşüncesi beni sinirlendiriyor ve güvensiz hissettiriyor. Başkalarına güvenmeyi ve sorumluluğu paylaşmayı nasıl öğrenebilirim, sürekli kontrolü kaybetme hissi olmadan?

Persona 6: Diğer aile üyelerinin bakım yapmaya yeterli olmadığına inanan ve profesyonel destek kullanan, tek başına, aktif, kontrolcü ve kararlı bir kişi

Hikaye 6.3: Kontrol ve bunaltılma

[Giriş] Her şeyi tek başıma halletmek kolay değil. Bakım üzerinde kontrolü elinde tutmam gerektiğini biliyorum, böylece hiçbir şey ters gitmez. Ama aynı zamanda, bu sorumluluğun beni ne kadar yüklediğini fark ediyorum. Sanki sürekli tetikte olmam gerekiyormuş gibi hissediyorum.

[Sorun Tanımı] Profesyonel bakımcıların desteğine rağmen, her zaman orada olmam gerektiğini hissediyorum. Olup biten her şeyi kontrol ediyor ve her şeyin planlarıma göre gittiğinden emin oluyorum. Bu kontrolü sürdürmek yorucu, ama kimsenin bunu benim kadar iyi yapabileceğini düşünüyorum. Yine de, her şeyi kontrol etmeye çalıştıkça, bunun beni nasıl tükettiğini daha çok fark ediyorum.

[Sonuç] Kontrol ve bunaltıcı sorumluluk arasında denge kurmak sürekli bir zorluk. Bunu tamamen tükenmeden daha ne kadar sürdürebileceğimi bazen merak ediyorum. Annemin bakımının bundan etkilenmeyeceğinden emin olmadan, bir miktar kontrolü nasıl bırakabilirim?

Persona 6: Diğer aile üyelerinin bakım yapmaya yeterli olmadığına inanan ve profesyonel destek kullanan, tek başına, aktif, kontrolcü ve kararlı bir kişi

Story 6.4: Mükemmeliyetçilik ihtiyacı

[Giriş] Annem için her şeyin en iyi durumda olduğundan emin oluyorum. Profesyonel yardım aldım, ama her şeyi kontrol ediyorum çünkü birkaç kez onların bir şeyleri unuttuğuna şahit oldum. Annemin en iyi ellerde olmasını ve en iyi desteği almasını istiyorum. Bu yüzden, birinin bir şeyleri yanlış yapması ya da benim doğru bildiğim yöntem dışında bir yöntem kullanması beni zor durumda bırakıyor.

[Sorun Tanımı] Her şeyi mükemmel yapmak gittikçe zorlaşıyor, özellikle de her şeyi tek başıma yapamadığım için. Annemin iyi bakılabilmesi için bazı görevleri devretmek zorundayım. Ama bunu yaparken sürekli stresli hissediyorum. Neredeyse her şeyin mükemmel bir şekilde düzenli kalmasını sağlamak için bir bekçi gibi hissediyorum.

[Sonuç] Acaba anneme mükemmel bir bakım verebilecek miyim, yoksa sonunda her şeyin her zaman mükemmel olmayacağını kabullenmek zorunda mı kalacağım? Ancak gevşemek fikri beni korkutuyor, çünkü bir şeylerin yanlış gitmesinden ve annemin bunun bedelini ödemesinden korkuyorum. Bakımı daha az sıkı kontrol etmeyi nasıl öğrenebilirim, bunun tavizlere yol açtığını hissetmeden?

Persona 6: Diğer aile üyelerinin bakım yapmaya yeterli olmadığına inanan ve profesyonel destek kullanan, tek başına, aktif, kontrolcü ve kararlı bir kişi

Story 6.5: Profesyonel destek

[Giriş] Artık her şeyi tek başıma yapamayacağım için profesyonel bakım hizmeti aldım. Bu, hem benim için hem de annem için büyük bir adımdı. Her şeyin iyi gitmesi için bakımı yakından takip ediyorum. Bakım çalışanlarıyla çok konuşuyorum ve bir şeyler konuşulması gerektiğinde doğrudan iletişimde olan kişiyim.

[Sorun Tanımı] Yardıma ihtiyacım olduğunu biliyorum, ama bu yardımı gerçekten kabul etmekte zorlanıyorum. Bir başkasının bakımın nasıl yapılacağına karar vermesi bana doğru gelmiyor. Annem için en iyi olanı yaptıklarından emin olmak istiyorum. Ama tabii ki, bakım çalışanlarının bakım konusunda uzman olduklarını da biliyorum ve kontrolü – en azından biraz – bırakabilirim.

[Sonuç] Yardım almakla, aynı zamanda her şeyi kendi başıma belirliyormuş gibi hissetmek arasında sürekli bir zorluk var. Acaba bir gün gerçekten bırakıp profesyonel bakım çalışanlarına güvenebilecek miyim? Bana sunulan desteği, gereksiz ya da kontrol altında hissedilmeden nasıl kabul edebilirim?

Persona 7: Kendi takdirine bağlı olarak, kendi yaşamıyla uyumlu bir şekilde ve profesyonel destekle bakım kararlarını rasyonel ve organize bir şekilde veren bir kişi

Hikaye paketine genel bakış

Hikaye	Konu
Hikaye 7.1	Giriş
Hikaye 7.2	Bakım ve organizasyon
Hikaye 7.3	Kararlar almak
Hikaye 7.4	Profesyonel destek
Hikaye 7.5	Duygusal dengeyi bulmak

Persona 7: Kendi takdirine bağlı olarak, kendi yaşamıyla uyumlu bir şekilde ve profesyonel destekle bakım kararlarını rasyonel ve organize bir şekilde veren bir kişi

Hikaye 7.1: Giriş

[Giriş] Aslında ana bakım veren ben değilim, ancak annemizin bakımının iyi yürütülmesini sağlıyorum. Çalışıyorum ve kendi ailem var, bu yüzden asıl bakımı kız kardeşim ve özellikle de tuttuğum bakım hizmetleri üstleniyor. Bakımın organizasyonundan sorumlu olmayı hedefliyorum. Annemle birlikte neye ihtiyacı olduğuna karar veriyor ve doğru yardımı almasını sağlıyorum. Beni ve kız kardeşimi destekleyen profesyonel bakım çalışanlarını da işe aldım.

[Sorun Tanımı] Her şeyin iyi organize edilmesi benim için önemli, ancak bakıma duygusal olarak fazla dahil olmuyorum. Bunun yapılması gereken bir görev olduğunu biliyorum ve bu yüzden her şeyi planlı bir şekilde yürütüyorum. Annemin ihtiyacı olan her şeyi almasını sağlıyorum, ancak bakımın kendi ihtiyaçlarımı fazla zorlamasına izin vermiyorum.

[Sonuç] Bu şekilde gayet iyi işliyor. Her şeyi organize edebiliyorum ve duygusal yönleriyle fazla yüzleşmek zorunda kalmıyorum. Ancak bazen kız kardeşim bana çok fazla "organize ettiğimi" ve annemin demansının duygusal yönlerini ve sağlık durumunu göz ardı ettiğimi hissettiriyor. Bakımın iyi organize edilmesini sağlarken aynı zamanda anneme ve kız kardeşime yeterince duygusal destek sunduğumdan nasıl emin olabilirim?

Persona 7: Kendi takdirine bağlı olarak, kendi yaşamıyla uyumlu bir şekilde ve profesyonel destekle bakım kararlarını rasyonel ve organize bir şekilde veren bir kişi

Story 7.2: Bakım ve organizasyon

[Giriş] Bakımı organizasyonel bir görev olarak görüyorum. Randevuların yerine getirilmesini, doğru destek kişilerin hazır bulunmasını ve annem için gerekli her şeyin düzenlenmesini sağlıyorum. Amacım, hiçbir şeyin unutulmamasını sağlamak için her şeyi koordine etmek. Kız kardeşim ise duruma tamamen farklı bakıyor. O, duygusal olarak çok fazla içinde yer alıyor.

[Sorun Tanımı] Her bir görevi bizzat yerine getirmiyorum, ancak her şeyin plana uygun ilerlemesini sağlıyorum. Bakım çalışanlarıyla düzenli olarak konuşuyor ve işlerin yolunda gittiğinden emin oluyorum. Annemi doktor randevularına da ben götürüyorum. Ancak kız kardeşim bazen duruma fazla tarafsız yaklaştığımı ve bakımın duygusal yönünü ihmal ettiğimi söylüyor.

[Sonuç] Her şeyin sorunsuz işlemesini istiyorum, ancak belki de annemin ihtiyaçlarına daha fazla kulak verip organizasyona daha az odaklanmalıyım. Bakımın organizasyonu ile anneme duygusal destek sağlamak arasında daha iyi bir dengeyi nasıl bulabilirim?

Persona 7: Kendi takdirine bağlı olarak, kendi yaşamıyla uyumlu bir şekilde ve profesyonel destekle bakım kararlarını rasyonel ve organize bir şekilde veren bir kişi

Hikaye 7.3: Kararlar almak

[Giriş] Annemin bakımıyla ilgili kararları ben veriyorum, ancak bu durum beni duygusal olarak zorlamıyor. Aileyi ve bakım çalışanlarını sürece dahil ediyorum, ancak yönü belirleyen kişi benim. Kontrolün bende olması bana iyi hissettiriyor, ancak bazen annemle yeterince konuşup nasıl hissettiğini gerçekten anlayıp anlamadığımı merak ediyorum.

[Sorun Tanımı] Her şeyin düzgün işlemesi önemli, ancak duygusal yönleri sürekli düşünmüyorum. Onun için en iyi olanı pragmatik bir şekilde belirliyorum. Ancak bu yaklaşımım nedeniyle, annem için önemli olabilecek ama doğrudan bakım ile ilgili olmayan şeyleri gözden kaçırıyor olabilirim.

[Sonuç] Organizasyon konusunda iyi olduğumu biliyorum, ancak bakım kararlarını alırken annemin duygusal ihtiyaçlarını daha fazla dikkate almam gerekip gerekmediğini düşünüyorum. Ayrıca, annemle çok fazla vakit geçiren kız kardeşimi sürece daha fazla dahil etmem iyi olur mu diye sorguluyorum. Annemin duygusal desteğe ihtiyaç duyduğunu daha iyi nasıl fark edebilirim, bunu yaparken bakımın pratik organizasyonunu da aksatmamak için ne yapmalıyım?

Persona 7: Kendi takdirine bağlı olarak, kendi yaşamıyla uyumlu bir şekilde ve profesyonel destekle bakım kararlarını rasyonel ve organize bir şekilde veren bir kişi

Story 7.4: Profesyonel destek

[Giriş] Günlük bakım işlerini üstlenen profesyonel bakım çalışanlarını işe aldım. Onlar iyi eğitimli ve annemin ihtiyaç duyduğu her konuda ona yardımcı olabiliyorlar. Karar alma süreçlerinde her zaman ulaşılabilirim, ancak pratik işleri bakım çalışanlarına bırakıyorum. Bu durum kız kardeşimi de rahatlatıyor.

[Sorun Tanımı] Finansal ve organizasyonel konularla ilgileniyorum ve bakımın günlük uygulamasını profesyonellere bırakıyorum. Her şey yolunda gidiyor, ancak bazen günlük bakım sürecine yeterince dahil olamadığımı hissediyorum.

[Sonuç] Annemin en iyi yardımı aldığından emin olmak istiyorum. Ancak bazen, diğer bakım verenler ya da kız kardeşim gibi, annemle duygusal bir bağ kurmada eksik kaldığımı hissediyorum. Günlük bakıma daha fazla nasıl dahil olabilirim, ancak tüm sorumluluğu da üstlenmeden bunu nasıl yapabilirim?

Persona 7: Kendi takdirine bağlı olarak, kendi yaşamıyla uyumlu bir şekilde ve profesyonel destekle bakım kararlarını rasyonel ve organize bir şekilde veren bir kişi

Story 7.5: Duygusal dengeyi bulmak

[Giriş] Annemin bakımıyla ilgilenirken duygusal olarak bağ kurmak benim için kolay değil. Her şeyi organize ediyorum, ancak iş duygulara gelince o kadar da içinde değilim. Onun iyi ellerde olmasını ve doğru desteği almasını sağlıyorum, ancak bazen annemin duygusal ihtiyaçları için yeterince çaba gösterip göstermediğimi merak ediyorum. Ayrıca, annemin hasta olması ve bakıma muhtaç olması beni neden bu kadar soğukkanlı bıraktığını sorguluyorum.

[Sorun Tanımı] Duygusal yönlere gelince bazen mesafeli durduğumu fark ediyorum. Belki de kendi korkularım ve endişelerimle yüzleşmek istemediğim için. Finansal ve organizasyonel işleri hallediyorum, ancak annemin duyguları veya istekleri hakkında çok sık konuşmuyorum. Onun iyi bakıldığından emin olmak istiyorum, ancak gerçekten neye ihtiyacı olduğunu yeterince dinleyip dinlemediğimi sorguluyorum.

[Sonuç] Belki de, güçlü yönüm olmasa bile, annemin duygusal ihtiyaçlarına daha fazla zaman ayırmalıyım. Peki, bakımın duygusal yönlerini günlük yaşamıma nasıl daha iyi entegre edebilirim, organizasyonel sorumluluklarımı ihmal etmeden bunu nasıl başarabilirim?

Persona 8: Bakım durumundan aktif bir şekilde uzaklaşan, en destekleyici ve daha çok gözlemleyen bir rol üstlenen bir kişi

Hikaye paketine genel bakış

**Hikaye**	**Konu**
Hikaye 8.1	Giriş
Hikaye 8.2	Bakımda destek
Hikaye 8.3	Bakımdan sınır koyma
Hikaye 8.4	Aile ve destek
Hikaye 8.5	Bakımdaki rol

Persona 8: Bakım durumundan aktif bir şekilde uzaklaşan, en destekleyici ve daha çok gözlemleyen bir rol üstlenen bir kişi

Hikaye 8.1: Giriş

[Giriş] Yardım edebileceğim yerlerde yardımcı oluyorum, ancak aslında daha çok dışarıdayım. Annemin bakımı çoğunlukla kız kardeşimin sorumluluğunda. Gerekirse ona destek oluyorum, ancak bakımın günlük işleyişine gerçekten dahil değilim. Daha çok diğer şeylerle ilgileniyorum ve bakımı kız kardeşime bırakıyorum.

[Sorun Tanımı] Yardıma ihtiyacı olduğunda oradayım, ancak bakım konusunda o kadar sorumlu hissetmiyorum. Her zaman üstlendiğim görevler var, örneğin alışveriş yapmak veya yemek pişirmek, ancak bakımın kendisi gerçekten bana ait değil. Kendimi çok fazla dahil etmeden nasıl daha fazla katkı sağlayabileceğimi bilmiyorum.

[Sonuç] Daha fazla şey yapmam gerektiğini düşünüyorum, ancak bir şekilde gerçekten sorumlu hissetmiyorum. Bakıma destek olmaya çalışıyorum, ancak aktif olarak katılmıyorum. Kendimi aşırı yüklenmiş hissetmeden bakımda rolümü nasıl değiştirebilirim?

Persona 8: Bakım durumundan aktif bir şekilde uzaklaşan, en destekleyici ve daha çok gözlemleyen bir rol üstlenen bir kişi

Story 8.2: Bakımda destek

[Giriş] Yardım gerektiğinde ara sıra oradayım, ancak açıkçası bakım hakkında çok bilgim yok. Kız kardeşim neredeyse her şeyi üstleniyor. Ben, yiyecek almak veya doktor randevuları düzenlemek gibi pratik destek sağlıyorum. Ama bakımın kendisiyle ilgili fazla bilgim yok.

[Sorun Tanımı] Genellikle doğru yardımın ne olduğunu bilmekte zorlanıyorum. Yeterince bir şey yapıp yapmadığımı veya daha fazla mı dahil olmam gerektiğini merak ediyorum. Ama gerçekten yardımcı olabilmek için bilgim eksik. Annemin neye ihtiyacı olduğunu anlamak benim için zor, özellikle bakım konusunda.

[Sonuç] Annemin neye ihtiyacı olduğunu daha iyi anlamak istiyorum, ancak nereden başlayacağımı bilmiyorum. Belki de daha fazla bilgi edinmeliyim. Kendimi aşırı yüklenmiş hissetmeden, bakım hakkında daha fazla nasıl öğrenebilirim?

Persona 8: Bakım durumundan aktif bir şekilde uzaklaşan, en destekleyici ve daha çok gözlemleyen bir rol üstlenen bir kişi

Hikaye 8.3: Bakımdan sınır koyma

[Giriş] Bakımı üstlenmedim çünkü kız kardeşimin bunu yapmasının daha iyi olduğunu düşünüyorum. Daha fazla deneyimi var ve istemediğim bir role itirilmek istemiyorum. Kendime güvendiğim görevlerde yardımcı oluyorum, ancak geri kalanında bakımdan uzak duruyorum.

[Sorun Tanımı] Kendimi doğru şekilde dahil etmekte zorlanıyorum çünkü fazla sorumluluk almak istemiyorum. Her gün bakımın içinde olmaktan rahat hissetmiyorum. Belki de bu yüzden bakımın yalnızca birkaç kişinin omuzlarına yüklenmesi beni bazen hayal kırıklığına uğratıyor, ancak şu anda yaptığımdan daha fazlasını yapmak istemiyorum.

[Sonuç] Zor bir durum çünkü kendimi ana bakım veren kişi olarak görmüyorum. Ama bir şekilde daha fazlasını yapmadığım için suçlu hissediyorum. Kendimi sınırlarımı aşmadan daha fazla nasıl dahil edebilirim?

Persona 8: Bakım durumundan aktif bir şekilde uzaklaşan, en destekleyici ve daha çok gözlemleyen bir rol üstlenen bir kişi

Story 8.4: Aile ve destek

[Giriş] Yardım gerektiğinde yardımcı oluyorum, ancak ailem bakım konusunda çoğunlukla sorumlu. Beni ihtiyaç duyduklarında yanlarında buluyorlar, ancak her şeyin sorumluluğunu almak gibi bir hisse kapılmıyorum. Sürekli bir şeyler yapmam gerektiği durumlar oluyor, ama gerçekten aktif bir şekilde devreye girmekte zorlanıyorum. Sonuçta annem hasta.

[Sorun Tanımı] Genellikle diğer aile üyelerinin daha fazla şey yapabileceğini düşünüyorum, ancak sürekli bakımın içine müdahale etmem gereken kişi ben değilim gibi hissediyorum. Destek olmak istiyorum, ama duygusal olarak mesafemi koruduğumu fark ediyorum. Bakıma tam olarak dahil olmak benim için zor ve çoğu zaman ailemin bu durumu kendi aralarında daha iyi halledebileceğini düşünüyorum. Öte yandan, aileme yardımcı olmak ve onları rahatlatmak istiyorum, durumun umurumda olmadığı izlenimi vermek istemiyorum. Ama aslında durumdan memnunum.

[Sonuç] Yardım gerektiğinde yanlarındayım, ancak bir şekilde annemin bakımında gerçekten fazla bir şey değiştiremeyeceğimi hissediyorum. Yardım etmek istiyorum. Bakıma çok fazla müdahale etmeden nasıl yardımcı olabilirim?

Persona 8: Bakım durumundan aktif bir şekilde uzaklaşan, en destekleyici ve daha çok gözlemleyen bir rol üstlenen bir kişi

Story 8.5: Bakımdaki rol

[Giriş] Annemin bakımını daha çok yapısal bir bakış açısından görüyorum. Yapılması gereken belirli görevler var ve ben bu görevleri desteklemek için buradayım. Ama annemin nasıl olduğunu ya da herkesin nasıl hissettiğini sormak söz konusu olduğunda daha mesafeliyim. Yapmam gerekeni yapıyorum, ama daha fazlası değil.

[Sorun Tanımı] Bazen, bakımın duygusal yönüyle ilgilenmek istemediğim için bakımdan geri çekildiğimi fark ediyorum. Organize edilmesi gereken şeylerle ilgileniyorum, örneğin tıbbi bakım, ancak annemin duygusal ihtiyaçlarını başkalarına bırakıyorum.

[Sonuç] Bakımın duygusal yönüyle başa çıkmakta zorlanıyorum. Gerekeni yapıyorum, ancak gerçekten bakımın içine aktif bir şekilde dahil olmuş gibi hissetmiyorum. Bakıma olan rolümü nasıl daha net tanımlayabilirim, aynı zamanda aşırı yüklenmiş hissetmeden?

Persona 9: Demans ve bakım konusunda bilgili olan, bu nedenle yalnızca profesyonellere güvenen ve kendisini adanmış bir bilgi aracısı olarak gören bir kişi

Hikaye paketine genel bakış

Hikaye	Konu
Hikaye 9.1	Giriş
Hikaye 9.2	Uzmanlara güven
Hikaye 9.3	Sorumluluk nedeniyle aşırı yüklenme
Hikaye 9.4	Kültürel beklentiler ve bakım
Hikaye 9.5	Bilgi ve destek

Persona 9: Demans ve bakım konusunda bilgili olan, bu nedenle yalnızca profesyonellere güvenen ve kendisini adanmış bir bilgi aracısı olarak gören bir kişi

Hikaye 9.1: Giriş

[Giriş] Annem, demans hastalığına yakalanmış ve ben onun başlıca bakım vericisiyim. Hastalık ve bakım konusunda derinlemesine bilgi edinmek için çok çaba sarf ettim çünkü onun en iyi bakımı almasını sağlamak istiyorum. Bakımı söz konusu olduğunda yalnızca profesyonel uzmanlara güveniyorum – doktorlar, hemşireler ve uzman danışmanlık hizmetleri.

[Sorun Tanımı] Bakım söz konusu olduğunda diğer aile üyelerine güvenmekte zorlanıyorum. Demans ve bakım hakkında çok şey biliyorum ama çoğu zaman başkalarının yeterince bilgiye sahip olmadığını hissediyorum. Bazen ailemden tavsiyeler alıyorum, ama annemin neye ihtiyacı olduğunu gerçekten anlayıp anlamadıklarından emin olamıyorum. Onun ihtiyaçlarını elimden geldiğince karşılamaya çalışıyorum, ama her şeyi yönetmek çok zor.

[Sonuç] Her şeyi tek başıma halletmeye çalışmanın beni bunaltığını fark ediyorum. Ama dışarıdan yardım alsam da, kimseye tam olarak güvenmiyorum. Kontrolü elinde tutmak ve her şeyin doğru yapıldığından emin olmak istiyorum. Başkalarına daha fazla güvenmeyi öğrenirken kontrolü kaybetmemek için ne yapabilirim?

Persona 9: Demans ve bakım konusunda bilgili olan, bu nedenle yalnızca profesyonellere güvenen ve kendisini adanmış bir bilgi aracısı olarak gören bir kişi

Story 9.2: Uzmanlara güven

[Giriş] Doktorlar, hemşireler ve demans danışmanlık hizmetleri ile düzenli olarak iletişime geçiyorum, böylece her zaman en iyi bilgi ve desteği alacağımızdan emin olabiliyoruz. Ailemde bakım konusunda karar veren kişi benim çünkü en fazla deneyimim ve bilgim var. Diğer aile üyeleri de bunu biliyor ve bu yüzden bana güveniyorlar. Bakım sorumluluğunu sevinçle bana devrediyorlar.

[Sorun Tanımı] Diğer yandan, çoğu zaman neden profesyonel yardıma bu kadar ısrarla başvurduğumu anlamıyorlar. Benim, dışarıdan destek almadan da bakım sürecini yönetebileceğimi düşünüyorlar. Ancak ben biliyorum ki demans, uzmanların çok önemli olduğu bir hastalık. Bilmediğim çok şey var ve annemin yanlış bir tedavi almasını riske atmak istemiyorum.

[Sonuç] Ailemi, profesyonel yardımın gerekli olduğu konusunda ikna etmek bazen zor oluyor. Ancak ben, uzmanların daha fazla bilgiye sahip olduklarına ve annem için neyin önemli olduğunu daha iyi bildiklerine dair görüşümden vazgeçmiyorum. Aileme, profesyonel yardıma neden bu kadar ısrarla başvurduğumu daha iyi nasıl açıklayabilirim?

Persona 9: Demans ve bakım konusunda bilgili olan, bu nedenle yalnızca profesyonellere güvenen ve kendisini adanmış bir bilgi aracısı olarak gören bir kişi

Hikaye 9.3: Sorumluluk nedeniyle aşırı yüklenme

[Giriş] Annemin başlıca bakım vericisi olduğum için, çoğu sorumluluğu ben taşıyorum. Her şeyi organize ediyorum – doktora gitmekten günlük bakım görevlerine kadar. Bazen her şeyin sadece bana bağlıymış gibi hissediyorum. Bunu yönetmek çok zor, özellikle kendi ailem ve işimle birlikte.

[Sorun Tanımı] Çoğu zaman her şeyin kontrolünü elinde tutmam gerektiğini hissediyorum, böylece her şeyin doğru gittiğinden emin olabilirim. Ancak bazen aşırı yüklenmiş hissediyorum. Bakım görevim ile kendi hayatım arasında bir denge kurmakta zorlanıyorum. Anneme her şeyi vermek istiyorum ama kendime zaman ayırmak neredeyse imkansız.

[Sonuç] Her şeyi bir arada tutmak sürekli bir zorluk. Ama annemi en iyi şekilde bakımını sağlamak konusunda kararlıyım, ne kadar zor olsa da. Bakımı, kendimi kaybetmeden nasıl daha iyi organize edebilirim?

Persona 9: Demans ve bakım konusunda bilgili olan, bu nedenle yalnızca profesyonellere güvenen ve kendisini adanmış bir bilgi aracısı olarak gören bir kişi

Story 9.4: Kültürel beklentiler ve bakım

[Giriş] Ailemde, yaşlı nesillere bakma konusunda güçlü bir gelenek vardır. Bu geleneği anlıyorum, ama aynı zamanda demansın, herkesin anlamadığı tamamen farklı bir bakım türü gerektirdiğini fark ettim. Kültürel beklentilere dikkat etmeye çalışıyorum, ancak annemin bakımının farklı bir düzeyde yapılması gerektiğini biliyorum.

[Sorun Tanımı] Ailemde sürekli olarak, annemi belirli bir şekilde bakımını yapmamı bekleyen durumlar oluyor. Ama bunun her zaman en iyi çözüm olmadığını biliyorum. Örneğin, annem artık kendine yardım edemediğinde bazı geleneksel bakım ritüelleri işe yaramayacak. Bu dengeyi bulmaya çalışıyorum – bir yandan geleneklere saygı göstermek, diğer yandan doğru bakımı sağlamak.

[Sonuç] Hem ailemin geleneksel değerleriyle hem de annemin gerçekten ihtiyacı olan şeyle bu iki dünyayı birleştirmek zor. Ama her iki yönün de en iyisini yapmaya çalışıyorum. Ailemin kültürel beklentileri ile annemin ihtiyaçlarını nasıl uyumlu hale getirebilirim?

Persona 9: Demans ve bakım konusunda bilgili olan, bu nedenle yalnızca profesyonellere güvenen ve kendisini adanmış bir bilgi aracısı olarak gören bir kişi

Story 9.5: Bilgi ve destek

[Giriş] Demans ve bakım hakkında çok şey bildiğim için bu bilgiyi başkalarıyla da paylaşmaktan memnuniyet duyuyorum. Annemin neye ihtiyacı olduğunu ve nasıl doğru şekilde bakım yapılması gerektiğini herkesin anlamasının önemli olduğunu düşünüyorum. Ama bazen, dışarıdan destek almanın çok yardımcı olabileceğini bildiğim halde, kendime yardım almakta zorlanıyorum.

[Sorun Tanımı] Demans hakkında çok konuşuyorum, ama kimsenin gerçekten ne kadar zor olduğunu anlamadığını hissediyorum. Çok şey bilsem de her şeyi tek başıma yapamam. Ancak, bana sunulan yardımı kabul etmekte de zorluk yaşıyorum çünkü diğer kişilerin de bu kadar bilgiye sahip olup olmadığından emin olamıyorum.

[Sonuç] Bilgi paylaşmak ile yardım kabul etmek arasında sürekli bir denge kurmak zor. Ama destek almam gerektiğini biliyorum, bunu kabul etmek bana zor gelse de. Belki bilgimi daha çok paylaşmalı ve böylece başkalarının benim seviyemde bilgi sahibi olduğunu bildiğimde de destek alabilmeliyim. Bilgimi paylaşmayı ve beni rahatlatacak yardımı kabul etmeyi nasıl öğrenebilirim?
